# Coordination Chemistry of Boron Subphthalocyanine With BINOL Derivatives: Unexpected Oxidation to Oxa[4]Helicene and Properties of the Resulting Axially Coordinated Complexes Containing Oxa[4]Helicene and/or BINOL Derivatives

**DOI:** 10.1002/chem.71412

**Published:** 2026-07-25

**Authors:** Shafikul Islam, Kazuyuki Asahi, Hiroyuki Nishikawa, Daiki Tauchi, Masashi Hasegawa, Ken‐ichi Sugiura

**Affiliations:** ^1^ Department of Chemistry, Graduate School of Science Tokyo Metropolitan University Hachi‐Oji Tokyo Japan; ^2^ Department of Chemistry Jahangirnagar University Dhaka Bangladesh; ^3^ Graduate School of Science and Engineering Ibaraki University Mito Ibaraki Japan; ^4^ Department of Chemistry, Graduate School of Science Kitasato University Sagamihara Kanagawa Japan

**Keywords:** axial‐coordination, BINOL, boron subphthalocyanine, CISS, oxa[4]helicene

## Abstract

In the course of our research on axially coordinated metalloporphyrinoids with stable bonding based on the hard and soft acid–base (HSAB) theory, we focused on boron subphthalocyanine because of the hard acid character of the boron(III) center. 1,1′‐Bi‐2‐naphthol (BINOL) was selected as an axial ligand to introduce chirality into the otherwise achiral subphthalocyanine. Surprisingly, treatment of unsubstituted BINOL with boron subphthalocyanine resulted in the formation of a complex coordinated to an oxa[4]helicene derivative formed via a ring‐closure reaction of BINOL. In contrast, treatment of the monomethyl ether of BINOL with boron subphthalocyanine afforded the expected axially coordinated complex. Based on several control experiments, we propose a reaction mechanism for the formation of the oxa[4]helicene involving coordination of BINOL, followed by deprotonation and racemization, and subsequent intramolecular ring‐closure via an ionic process. To characterize and elucidate the fundamental properties of the resulting complexes, single‐crystal x‐ray diffraction, NMR spectroscopy, UV–vis absorption spectroscopy, and electrochemical studies were carried out. Furthermore, we investigated the chirality‐derived properties of the complexes, and the chirality‐induced spin selectivity (CISS) effect was evaluated using magnetic conductive atomic force microscopy (mc‐AFM), revealing a large spin polarization.

## Graphical abstract

Treatment of BINOL with a boron subphthalocyanine derivative resulted in the formation of a complex coordinated to an oxa[4]helicene derivative, which is generated via a ring‐closure reaction of BINOL. In contrast, treatment of the monomethyl ether of 1,1′‐bi‐2‐naphthol (BINOL) afforded the expected axially coordinated complex. A plausible reaction mechanism, as well as the CISS properties of the complexes, are discussed.

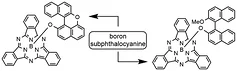

## 1 Introduction

The chemistry and science of metalloporphyrins and related materials have attracted significant attention for over a century [1, 2, 3, 4, 5]. To elucidate the reaction mechanisms of metalloporphyrinoid‐containing proteins and biomolecules, as well as to uncover novel properties, various chemical modifications of porphyrins have been explored. Among these strategies, the introduction of axial ligands has been relatively uncommon due to the instability of metal–ligand coordination bonds. For example, axial ligands often dissociate readily in solution [6]. If axial ligand bonding with kinetic stability comparable to that of covalent bonds could be achieved, it would represent a new avenue for chemical modification. Against this background, we have focused our attention on axial ligation in metalloporphyrins [7, 8, 9].

Recently, we discovered that metals with hard‐acid character, such as gallium (Ga^3^^+^) and tin (Sn^4^^+^), form kinetically stable bonds with hard‐base ligands such as phenolates [7, 8, 9], in accordance with the hard and soft acid–base (HSAB) theory [10]. The resulting complexes exhibit remarkable kinetic stability. For instance, the phenolato complex of gallium Ga(III) porphyrin ([Ga^3^^+^Por]) produced a clear molecular ion signal in atmospheric pressure chemical ionization mass spectrometry (APCI‐MS), without any degradation of the axial ligand during ionization [8, 9]. These complexes are sufficiently stable to be handled under ambient conditions and to undergo conventional synthetic operations, such as recrystallization from hot solvents. To expand the scope of this chemistry, replacing the metal center with another hard‐acid species remains a challenge. Boron (B^3^^+^), another common hard‐acid element [10], is a promising candidate. Boron subphthalocyanine (B^3^^+^SubPc), **1**, is a well‐known B^3^^+^‐containing porphyrinoid compound, as the small ionic radius of B^3^^+^ necessitates a contracted ligand framework [11, 12, 13, 14]. Several B^3^^+^SubPc complexes with oxygen‐containing axial ligands have been reported to date [11, 12, 13, 14, 15, 16, 17].

In this study, we focus on the introduction of the chiral axial ligand 1,1′‐bi‐2‐naphthol (BINOL) to form compound **1a**, aiming to impart chirality to B^3^^+^SubPc based on our recent discovery of the BINOL‐coordinated Ga^3^^+^Por complex [9]. Unexpectedly, oxidation of the coordinated BINOL led to the formation of an oxa[4]helicene derivative (**2**) [18] and the isolation of the corresponding subphthalocyanine **1b**. Herein, we report and discuss the axial coordination chemistry of boron subphthalocyanine with BINOL and its oxidation derivative, including control experiments that support the proposed reaction mechanism for the formation of **1b**. The natures of the formed complexes were characterized by conventional spectroscopic techniques. Furthermore, we attempted to observe the chirality‐derived properties of the complexes, such as circular dichroism (CD) and the chirality‐induced spin selectivity (CISS) effect [19, 20]. The CISS effect was evaluated using magnetic conductive atomic force microscopy (mc‐AFM), revealing a large spin polarization for chiral **1c**.

## 2 Results and Discussions

### 2.1 Ligation Reaction of BINOL on Boron Subphthalocyanine Chloride

In 2003, Kobayashi and coworkers reported BINOL‐coordinated boron subphthalocyanine **3** in racemic form under harsh reaction conditions, i.e., boiling 1,2,4‐trichlorobenzene [17]. In this study, the target complex **1a** was attempted to be synthesized via a ligand exchange reaction of **1e**, derived from **1d**, using the method developed by González‐Rodríguez and Torres [21] under mild conditions to avoid racemization of optically pure BINOL. After the formation of triflate **1e**, *rac‐*BINOL was initially added to the reaction mixture. Thin‐layer chromatography clearly indicated the formation of a new product, which was successfully isolated by conventional SiO_2_ column chromatography without any degradation of the ligand (*vide infra*). The ^1^H NMR spectrum showed well‐resolved signals corresponding to the BINOL moiety (Figure S1), reflecting the ring‐current effects of the subphthalocyanine π‐system (*vide infra*). Interestingly, the integration values suggested the loss of a single proton from the BINOL moiety.

Due to the good crystallinity of the product, a single crystal suitable for x‐ray diffraction was obtained. The molecular structure, shown in Figure 2 (*vide infra*), revealed that BINOL had undergone a ring‐closure reaction to form the oxa[4]helicene derivative **2**, resulting in complex **1b**. Based on this structural information, all ^1^H NMR signals of **1b** were assigned with the aid of two‐dimensional spectra such as ^1^H–^1^H COSY and NOESY (Figure S2 and S3). The high‐resolution atmospheric pressure chemical ionization (APCI) mass spectrum further supported the formation of **1b**, showing the loss of a single hydrogen atom (Figure S4).

To elucidate the mechanism of this unique ring closure reaction, five control experiments (CE) were conducted:

1. CE#1: The reaction was performed in the absence of light, based on the hypothesis that photoinduced electron transfer from BINOL to the electron‐deficient subphthalocyanine might initiate the ring closure via a cation radical intermediate. Despite careful handling, including chromatography under dark conditions, **1b** was still obtained.
2. CE#2: The reaction was carried out under 200 W tungsten lamp irradiation. No significant changes were observed.
3. CE#3: An excess of BINOL was stirred with the reaction mixture to test whether **1b** or **1e** could catalyze the formation of oxa[4]helicene **2**. No reaction occurred with the unreacted BINOL.
4. CE#4: Optically pure BINOL enantiomers were used; however, racemic **1b** was obtained in all cases. This behavior was confirmed by single‐crystal x‐ray diffraction analysis of the product, which revealed that the crystals crystallize in the centrosymmetric space group P2_1_/c. Although oxa[4]helicene **2** is known to racemize easily due to minimal steric hindrance [22], the attachment of the bulky subphthalocyanine at the terminus of **1b** was expected to prevent racemization. Nevertheless, the reaction yielded a racemic product.
5. CE#5: BINOL monomethyl ether [23, 24] was used, resulting in the formation of the expected complex **1c** without ring closure of the BINOL moiety.

Based on these control experiments, the following mechanism is proposed for the reaction of (*R*)‐BINOL (Scheme 1):

1. Step 1: Ligand exchange of 1e (derived from 1d) with (R)‐BINOL to afford (R)‐1a.
2. Step 2: Formation of a weak charge‐transfer interaction between the naphthol moiety and the subphthalocyanine.
3. Step 3: Deprotonation to yield (R)‐4.
4. Step 4: Rotation and/or racemization of the NP‐II moiety to generate (S)‐4.
5. Step 5: Intramolecular attack of the phenolato oxygen of NP‐II on NP‐I, producing (M)‐1b and (P)‐1b from (S)‐3 and (R)‐3, respectively.

**SCHEME 1:**
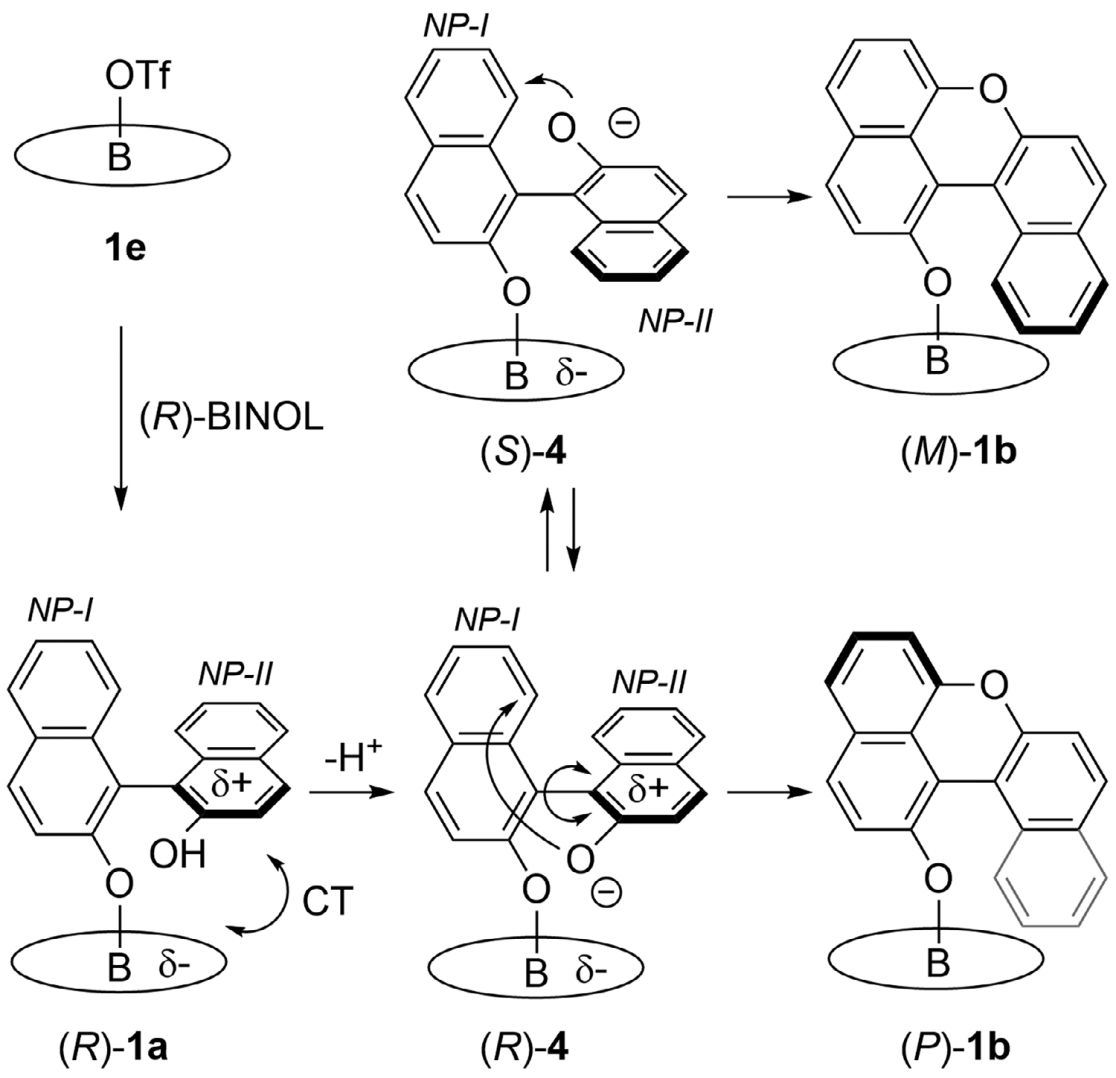
Plausible reaction mechanism for the formation of oxa[4]helicene (**2**)–coordinated boron subphthalocyanine **1b**.

The result of CE#3 suggests that ring closure occurs only when BINOL is coordinated to the subphthalocyanine, and not in its free form. CE#4 indicates that racemization occurs readily at room temperature, consistent with the known behavior of deprotonated BINOL in basic media [25]. CE#1 and CE#2 suggest that the ring closure proceeds via an ionic intermediate rather than through a photoinduced cation radical. Accordingly, a proton‐release process facilitated by charge‐transfer interaction is proposed. The result of CE#5 supports the necessity of both hydroxyl groups for ring closure. The possibility of racemization of the formed oxa[4]helicene in its dissociated state—i.e., dissociation, racemization, and re‐ligation—is ruled out due to the stability of the B─O bond, with no dissociation observed during the reaction (*vide infra*).

It is worth noting that the authors are particularly interested in the stability of the axial bonding. As described above, the complexes remained stable during SiO_2_ column chromatography. Notably, the ring closure product **1b** was fully stable under acidic conditions, including treatment with *dilute* or *concentrated* HCl, which typically induces dissociation of axial ligands in Ga^3^^+^ porphyrins. However, treatment with *concentrated* H_2_SO_4_ led to decomposition of the subphthalocyanine core, and attempts to isolate compound **2** were unsuccessful.

### 2.2 Structures of the Complexes and NMR Behaviors

Fortunately, we obtained the crystal structures of two unique complexes, **1b** and **1c** (Figure 2 and 3, respectively). In complex **1b**, it is evident from Figure 2 that BINOL has been transformed into an oxa[4]helicene (*vide infra*). Important structural parameters are summarized in Table 1, along with values from structurally related subphtahlocyanines coordinated by phenol or naphthol. Similar to previously reported complexes, the subphthalocyanine ligand adopts a bowl‐shaped geometry [11, 12, 13, 14]. The boron atom forms a distorted tetrahedral and/or trigonal pyramidal geometry, with three nitrogen atoms of the subphthalocyanine forming the base. The average B─N bond lengths (*d*(B─N)) in **1b** and **1c** are nearly identical to those reported for similar complexes. The *d*(B─O) are 1.4530(18) Å in **1b** and 1.434(3) Å in **1c**, both of which fall within the range reported for complexes **1f–1h** (Table 1). Although the reason is not yet clear and, to our knowledge, has not been previously discussed, it is noteworthy that these *d*(B─O) are shorter than the sum of the covalent radii of boron and oxygen (1.48 Å) [26]. In subphthalocyanine complexes coordinated via oxygen atoms, the distortion of the coordination geometry is typically evaluated using the B─O─C angle (∠BOC). The ∠BOC values for **1b** and **1c** are 115.8(1)° and 122.1(2)°, respectively, which are comparable to previously reported values.

**TABLE 1:** Selected bond distances and other structural parameters for the reported subphthalocyanine complexes, including complexes 1b and 1c.

Complex	*d*(B‐O) [Å]	*d*(B‐N) [Å], (average)	∠BOC [degree]	CSD #^a^	Reference
**1b**	1.4530(18)	1.4913(19)	115.8(1)	2424770	This work
**1c**	1.434(3)	1.500(3)	122.1(2)	2424776	This work
**1f**	1.443(2)	1.498	115.5(1)	IJEYUS	[15]
**1g**	1.455	1.493	121.2	JORDUR	[16]^b^
**1h**	1.442	1.497	^c^	JORFAZ	[16]^b^
**3**	1.432(3)	1.495(3)	120.6(2)	EHOQUN	[17]^d^

^a^ Deposition number at the Cambridge Crystallographic Data Centre (CCDC).

^b^ Standard deviation values were not reported.

^c^ There is disorder in the ligand moiety, and since its occupancy was not reported, the value could not be determined from the CIF data.

^d^ Because BINOL binds to two subphthalocyanines unequivalently, the average values are presented here.

**FIGURE 1:**
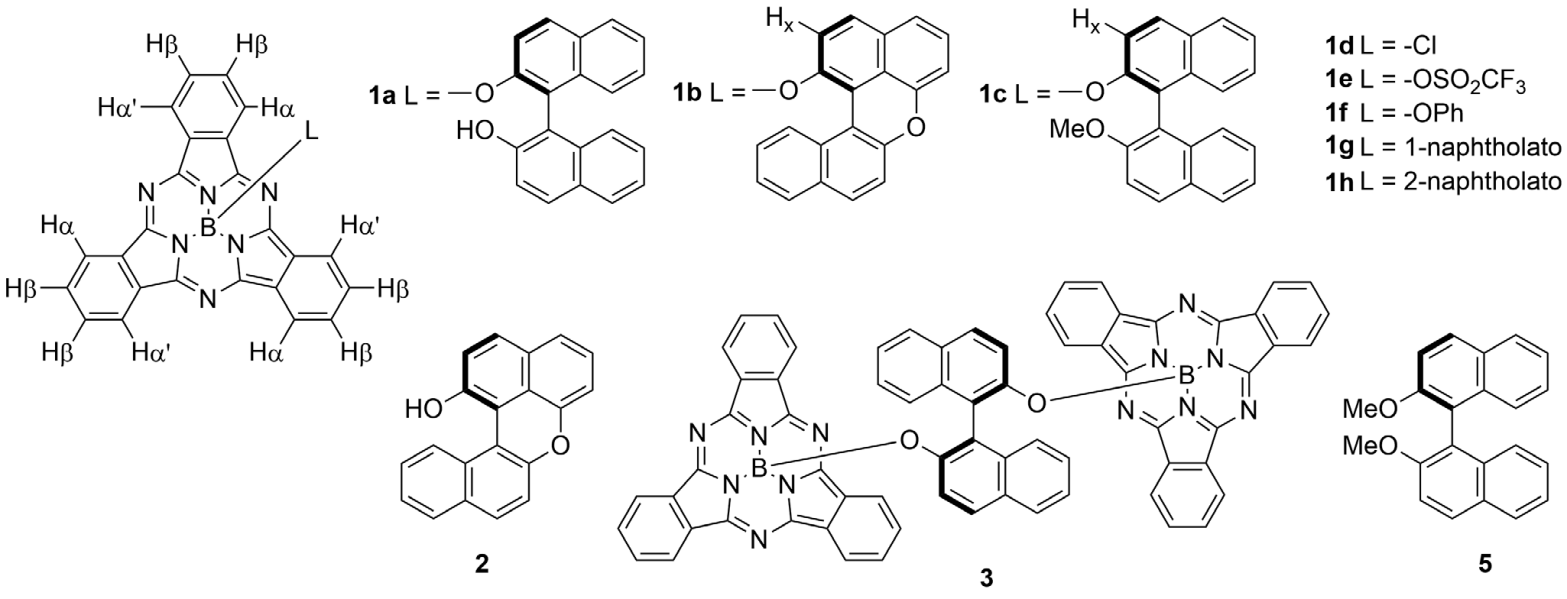
Structures discussed in this paper and their related complexes. Chiral compounds (**1a**, **1b**, **1c**, **2**, **3**, and **5**) are represented by their corresponding (*R*)‐ or (*S*)‐configured structures.

A distinctive feature of **1b** is its helicene structure, bearing a large subphthalocyanine moiety at one terminus. The obtained **1b** is a *racemic* mixture, and the crystal belongs to the *P*2_1_/_c_ space group. One enantiomer (the (*P*)‐form) is shown in Figure 2, and the other enantiomer is generated by an inversion operation. The degree of helicene‐twisting is discussed in terms of the dihedral angle formed between the terminal benzene rings, *Ph‐I* and *Ph‐IV*. This angle in **1b** is 36.6°, significantly larger than that of [4]helicene (26.7°) [27], likely due to the attachment of the bulky subphthalocyanine to the oxa[4]helicene. This sterically hindered structure effectively eliminates the possibility of racemization and is considered a promising new motif for optically active [4]helicenes (an attempt to achieve optical resolution of racemic **1b** is discussed later).

Previously, we reported the first example of a metal porphyrin coordinated by BINOL. Due to the bowl‐shaped geometry of subphthalocyanine, direct comparison with metal porphyrins is difficult. The least‐squares plane of BINOL's *NP‐I* binds to the subphthalocyanine plane defined by three pyrrole‐nitrogens at an angle of 39.9°. The most important structural feature of this complex is the dihedral angle between *NP‐I* and *NP‐II*, which is obtained as an obtuse angle, 112.5°. It has long been suggested that the dihedral angle in BINOL derivatives influences their chiroptical properties, but compounds that actually exhibit such obtuse angles have been limited [28]. This complex is expected to serve as a key compound for evaluating the chiroptical properties imparted by BINOL with an obtuse dihedral angle (*vide infra*).

^1^H NMR spectroscopy also provides valuable structural information for **1b** and **1c**. The observed behavior reproduces trends seen in gallium porphyrins, with BINOL‐derived well‐resolved signals shifted to the high‐field region (8.0–5.5 ppm). The protons closest to the subphthalocyanine ring H_x_ (see the structure of **1b** and **1c** in Figure 1), as revealed by x‐ray analysis, were assigned to the signal observed at the highest field, 5.73 and 5.59 ppm for **1b** and **1c**, respectively. Subphthalocyanine signals also showed significant changes upon BINOL coordination. The fused benzene ring signals exhibit an *AA′BB′* spin multiplicity pattern. However, in example **1c** (Figure 4), although the six H_β_ protons are magnetically equivalent, the H_α_ and H_α_′ protons—three each—give rise to signals corresponding to three magnetically non‐equivalent protons. This phenomenon can be reasonably explained by assuming that the chiral axial ligand undergoes free rotation at room temperature. We previously reported the similar behavior in BINOL coordinate GaPor [9].

**FIGURE 2:**
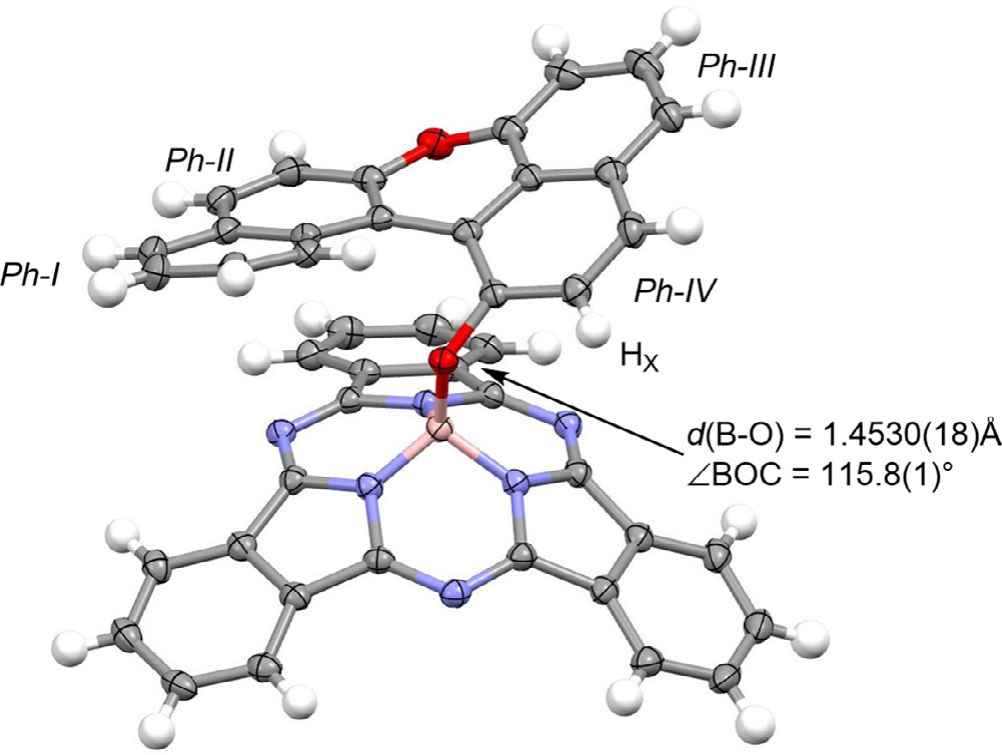
Molecular structure of complex **1b**. Thermal ellipsoids are shown at the 50% probability level. This crystal crystallizes in the centrosymmetric space group *P*2_1_/c, indicating that both (*P*)‐ and (*M*)‐enantiomers are present in the crystal. The stereoisomer corresponding to the structure shown in the figure, namely the (*M*)‐oxa[4]helicene derivative, is related to the depicted structure through a crystallographic inversion center.

**FIGURE 3:**
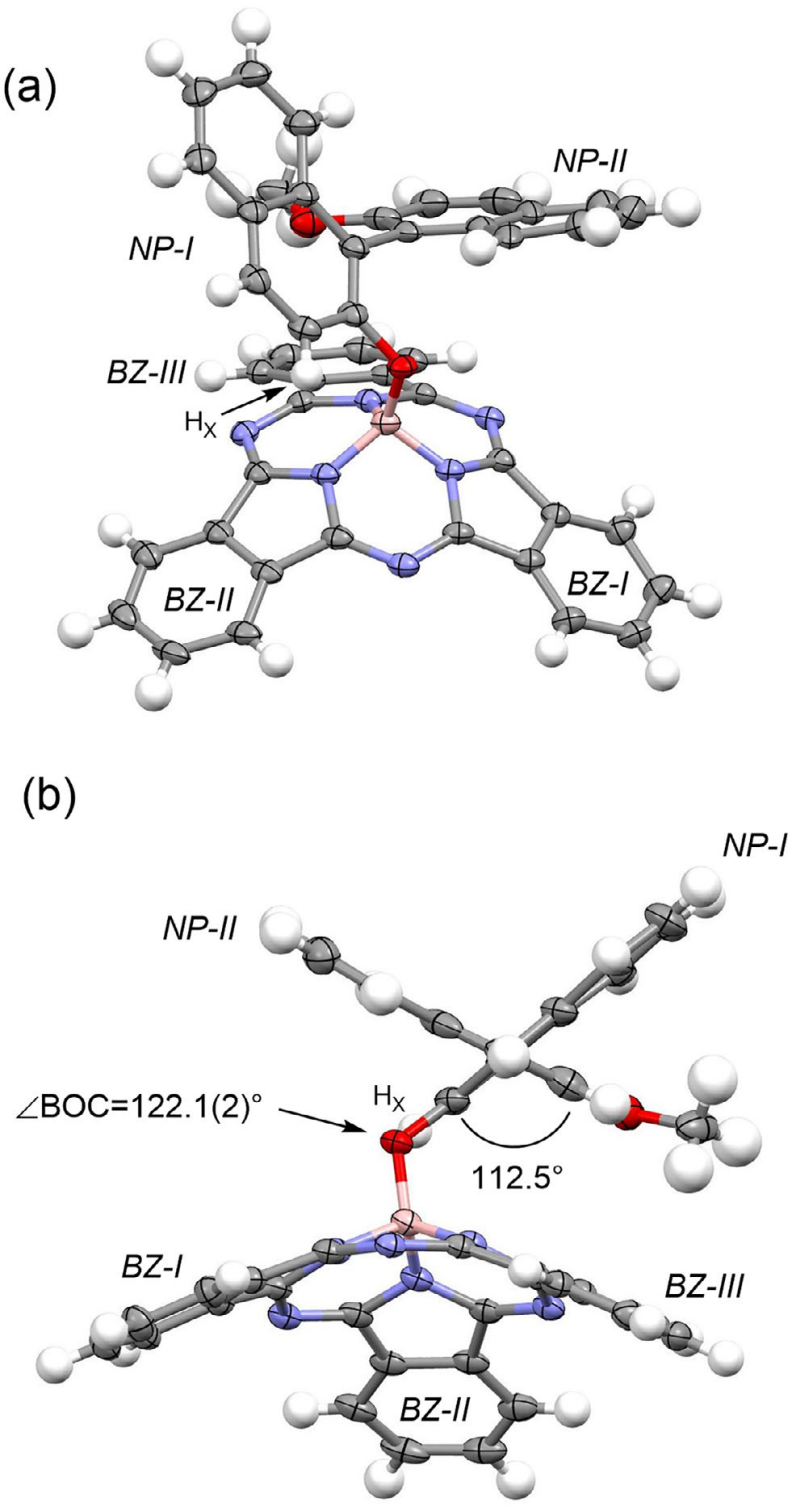
Molecular structure of complex **1c**. Thermal ellipsoids are shown at the 50% probability level. (a) Perspective view and (b) side view along the chiral axis of BINOL. The dihedral angle between the least‑squares planes defined by the ten naphthalene carbon atoms is obtuse, 112.5°.

**FIGURE 4:**
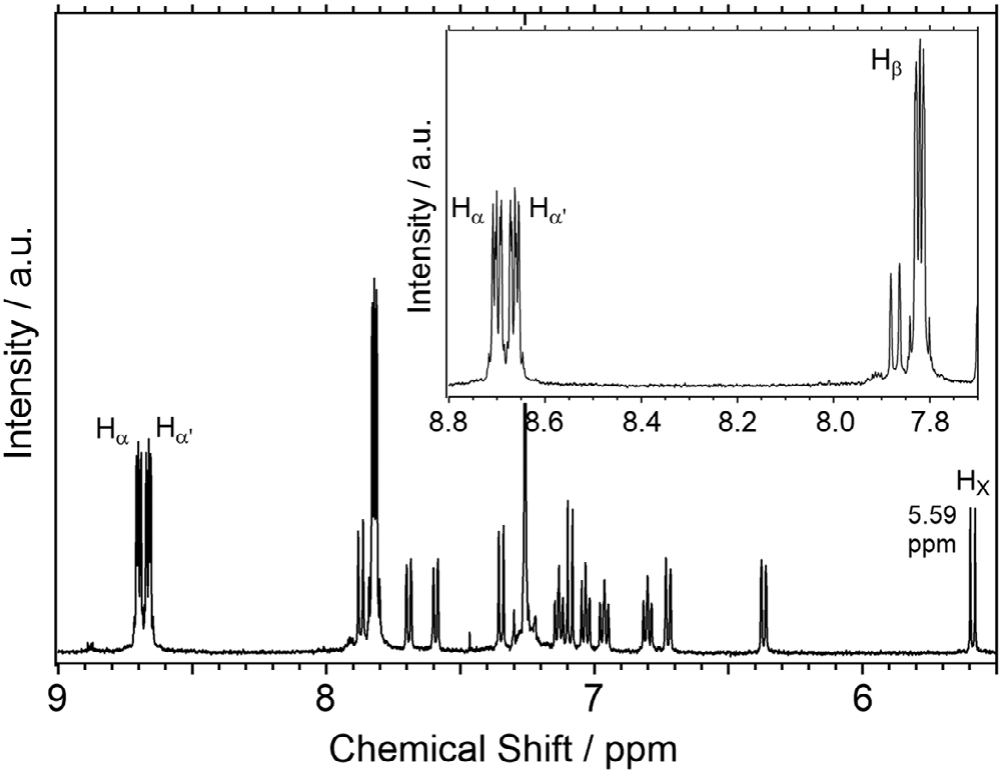
^1^H NMR spectrum of **1c** (in CDCl_3_).

### 2.3 Electrochemical Studies

In the previous section, we proposed a reaction mechanism that includes a proton dissociation step induced by charge‐transfer interactions between subphthalocyanine and *NP‐I* of BINOL. To verify this hypothesis, we conducted electrochemical studies (see Section S2). It is well known that the LUMO of subphthalocyanine is stabilized [29], and both complexes **1b** and *rac*‐**1c** exhibited reversible reduction waves at −1.57 and −1.56 V (vs. ferrocene/ferrocenium in CH_2_Cl_2_), respectively. Regarding the oxidation processes at 0.46 and 0.50 V for **1b** and *rac*‐**1c**, respectively. These oxidation waves could be attributed to the axial ligands. Therefore, effective charge‐transfer interactions occur between the axial ligand, BINOL and/or oxa[4]helicene, and subphthalocyanine. It should be noted that the oxidation process attributable to the oxa[4]helicene moiety was quasi‐reversible, whereas that attributable to BINOL was irreversible.

### 2.4 Absorption, Emission, and Other Spectroscopic Studies

The absorption and emission spectra of complexes **1b** and *rac*‐**1c**, as well as the CD spectrum of (*R*)‐ and (*S*)‐**1c**, are shown in Figure 5. Both complexes exhibit characteristic absorption spectra of boron subphthalocyanine, with a strong Q‐band observed around 560 nm and a Soret band around 300 nm [11, 12, 13, 14]. The emission was observed at 565 nm as a mirror image of the Q‐band. The absorption below 360 nm for both complexes is considered to originate from the axial ligands. Based on TD‐DFT calculations (Section S3 and S4), the broad absorption near 400 nm in **1b** was attributed to the oxa[4]helicene moiety.

**FIGURE 5:**
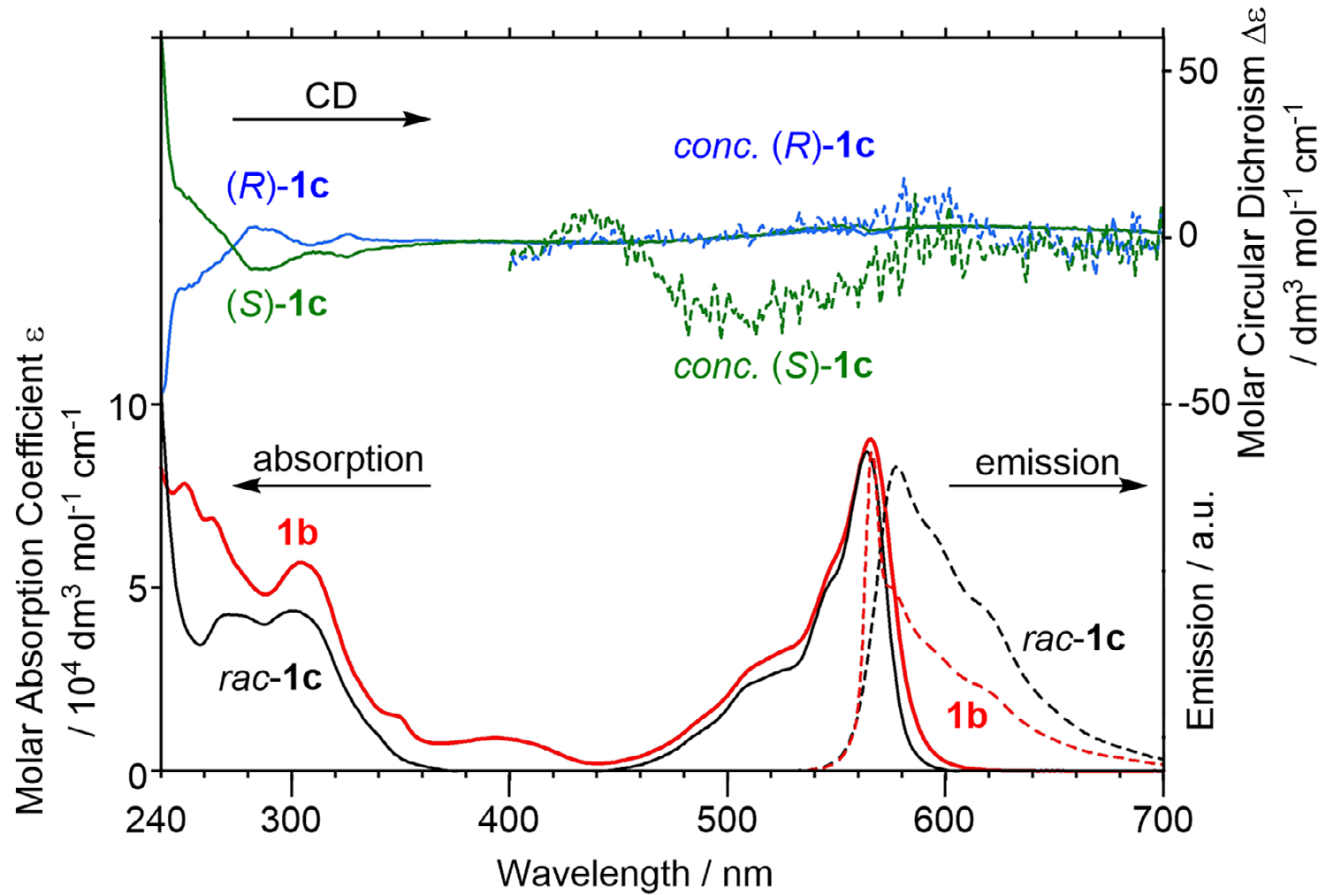
(bottom) Absorption and emission spectra of **1b** and *rac*‐**1c**. (top) CD spectra of optically pure (*R*)‐ and (*S*)‐**1c**.

CD measurements of (*R*)‐ and (*S*)‐**1c** revealed mirror‐image Cotton effects in the absorption region of BINOL, 260–360 nm, with the (*R*)‐**1c** showing a positive sign and the (*S*)‐**1c** a negative sign. It is known that the CD spectrum of BINOL varies depending on the dihedral angle (*θ*) between the two naphthalene rings [28]. As mentioned earlier, the dihedral angle of BINOL in **1c** was obtuse, 112.5°. Therefore, we performed CD simulations for model compound dimethoxy BINOL **5** by varying the angle *θ* (see Section S5). As a result, the spectral shape changed significantly near the most stable structure at *θ* = 90°, and the experimental spectrum was successfully reproduced when *θ* was set to 107.9°, which is close to the angle observed in **1c** (112.5°).

It is worth to introduce the Q‐band behavior of **1c**. Under the conventional condition, no significant CD signal was observed in this region, however, concentrated solution gave weak signal with a poor signal‐to‐noise ratio, however, significant mirror image signals, i.e., the (*R*)‐**1c** showing a positive sign and the (*S*)‐**1c** a negative sign. Two possibilities are considered; one possibility is that the signal was an artifact caused by the measurement instrument, and the other is that it is a very weak signal resulting from chirality transfer from BINOL to subphthalocyanine based on the Kuhn–Kirkwood coupled oscillator model and/or the CD stealing mechanism [30]. In support of the latter possibility, TD‐DFT calculations for **1c** indicate a significant CD transition in the Q‐band (Figure S11). Furthermore, a similar phenomenon was observed in our previously reported gallium porphyrin complex coordinated with BINOL [9].

### 2.5 Chirality‐Induced Spin Selectivity (CISS)

To further reveal the nature of the complexes attributable to their chirality, we evaluated chirality‐induced spin selectivity (CISS) for (*R*)‐**1c** and (*S*)‐**1c**. Although various methods have been reported for spin‐selective transport [19, 20] we adopted magnetic‐conductive atomic force microscopy (mc‐AFM) because of its experimental simplicity, reliability, and compatibility with a wide range of organic compounds [19]. The complexes were deposited on highly oriented pyrolytic graphite (HOPG). The conductivity was measured using a magnetized AFM‐tip (experimental details are shown in *SI* Section §7). Conductivity measurements were performed at various positions on the substrate, with a total of 40 measurements. After reversing the magnetization direction of the tip, an additional 40 measurements were carried out. All of the measurements are shown in pale color in Figure 6a,b. Spin‐selective electron transport is quantified by the spin polarization (SP) value, defined as the relative difference between the currents measured under the two magnetization configurations, i.e., SP = (*I*_up_ − I_down_)/(I_up_ + I_down_)] × 100. Average SP values of approximately ±60% ± 5% were obtained for (*R*)‐ and (*S*)‐**1c**, respectively. These spin filtering is comparable to and/or significantly larger those reported for other chiral subphthalocyanine complexes, 35%–40% [31].

**FIGURE 6:**
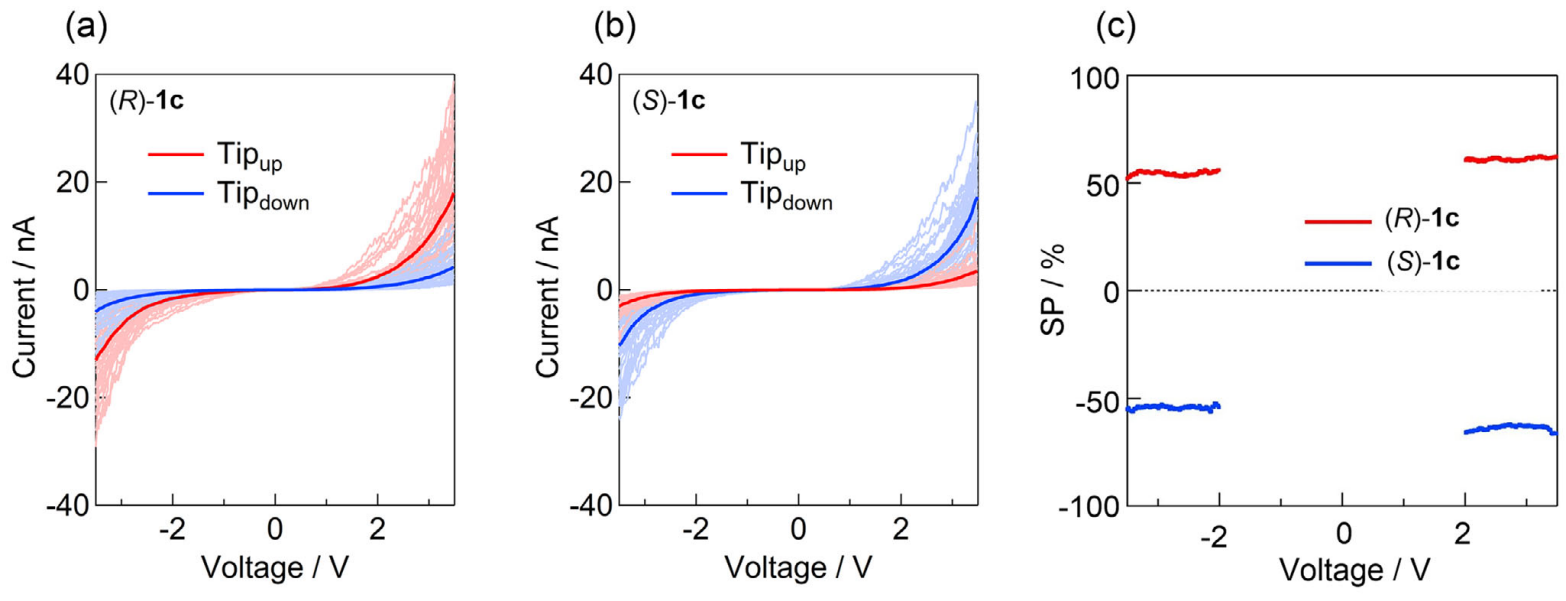
Spin‑dependent transport properties measured by mc‑AFM for (a) (*R*)‐**1c** and (b) (*S*)‐**1c**. All recorded current–voltage (I–V) curves are shown in pale colors, and their averages are shown as solid lines. (c) Spin polarization percentage (SP%) as a function of the applied bias is plotted in the regions above +2 V and below –2 V.

## 3 Conclusion

With the aim of preparing BINOL‐coordinated metalloporphyrins, novel boron subphthalocyanine complexes **1b** and **1c** were successfully synthesized and characterized. Control experiments enabled us to propose a plausible reaction mechanism for the formation of complex **1b**.

The serendipitously obtained complex **1b** can be regarded as an expanded helicene incorporating the subphthalocyanine framework. Owing to the presence of a bulky and strongly absorbing subphthalocyanine unit at its terminus, **1b** represents a compelling new molecular motif for optically active [4]helicenes.

In contrast, the optically active BINOL‐derived complex **1c** provides an opportunity to introduce chirality into various properties of subphthalocyanine reported previously, including optical characteristics and catalytic functions. Among these, we are particularly interested in molecule‐based electronics, where subphthalocyanine has been extensively studied as an electron‐accepting component in electron‐transport materials. Accordingly, complex **1c** is a promising candidate for chirality‐induced spin selectivity (CISS), and research in this direction is currently underway.

Overall, the ability to induce chirality through the simple introduction of an axial ligand represents an attractive strategy. Therefore, we will continue the synthesis and evaluation of related complexes.

## Conflicts of Interest

The authors declare no conflicts of interest.

## Supporting Information

Details of experimental procedures, characterization of product, theoretical studies, and the single‐crystal x‐ray analyses (CSD Deposition **1b**: 2424770 and **1c**: 2378335). Supporting information for this article is available on the WWW under https://doi.org/10.1002/ejoc/2024XXXXX.

## Supporting information




**Supporting File 1**: chem71412‐sup‐0001‐SuppMat.zip

## Data Availability

The data that support the findings of this study are available on request from the corresponding author. The data are not publicly available due to privacy or ethical restrictions.
