# Supplementary material for: Coordination Chemistry of Boron Subphthalocyanine With BINOL Derivatives: Unexpected Oxidation to Oxa[4]Helicene and Properties of the Resulting Axially Coordinated Complexes Containing Oxa[4]Helicene and/or BINOL Derivatives
Source: Chemistry. 2026 Jul 25;32(40):e71412. doi: 10.1002/chem.71412 (PMC13716020; doi:10.1002/chem.71412)
Supplement: Supplementary file 1 [file CHEM-32-e71412-s001.zip › REVISED_chem71412-sup-0001-SuppMat.docx]

Supporting Information

Coordination Chemistry of Boron Subphthalocyanine with BINOL Derivatives: Unexpected Oxidation to Oxa[4]helicene and Properties of the Resulting Axially Coordinated Complexes Containing Oxa[4]helicene and/or BINOL Derivatives

Shafikul Islam,^[a,b]^ Kazuyuki Asahi,^[c]^ Hiroyuki Nishikawa,^* [c]^ Daiki Tauchi,^[d]^ Masashi Hasegawa,^* [d]^, and Ken-ichi Sugiura^* [a]^

[a]	*Department of Chemistry, Graduate School of Science, Tokyo Metropolitan University, 1-1 Minami-Osawa, Hachi-Oji, Tokyo 192-0397, Japan*

[b]	*Department of Chemistry, Jahangirnagar University, Savar, Dhaka-1342, Bangladesh*

[c]	*Graduate School of Science and Engineering, Ibaraki University, 2-1-1 Bunkyo, Mito, Ibaraki 310-8512, Japan*

[d]	*Department of Chemistry, Graduate School of Science, Kitasato University, 1–15-1 Kitasato Minami-ku, Sagamihara, Kanagawa 252–0373, Japan*

§1 Synthesis

1-1 General

The following chemicals were prepared according to the literature. Toluene (Kanto Chemical Co., Ltd.) used in the complex formation reaction was distilled over P₂O₅ prior to use. All other chemicals were used as received. Methoxy-BINOL was also prepared according to the literature.^[1]^ Thin-layer chromatography (TLC) was performed using Merck/Supelco silica gel 60 F254 plates. Silica gel flash column chromatography was performed using a Silica Gel 60N (40–50 μm, spherical, neutral) from Kanto Chemical Co., Inc. An EARTH MAN CLT-120 clip light (200 W, Takagi Co., Ltd., Japan) was used as the light source in the light-irradiation control experiment (CE#2).

1-2 Synthesis of the complexes, **1b** and **1c**.

(General Procedure)

A mixture of chloroboronsubphthalocyanine **1d** (83.4 mg, 194 μmol, TCI Co., Ltd.), silver triflate (67.3 mg, 262 μmol, 1.4 equiv, TCI Co., Ltd.), and distilled toluene (3 mL) was purged with nitrogen and stirred at room temperature for 2 h. During the reaction, the color of the mixture changed from pink to violet. The progress of the reaction was monitored by TLC (*R*_f_ = 0.78 for **1d** and *R*_f_ = 0.04 for **1e** in toluene/ethyl acetate = 9:1). To avoid formation of the μ-oxo dimer of subphthalocyanine (*R*_f_ = 0.18), the reaction time at this stage should be minimized.

Subsequently, *rac*-methoxyBINOL (116.3 mg, 387 μmol, 2.0 equiv)^[1]^ and diisopropylethylamine (31.3 mg, 242 μmol, 1.25 equiv, TCI Co., Ltd.) were added, and the mixture was stirred at 50 °C for 14 h. This stage was also monitored by TLC (*R*_f_ = 0.72 for **1c**). The reaction was quenched, and the solvent was removed under reduced pressure. The residue was subjected to column chromatography on silica gel, eluting with toluene/ethyl acetate (9:1), to afford the crude product. Further purification by silica gel chromatography using the same eluent yielded compound **1c** as a pinkish-red solid (72.1 mg, 54%). The μ-oxo dimer of subphthalocyanine was also isolated as a byproduct (24.9 mg, 32%).

Similar reactions were carried out using the following reagents to afford **1b** and (*R*)- and (*S*)-**1c**.

**Table SI-1** Summary of complex formation reactions.

| **1d** | AgOTf | PhMe | BINOLs | iPr_2_NEt | Reaction time | Products  (yield) |
| --- | --- | --- | --- | --- | --- | --- |
| 86.3 mg  (1 eq.) | 66.8 mg  (1.3 eq.) | 3.0 mL | (*R*)-methoxy BINOL  120.7 mg  (2.0 eq.) | 31.3 mg  (1.3 eq.) | 16h  At 50 °C | (*R*)-**1c**  (73.1 mg, 53%)  μ-oxo dimer  (25.1 mg, 31%) |
| 90.2 mg  (1 eq.) | 69.8 mg  (1.3 eq.) | 3.0 mL | (*S*)-methoxy BINOL  126.3 mg  (2.0 eq.) | 33.8 mg  (1.3 eq.) | 16h  At 50 °C | (*S*)-**1c**  75.2 mg, 52%  μ-oxo dimer  (27.8 mg, 32%) |
| 52.2 mg  (1 eq.) | 40.0 mg  (1.3 eq.) | 4.0 mL | *rac*-BINOL  104.1 mg  (3.0 eq.) | 17.5 mg  (1.1 eq.) | 16h  At 40 °C | **1b**  44.5 mg, 54%  μ-oxo dimer  (15.2 mg, 31%) |
| 51.8 mg  (1 eq.) | 41.1 mg  (1.3 eq.) | 3.0 mL | *(R)*-BINOL  137.5 mg  (4.0 eq.) | 13.3 mg  (1.1 eq.) | 16h  At 40 °C | **1b**  43.2 mg, 53%  μ-oxo dimer  (14.8 mg, 30%) |
| 50.2 mg  (1 eq.) | 40.5 mg  (1.3 eq.) | 3.0 mL | *(*S*)* -BINOL  135.8 mg  (4.0 eq.) | 13.3 mg  (1.1 eq.) | 16h  At 40 °C | **1b**  41.8 mg, 53%  μ-oxo dimer  (14.6 mg, 31%) |

**1c**: ^1^H NMR (500 MHz, CDCl_3_) δ 8.71-8.66 (*AA’BB’*-spin system, 6H), 7.87 (d, *J* = 8.9Hz, 1H), 7.84-7.80 (*AA’BB’*-spin system, 6H), 7.69 (d, *J* =8.2, 1H), 7.59 (d, *J* = 8.0Hz, 1H), 7.34 (d, *J* = 9.0, 1H), 7.13 (dt, *J* = 6.6 and 1.0 Hz, 1H), 7.09 (d, *J* = 9.0 Hz, 1H), 7.03 (dt, *J* = 6.6 and 0.85 Hz, 1H), 6.96 (dt, *J* = 6.7 and 1.2 Hz, 1H), 6.80 (dt, *J* = 6.6 and 1.2 Hz, 1H), 6.72 (d, *J* = 8.7 Hz, 1H), 6.37 (d, *J* = 8.1 Hz, 1H), 5.59 (d, *J* = 8.8 Hz, 1H), and 3.24 (s, 3H) ppm; ^13^C NMR (125 MHz, CDCl3) δ 154.36 (quaternary, q, determined by DEPT-135), 151.13 (q), 151.05 (q), 148.16 (q), 133.51 (q), 133.05 (q), 130.76 (q), 129.47 (q), 129.40 (tertiary, t), 129.07 (t), 128.57 (q), 128.53 (t), 127.60 (t), 127.34 (t), 125.99 (t), 125.62 (t), 125.48 (t), 125.00 (t), 123.39 (t), 123.24 (t), 122.01 (t), 120.46 (t), 118.65 (q), 113.78 (t), and 56.55 (methyl) ppm; HR-MALDI-TOF mass (m/z) Calcd for C_45_H_27_BN_6_O_2_ [M]^+^ 694.2295, found [M]^+^ 694.8883; UV (CH_2_Cl_2_) λmax (10^4^ ε dm^3^mol^-1^cm^-1^) 271 (4.27), 300 (4.37), and 564 (8.71) nm; emission (nm) 577 nm (excitation at 564nm, quantum yield: 13%).

**Figure SI-1** ^1^H NMR spectrum of **1b** in CDCl_3_.

**Figure SI-2** COSY spectrum of **1b** in CDCl_3_.

**Figure SI-3** NOESY spectrum of **1b** in CD_2_Cl_2_.

**Figure SI-4** APCI mass spectrum of **1b**.

**1c**: ^1^H NMR (500 MHz, CDCl_3_) δ 8.63 (broad s, 6H), 7.87 (d, *J* = 8.0 Hz, 1H), 7.83-7.80 (*AA’BB’*-spin system, 6H), 7.48 (t, *J* = 8.0 Hz, 1H), 7.45 (d, *J* = 8.6 Hz, 1H), 7.18 (t, *J* = 7.9 Hz, 1H), 7.12 (d, *J* = 7.2 Hz, 1H), 7.03 (d, *J* = 8.8 Hz, 1H), 6.94 (dt, *J* = 6.8 and 1.3 Hz, 1H), 6.83 (dd, *J* = 7.4 and 1.1 Hz, 1H), 6.79 (d, *J* = 8.7 Hz, 1H), 6.69 (d, *J* = 8.5 Hz, 1H), and 5.72 (d, *J* = 8.8 Hz, 1H) ppm; 151.74 (q), 149.23 (q), 149.20 (q), 142.04 (q), 130.12 (q), 129.92 (q), 129.62 (q), 129.02 (t), 128.35 (t), 128.00 (q), 126.97 (q), 126.53 (t), 125.98 (t), 125.23 (t), 124.48 (q), 124.26 (t), 124.03 (t), 123.25 (t), 123.18 (t), 120.96 (t), 118.39 (t), 115.07 (t), 114.26 (q), 111.83 (q), and 106.53 (t) ppm; HR-MALDI-TOF mass (m/z) Calcd for C_44_H_23_BN_6_O_2_ [M]^+^ 678.1982, found [M]^+^678.9257; UV (CH_2_Cl_2_) λmax (10^4^ ε dm^3^mol^-1^cm^-1^) 263 (6.90), 304 (5.67), and 566 (9.04) nm; emission (nm) 578 nm (excitation at 566nm, quantum yield: 4%).

**Figure SI-5** ^1^H NMR spectrum of **1c** in CDCl_3_.

**Figure SI-6** COSY spectrum of **1c** in CDCl_3_.

**Figure SI-7** APCI mass spectrum of **1c**.

§2 Electrochemistry

Cyclic voltammetry (CV) and differential pulse voltammetry (DPV) were recorded using a BAS instrument with a glassy carbon working electrode, an Ag/AgCl reference electrode, and a platinum counter electrode. Distilled CH₂Cl₂ was used as the solvent, and recrystallized tetrabutylammonium PF₆ was used as the supporting electrolyte. The potentials were calibrated against the ferrocene/ferrocenium redox couple and referenced to 0 V.

| Potential (vs. Fc/Fc+) / V | CV |
| --- | --- |
| -0.741 | 3.048e-08 |
| -0.742 | 1.133e-07 |
| -0.743 | 1.442e-07 |
| -0.744 | 1.541e-07 |
| -0.745 | 1.651e-07 |
| -0.746 | 1.711e-07 |
| -0.747 | 1.807e-07 |
| -0.748 | 1.832e-07 |
| -0.749 | 1.899e-07 |
| -0.75 | 1.904e-07 |
| -0.751 | 1.954e-07 |
| -0.752 | 1.985e-07 |
| -0.753 | 2.03e-07 |
| -0.754 | 2.032e-07 |
| -0.755 | 2.102e-07 |
| -0.756 | 2.101e-07 |
| -0.757 | 2.147e-07 |
| -0.758 | 2.167e-07 |
| -0.759 | 2.212e-07 |
| -0.76 | 2.188e-07 |
| -0.761 | 2.24e-07 |
| -0.762 | 2.257e-07 |
| -0.763 | 2.295e-07 |
| -0.764 | 2.296e-07 |
| -0.765 | 2.362e-07 |
| -0.766 | 2.35e-07 |
| -0.767 | 2.392e-07 |
| -0.768 | 2.404e-07 |
| -0.769 | 2.436e-07 |
| -0.77 | 2.438e-07 |
| -0.771 | 2.482e-07 |
| -0.772 | 2.474e-07 |
| -0.773 | 2.524e-07 |
| -0.774 | 2.523e-07 |
| -0.775 | 2.568e-07 |
| -0.776 | 2.548e-07 |
| -0.777 | 2.589e-07 |
| -0.778 | 2.609e-07 |
| -0.779 | 2.64e-07 |
| -0.78 | 2.633e-07 |
| -0.781 | 2.696e-07 |
| -0.782 | 2.673e-07 |
| -0.783 | 2.703e-07 |
| -0.784 | 2.719e-07 |
| -0.785 | 2.743e-07 |
| -0.786 | 2.716e-07 |
| -0.787 | 2.77e-07 |
| -0.788 | 2.765e-07 |
| -0.789 | 2.803e-07 |
| -0.79 | 2.813e-07 |
| -0.791 | 2.852e-07 |
| -0.792 | 2.845e-07 |
| -0.793 | 2.871e-07 |
| -0.794 | 2.875e-07 |
| -0.795 | 2.905e-07 |
| -0.796 | 2.894e-07 |
| -0.797 | 2.952e-07 |
| -0.798 | 2.898e-07 |
| -0.799 | 2.922e-07 |
| -0.8 | 2.957e-07 |
| -0.801 | 2.994e-07 |
| -0.802 | 2.982e-07 |
| -0.803 | 3.045e-07 |
| -0.804 | 3.026e-07 |
| -0.805 | 3.071e-07 |
| -0.806 | 3.066e-07 |
| -0.807 | 3.106e-07 |
| -0.808 | 3.1e-07 |
| -0.809 | 3.136e-07 |
| -0.81 | 3.143e-07 |
| -0.811 | 3.153e-07 |
| -0.812 | 3.142e-07 |
| -0.813 | 3.198e-07 |
| -0.814 | 3.178e-07 |
| -0.815 | 3.2e-07 |
| -0.816 | 3.213e-07 |
| -0.817 | 3.235e-07 |
| -0.818 | 3.23e-07 |
| -0.819 | 3.28e-07 |
| -0.82 | 3.258e-07 |
| -0.821 | 3.293e-07 |
| -0.822 | 3.288e-07 |
| -0.823 | 3.322e-07 |
| -0.824 | 3.323e-07 |
| -0.825 | 3.36e-07 |
| -0.826 | 3.356e-07 |
| -0.827 | 3.379e-07 |
| -0.828 | 3.372e-07 |
| -0.829 | 3.427e-07 |
| -0.83 | 3.398e-07 |
| -0.831 | 3.435e-07 |
| -0.832 | 3.44e-07 |
| -0.833 | 3.464e-07 |
| -0.834 | 3.443e-07 |
| -0.835 | 3.496e-07 |
| -0.836 | 3.478e-07 |
| -0.837 | 3.495e-07 |
| -0.838 | 3.5e-07 |
| -0.839 | 3.544e-07 |
| -0.84 | 3.527e-07 |
| -0.841 | 3.567e-07 |
| -0.842 | 3.567e-07 |
| -0.843 | 3.608e-07 |
| -0.844 | 3.595e-07 |
| -0.845 | 3.639e-07 |
| -0.846 | 3.617e-07 |
| -0.847 | 3.655e-07 |
| -0.848 | 3.654e-07 |
| -0.849 | 3.684e-07 |
| -0.85 | 3.701e-07 |
| -0.851 | 3.718e-07 |
| -0.852 | 3.702e-07 |
| -0.853 | 3.723e-07 |
| -0.854 | 3.729e-07 |
| -0.855 | 3.759e-07 |
| -0.856 | 3.744e-07 |
| -0.857 | 3.779e-07 |
| -0.858 | 3.772e-07 |
| -0.859 | 3.8e-07 |
| -0.86 | 3.779e-07 |
| -0.861 | 3.842e-07 |
| -0.862 | 3.81e-07 |
| -0.863 | 3.843e-07 |
| -0.864 | 3.855e-07 |
| -0.865 | 3.872e-07 |
| -0.866 | 3.863e-07 |
| -0.867 | 3.917e-07 |
| -0.868 | 3.894e-07 |
| -0.869 | 3.938e-07 |
| -0.87 | 3.917e-07 |
| -0.871 | 3.961e-07 |
| -0.872 | 3.939e-07 |
| -0.873 | 3.983e-07 |
| -0.874 | 3.99e-07 |
| -0.875 | 4.024e-07 |
| -0.876 | 3.989e-07 |
| -0.877 | 4.046e-07 |
| -0.878 | 4.017e-07 |
| -0.879 | 4.055e-07 |
| -0.88 | 4.058e-07 |
| -0.881 | 4.086e-07 |
| -0.882 | 4.075e-07 |
| -0.883 | 4.118e-07 |
| -0.884 | 4.106e-07 |
| -0.885 | 4.149e-07 |
| -0.886 | 4.135e-07 |
| -0.887 | 4.189e-07 |
| -0.888 | 4.137e-07 |
| -0.889 | 4.2e-07 |
| -0.89 | 4.191e-07 |
| -0.891 | 4.219e-07 |
| -0.892 | 4.202e-07 |
| -0.893 | 4.266e-07 |
| -0.894 | 4.237e-07 |
| -0.895 | 4.268e-07 |
| -0.896 | 4.269e-07 |
| -0.897 | 4.306e-07 |
| -0.898 | 4.271e-07 |
| -0.899 | 4.331e-07 |
| -0.9 | 4.301e-07 |
| -0.901 | 4.392e-07 |
| -0.902 | 4.353e-07 |
| -0.903 | 4.378e-07 |
| -0.904 | 4.351e-07 |
| -0.905 | 4.385e-07 |
| -0.906 | 4.388e-07 |
| -0.907 | 4.414e-07 |
| -0.908 | 4.39e-07 |
| -0.909 | 4.44e-07 |
| -0.91 | 4.414e-07 |
| -0.911 | 4.444e-07 |
| -0.912 | 4.442e-07 |
| -0.913 | 4.458e-07 |
| -0.914 | 4.433e-07 |
| -0.915 | 4.497e-07 |
| -0.916 | 4.48e-07 |
| -0.917 | 4.525e-07 |
| -0.918 | 4.501e-07 |
| -0.919 | 4.548e-07 |
| -0.92 | 4.546e-07 |
| -0.921 | 4.583e-07 |
| -0.922 | 4.562e-07 |
| -0.923 | 4.595e-07 |
| -0.924 | 4.567e-07 |
| -0.925 | 4.636e-07 |
| -0.926 | 4.615e-07 |
| -0.927 | 4.656e-07 |
| -0.928 | 4.639e-07 |
| -0.929 | 4.677e-07 |
| -0.93 | 4.647e-07 |
| -0.931 | 4.707e-07 |
| -0.932 | 4.666e-07 |
| -0.933 | 4.72e-07 |
| -0.934 | 4.692e-07 |
| -0.935 | 4.742e-07 |
| -0.936 | 4.712e-07 |
| -0.937 | 4.767e-07 |
| -0.938 | 4.74e-07 |
| -0.939 | 4.765e-07 |
| -0.94 | 4.739e-07 |

| Potential (vs. Fc/Fc+) / V | Current / A |
| --- | --- |
| -0.728 | -4.93e-08 |
| -0.727 | -1.287e-07 |
| -0.726 | -1.594e-07 |
| -0.725 | -1.683e-07 |
| -0.724 | -1.788e-07 |
| -0.723 | -1.864e-07 |
| -0.722 | -1.923e-07 |
| -0.721 | -1.956e-07 |
| -0.72 | -2.026e-07 |
| -0.719 | -2.039e-07 |
| -0.718 | -2.091e-07 |
| -0.717 | -2.112e-07 |
| -0.716 | -2.163e-07 |
| -0.715 | -2.175e-07 |
| -0.714 | -2.219e-07 |
| -0.713 | -2.236e-07 |
| -0.712 | -2.276e-07 |
| -0.711 | -2.274e-07 |
| -0.71 | -2.347e-07 |
| -0.709 | -2.324e-07 |
| -0.708 | -2.361e-07 |
| -0.707 | -2.397e-07 |
| -0.706 | -2.428e-07 |
| -0.705 | -2.418e-07 |
| -0.704 | -2.482e-07 |
| -0.703 | -2.48e-07 |
| -0.702 | -2.534e-07 |
| -0.701 | -2.531e-07 |
| -0.7 | -2.563e-07 |
| -0.699 | -2.559e-07 |
| -0.698 | -2.606e-07 |
| -0.697 | -2.615e-07 |
| -0.696 | -2.68e-07 |
| -0.695 | -2.656e-07 |
| -0.694 | -2.69e-07 |
| -0.693 | -2.676e-07 |
| -0.692 | -2.71e-07 |
| -0.691 | -2.723e-07 |
| -0.69 | -2.75e-07 |
| -0.689 | -2.75e-07 |
| -0.688 | -2.8e-07 |
| -0.687 | -2.79e-07 |
| -0.686 | -2.829e-07 |
| -0.685 | -2.831e-07 |
| -0.684 | -2.867e-07 |
| -0.683 | -2.831e-07 |
| -0.682 | -2.895e-07 |
| -0.681 | -2.894e-07 |
| -0.68 | -2.921e-07 |
| -0.679 | -2.927e-07 |
| -0.678 | -2.98e-07 |
| -0.677 | -2.96e-07 |
| -0.676 | -2.997e-07 |
| -0.675 | -3.006e-07 |
| -0.674 | -3.032e-07 |
| -0.673 | -3.014e-07 |
| -0.672 | -3.08e-07 |
| -0.671 | -3.066e-07 |
| -0.67 | -3.116e-07 |
| -0.669 | -3.11e-07 |
| -0.668 | -3.13e-07 |
| -0.667 | -3.122e-07 |
| -0.666 | -3.164e-07 |
| -0.665 | -3.159e-07 |
| -0.664 | -3.191e-07 |
| -0.663 | -3.183e-07 |
| -0.662 | -3.24e-07 |
| -0.661 | -3.218e-07 |
| -0.66 | -3.254e-07 |
| -0.659 | -3.258e-07 |
| -0.658 | -3.282e-07 |
| -0.657 | -3.257e-07 |
| -0.656 | -3.326e-07 |
| -0.655 | -3.314e-07 |
| -0.654 | -3.352e-07 |
| -0.653 | -3.353e-07 |
| -0.652 | -3.393e-07 |
| -0.651 | -3.385e-07 |
| -0.65 | -3.416e-07 |
| -0.649 | -3.404e-07 |
| -0.648 | -3.447e-07 |
| -0.647 | -3.431e-07 |
| -0.646 | -3.493e-07 |
| -0.645 | -3.496e-07 |
| -0.644 | -3.532e-07 |
| -0.643 | -3.538e-07 |
| -0.642 | -3.556e-07 |
| -0.641 | -3.535e-07 |
| -0.64 | -3.587e-07 |
| -0.639 | -3.573e-07 |
| -0.638 | -3.611e-07 |
| -0.637 | -3.605e-07 |
| -0.636 | -3.635e-07 |
| -0.635 | -3.626e-07 |
| -0.634 | -3.664e-07 |
| -0.633 | -3.666e-07 |
| -0.632 | -3.672e-07 |
| -0.631 | -3.671e-07 |
| -0.63 | -3.739e-07 |
| -0.629 | -3.711e-07 |
| -0.628 | -3.756e-07 |
| -0.627 | -3.753e-07 |
| -0.626 | -3.796e-07 |
| -0.625 | -3.779e-07 |
| -0.624 | -3.832e-07 |
| -0.623 | -3.809e-07 |
| -0.622 | -3.845e-07 |
| -0.621 | -3.84e-07 |
| -0.62 | -3.887e-07 |
| -0.619 | -3.882e-07 |
| -0.618 | -3.916e-07 |
| -0.617 | -3.91e-07 |
| -0.616 | -3.937e-07 |
| -0.615 | -3.929e-07 |
| -0.614 | -3.974e-07 |
| -0.613 | -3.956e-07 |
| -0.612 | -3.996e-07 |
| -0.611 | -4.008e-07 |
| -0.61 | -4.032e-07 |
| -0.609 | -4.01e-07 |
| -0.608 | -4.063e-07 |
| -0.607 | -4.05e-07 |
| -0.606 | -4.066e-07 |
| -0.605 | -4.078e-07 |
| -0.604 | -4.116e-07 |
| -0.603 | -4.103e-07 |
| -0.602 | -4.136e-07 |
| -0.601 | -4.133e-07 |
| -0.6 | -4.169e-07 |
| -0.599 | -4.151e-07 |
| -0.598 | -4.199e-07 |
| -0.597 | -4.175e-07 |
| -0.596 | -4.216e-07 |
| -0.595 | -4.228e-07 |
| -0.594 | -4.261e-07 |
| -0.593 | -4.269e-07 |
| -0.592 | -4.305e-07 |
| -0.591 | -4.277e-07 |
| -0.59 | -4.315e-07 |
| -0.589 | -4.3e-07 |
| -0.588 | -4.343e-07 |
| -0.587 | -4.333e-07 |
| -0.586 | -4.372e-07 |
| -0.585 | -4.365e-07 |
| -0.584 | -4.389e-07 |
| -0.583 | -4.364e-07 |
| -0.582 | -4.437e-07 |
| -0.581 | -4.388e-07 |
| -0.58 | -4.432e-07 |
| -0.579 | -4.441e-07 |
| -0.578 | -4.464e-07 |
| -0.577 | -4.45e-07 |
| -0.576 | -4.519e-07 |
| -0.575 | -4.487e-07 |
| -0.574 | -4.528e-07 |
| -0.573 | -4.519e-07 |
| -0.572 | -4.562e-07 |
| -0.571 | -4.537e-07 |
| -0.57 | -4.578e-07 |
| -0.569 | -4.569e-07 |
| -0.568 | -4.602e-07 |
| -0.567 | -4.586e-07 |
| -0.566 | -4.62e-07 |
| -0.565 | -4.599e-07 |
| -0.564 | -4.632e-07 |
| -0.563 | -4.627e-07 |
| -0.562 | -4.655e-07 |
| -0.561 | -4.635e-07 |
| -0.56 | -4.691e-07 |
| -0.559 | -4.654e-07 |
| -0.558 | -4.689e-07 |
| -0.557 | -4.678e-07 |
| -0.556 | -4.734e-07 |
| -0.555 | -4.686e-07 |
| -0.554 | -4.747e-07 |
| -0.553 | -4.727e-07 |
| -0.552 | -4.779e-07 |
| -0.551 | -4.748e-07 |
| -0.55 | -4.821e-07 |
| -0.549 | -4.789e-07 |
| -0.548 | -4.834e-07 |
| -0.547 | -4.826e-07 |
| -0.546 | -4.857e-07 |
| -0.545 | -4.837e-07 |
| -0.544 | -4.89e-07 |
| -0.543 | -4.852e-07 |
| -0.542 | -4.937e-07 |
| -0.541 | -4.898e-07 |
| -0.54 | -4.933e-07 |
| -0.539 | -4.898e-07 |
| -0.538 | -4.953e-07 |
| -0.537 | -4.931e-07 |
| -0.536 | -4.975e-07 |
| -0.535 | -4.95e-07 |
| -0.534 | -5.003e-07 |
| -0.533 | -4.96e-07 |
| -0.532 | -5.004e-07 |
| -0.531 | -5e-07 |
| -0.53 | -5.024e-07 |
| -0.529 | -4.99e-07 |

| Potential (vs. Fc/Fc+) / V | DPV |
| --- | --- |
| -0.18 | -4.346e-07 |
| -0.176 | -4.343e-07 |
| -0.172 | -4.362e-07 |
| -0.168 | -4.388e-07 |
| -0.164 | -4.415e-07 |
| -0.16 | -4.445e-07 |
| -0.156 | -4.503e-07 |
| -0.152 | -4.546e-07 |
| -0.148 | -4.604e-07 |
| -0.144 | -4.665e-07 |
| -0.14 | -4.734e-07 |
| -0.136 | -4.823e-07 |
| -0.132 | -4.913e-07 |
| -0.128 | -5e-07 |
| -0.124 | -5.128e-07 |
| -0.12 | -5.251e-07 |
| -0.116 | -5.383e-07 |
| -0.112 | -5.549e-07 |
| -0.108 | -5.712e-07 |
| -0.104 | -5.908e-07 |
| -0.1 | -6.111e-07 |
| -0.096 | -6.349e-07 |
| -0.092 | -6.603e-07 |
| -0.088 | -6.876e-07 |
| -0.084 | -7.19e-07 |
| -0.08 | -7.53e-07 |
| -0.076 | -7.871e-07 |
| -0.072 | -8.251e-07 |
| -0.068 | -8.661e-07 |
| -0.064 | -9.082e-07 |
| -0.06 | -9.512e-07 |
| -0.056 | -9.969e-07 |
| -0.052 | -1.043e-06 |
| -0.048 | -1.087e-06 |
| -0.044 | -1.131e-06 |
| -0.04 | -1.173e-06 |
| -0.036 | -1.212e-06 |
| -0.032 | -1.244e-06 |
| -0.028 | -1.272e-06 |
| -0.024 | -1.292e-06 |
| -0.02 | -1.308e-06 |
| -0.016 | -1.315e-06 |
| -0.012 | -1.315e-06 |
| -0.008 | -1.308e-06 |
| -0.004 | -1.293e-06 |
| 0 | -1.272e-06 |
| 0.004 | -1.241e-06 |
| 0.008 | -1.208e-06 |
| 0.012 | -1.169e-06 |
| 0.016 | -1.129e-06 |
| 0.02 | -1.087e-06 |
| 0.024 | -1.045e-06 |
| 0.028 | -1.001e-06 |
| 0.032 | -9.584e-07 |
| 0.036 | -9.173e-07 |
| 0.04 | -8.786e-07 |
| 0.044 | -8.419e-07 |
| 0.048 | -8.079e-07 |
| 0.052 | -7.761e-07 |
| 0.056 | -7.481e-07 |
| 0.06 | -7.239e-07 |
| 0.064 | -6.995e-07 |
| 0.068 | -6.797e-07 |
| 0.072 | -6.607e-07 |
| 0.076 | -6.462e-07 |
| 0.08 | -6.322e-07 |
| 0.084 | -6.199e-07 |
| 0.088 | -6.09e-07 |
| 0.092 | -6e-07 |
| 0.096 | -5.931e-07 |
| 0.1 | -5.866e-07 |
| 0.104 | -5.816e-07 |
| 0.108 | -5.79e-07 |
| 0.112 | -5.748e-07 |
| 0.116 | -5.738e-07 |
| 0.12 | -5.726e-07 |
| 0.124 | -5.729e-07 |
| 0.128 | -5.731e-07 |
| 0.132 | -5.745e-07 |
| 0.136 | -5.774e-07 |
| 0.14 | -5.81e-07 |
| 0.144 | -5.83e-07 |
| 0.148 | -5.865e-07 |
| 0.152 | -5.898e-07 |
| 0.156 | -5.941e-07 |
| 0.16 | -5.977e-07 |
| 0.164 | -5.998e-07 |
| 0.168 | -6.045e-07 |
| 0.172 | -6.095e-07 |
| 0.176 | -6.125e-07 |
| 0.18 | -6.132e-07 |
| 0.184 | -6.162e-07 |
| 0.188 | -6.18e-07 |
| 0.192 | -6.208e-07 |
| 0.196 | -6.215e-07 |
| 0.2 | -6.239e-07 |
| 0.204 | -6.257e-07 |
| 0.208 | -6.289e-07 |
| 0.212 | -6.301e-07 |
| 0.216 | -6.36e-07 |
| 0.22 | -6.392e-07 |
| 0.224 | -6.435e-07 |
| 0.228 | -6.501e-07 |
| 0.232 | -6.576e-07 |
| 0.236 | -6.669e-07 |
| 0.24 | -6.764e-07 |
| 0.244 | -6.882e-07 |
| 0.248 | -7.034e-07 |
| 0.252 | -7.189e-07 |
| 0.256 | -7.378e-07 |
| 0.26 | -7.577e-07 |
| 0.264 | -7.848e-07 |
| 0.268 | -8.13e-07 |
| 0.272 | -8.447e-07 |
| 0.276 | -8.806e-07 |
| 0.28 | -9.252e-07 |
| 0.284 | -9.733e-07 |
| 0.288 | -1.03e-06 |
| 0.292 | -1.092e-06 |
| 0.296 | -1.167e-06 |
| 0.3 | -1.252e-06 |
| 0.304 | -1.349e-06 |
| 0.308 | -1.459e-06 |
| 0.312 | -1.591e-06 |
| 0.316 | -1.745e-06 |
| 0.32 | -1.923e-06 |
| 0.324 | -2.13e-06 |
| 0.328 | -2.376e-06 |
| 0.332 | -2.66e-06 |
| 0.336 | -2.983e-06 |
| 0.34 | -3.356e-06 |
| 0.344 | -3.783e-06 |
| 0.348 | -4.266e-06 |
| 0.352 | -4.813e-06 |
| 0.356 | -5.43e-06 |
| 0.36 | -6.117e-06 |
| 0.364 | -6.879e-06 |
| 0.368 | -7.708e-06 |
| 0.372 | -8.62e-06 |
| 0.376 | -9.61e-06 |
| 0.38 | -1.066e-05 |
| 0.384 | -1.176e-05 |
| 0.388 | -1.295e-05 |
| 0.392 | -1.42e-05 |
| 0.396 | -1.548e-05 |
| 0.4 | -1.68e-05 |
| 0.404 | -1.814e-05 |
| 0.408 | -1.949e-05 |
| 0.412 | -2.083e-05 |
| 0.416 | -2.214e-05 |
| 0.42 | -2.342e-05 |
| 0.424 | -2.466e-05 |
| 0.428 | -2.58e-05 |
| 0.432 | -2.689e-05 |
| 0.436 | -2.785e-05 |
| 0.44 | -2.873e-05 |
| 0.444 | -2.946e-05 |
| 0.448 | -3.003e-05 |
| 0.452 | -3.045e-05 |
| 0.456 | -3.069e-05 |
| 0.46 | -3.074e-05 |
| 0.464 | -3.056e-05 |
| 0.468 | -3.017e-05 |
| 0.472 | -2.957e-05 |
| 0.476 | -2.874e-05 |
| 0.48 | -2.768e-05 |
| 0.484 | -2.642e-05 |
| 0.488 | -2.499e-05 |
| 0.492 | -2.342e-05 |
| 0.496 | -2.173e-05 |
| 0.5 | -2e-05 |
| 0.504 | -1.826e-05 |
| 0.508 | -1.653e-05 |
| 0.512 | -1.488e-05 |
| 0.516 | -1.334e-05 |
| 0.52 | -1.193e-05 |
| 0.524 | -1.064e-05 |
| 0.528 | -9.475e-06 |
| 0.532 | -8.468e-06 |
| 0.536 | -7.589e-06 |
| 0.54 | -6.852e-06 |
| 0.544 | -6.218e-06 |
| 0.548 | -5.692e-06 |
| 0.552 | -5.25e-06 |
| 0.556 | -4.879e-06 |
| 0.56 | -4.567e-06 |
| 0.564 | -4.32e-06 |
| 0.568 | -4.126e-06 |
| 0.572 | -3.982e-06 |
| 0.576 | -3.887e-06 |
| 0.58 | -3.849e-06 |
| 0.584 | -3.866e-06 |
| 0.588 | -3.938e-06 |
| 0.592 | -4.05e-06 |
| 0.596 | -4.222e-06 |
| 0.6 | -4.45e-06 |
| 0.604 | -4.726e-06 |
| 0.608 | -5.067e-06 |
| 0.612 | -5.465e-06 |
| 0.616 | -5.927e-06 |

| Potential (vs. Fc/Fc+) / V | Current / A |
| --- | --- |
| -0.188 | 4.246e-07 |
| -0.192 | 4.172e-07 |
| -0.196 | 4.096e-07 |
| -0.2 | 4.038e-07 |
| -0.204 | 3.988e-07 |
| -0.208 | 3.945e-07 |
| -0.212 | 3.888e-07 |
| -0.216 | 3.846e-07 |
| -0.22 | 3.814e-07 |
| -0.224 | 3.777e-07 |
| -0.228 | 3.756e-07 |
| -0.232 | 3.717e-07 |
| -0.236 | 3.697e-07 |
| -0.24 | 3.667e-07 |
| -0.244 | 3.644e-07 |
| -0.248 | 3.623e-07 |
| -0.252 | 3.6e-07 |
| -0.256 | 3.58e-07 |
| -0.26 | 3.547e-07 |
| -0.264 | 3.532e-07 |
| -0.268 | 3.512e-07 |
| -0.272 | 3.496e-07 |
| -0.276 | 3.483e-07 |
| -0.28 | 3.458e-07 |
| -0.284 | 3.44e-07 |
| -0.288 | 3.424e-07 |
| -0.292 | 3.405e-07 |
| -0.296 | 3.39e-07 |
| -0.3 | 3.376e-07 |
| -0.304 | 3.357e-07 |
| -0.308 | 3.353e-07 |
| -0.312 | 3.336e-07 |
| -0.316 | 3.321e-07 |
| -0.32 | 3.308e-07 |
| -0.324 | 3.296e-07 |
| -0.328 | 3.282e-07 |
| -0.332 | 3.27e-07 |
| -0.336 | 3.255e-07 |
| -0.34 | 3.233e-07 |
| -0.344 | 3.224e-07 |
| -0.348 | 3.215e-07 |
| -0.352 | 3.207e-07 |
| -0.356 | 3.2e-07 |
| -0.36 | 3.191e-07 |
| -0.364 | 3.184e-07 |
| -0.368 | 3.181e-07 |
| -0.372 | 3.168e-07 |
| -0.376 | 3.166e-07 |
| -0.38 | 3.157e-07 |
| -0.384 | 3.153e-07 |
| -0.388 | 3.149e-07 |
| -0.392 | 3.149e-07 |
| -0.396 | 3.146e-07 |
| -0.4 | 3.146e-07 |
| -0.404 | 3.15e-07 |
| -0.408 | 3.144e-07 |
| -0.412 | 3.158e-07 |
| -0.416 | 3.142e-07 |
| -0.42 | 3.149e-07 |
| -0.424 | 3.152e-07 |
| -0.428 | 3.154e-07 |
| -0.432 | 3.152e-07 |
| -0.436 | 3.161e-07 |
| -0.44 | 3.16e-07 |
| -0.444 | 3.169e-07 |
| -0.448 | 3.175e-07 |
| -0.452 | 3.177e-07 |
| -0.456 | 3.183e-07 |
| -0.46 | 3.193e-07 |
| -0.464 | 3.191e-07 |
| -0.468 | 3.197e-07 |
| -0.472 | 3.213e-07 |
| -0.476 | 3.222e-07 |
| -0.48 | 3.225e-07 |
| -0.484 | 3.23e-07 |
| -0.488 | 3.247e-07 |
| -0.492 | 3.243e-07 |
| -0.496 | 3.253e-07 |
| -0.5 | 3.256e-07 |
| -0.504 | 3.264e-07 |
| -0.508 | 3.273e-07 |
| -0.512 | 3.282e-07 |
| -0.516 | 3.274e-07 |
| -0.52 | 3.276e-07 |
| -0.524 | 3.289e-07 |
| -0.528 | 3.284e-07 |
| -0.532 | 3.288e-07 |
| -0.536 | 3.291e-07 |
| -0.54 | 3.29e-07 |
| -0.544 | 3.301e-07 |
| -0.548 | 3.301e-07 |
| -0.552 | 3.305e-07 |
| -0.556 | 3.324e-07 |
| -0.56 | 3.319e-07 |
| -0.564 | 3.318e-07 |
| -0.568 | 3.327e-07 |
| -0.572 | 3.341e-07 |
| -0.576 | 3.353e-07 |
| -0.58 | 3.355e-07 |
| -0.584 | 3.368e-07 |
| -0.588 | 3.384e-07 |
| -0.592 | 3.391e-07 |
| -0.596 | 3.414e-07 |
| -0.6 | 3.422e-07 |
| -0.604 | 3.445e-07 |
| -0.608 | 3.457e-07 |
| -0.612 | 3.468e-07 |
| -0.616 | 3.488e-07 |
| -0.62 | 3.498e-07 |
| -0.624 | 3.516e-07 |
| -0.628 | 3.526e-07 |
| -0.632 | 3.549e-07 |
| -0.636 | 3.562e-07 |
| -0.64 | 3.586e-07 |
| -0.644 | 3.604e-07 |
| -0.648 | 3.622e-07 |
| -0.652 | 3.637e-07 |
| -0.656 | 3.655e-07 |
| -0.66 | 3.679e-07 |
| -0.664 | 3.7e-07 |
| -0.668 | 3.72e-07 |
| -0.672 | 3.718e-07 |
| -0.676 | 3.743e-07 |
| -0.68 | 3.756e-07 |
| -0.684 | 3.78e-07 |
| -0.688 | 3.787e-07 |
| -0.692 | 3.805e-07 |
| -0.696 | 3.827e-07 |
| -0.7 | 3.839e-07 |
| -0.704 | 3.857e-07 |
| -0.708 | 3.877e-07 |
| -0.712 | 3.886e-07 |
| -0.716 | 3.902e-07 |
| -0.72 | 3.921e-07 |
| -0.724 | 3.933e-07 |
| -0.728 | 3.956e-07 |
| -0.732 | 3.972e-07 |
| -0.736 | 3.98e-07 |
| -0.74 | 4e-07 |
| -0.744 | 4.015e-07 |
| -0.748 | 4.029e-07 |
| -0.752 | 4.032e-07 |
| -0.756 | 4.042e-07 |
| -0.76 | 4.058e-07 |
| -0.764 | 4.07e-07 |
| -0.768 | 4.08e-07 |
| -0.772 | 4.093e-07 |
| -0.776 | 4.111e-07 |
| -0.78 | 4.122e-07 |
| -0.784 | 4.131e-07 |
| -0.788 | 4.138e-07 |
| -0.792 | 4.142e-07 |
| -0.796 | 4.146e-07 |
| -0.8 | 4.152e-07 |
| -0.804 | 4.165e-07 |
| -0.808 | 4.169e-07 |
| -0.812 | 4.18e-07 |
| -0.816 | 4.184e-07 |
| -0.82 | 4.185e-07 |
| -0.824 | 4.178e-07 |
| -0.828 | 4.191e-07 |
| -0.832 | 4.199e-07 |
| -0.836 | 4.205e-07 |
| -0.84 | 4.201e-07 |
| -0.844 | 4.211e-07 |
| -0.848 | 4.207e-07 |
| -0.852 | 4.224e-07 |
| -0.856 | 4.225e-07 |
| -0.86 | 4.223e-07 |
| -0.864 | 4.242e-07 |
| -0.868 | 4.249e-07 |
| -0.872 | 4.262e-07 |
| -0.876 | 4.265e-07 |
| -0.88 | 4.275e-07 |
| -0.884 | 4.282e-07 |
| -0.888 | 4.298e-07 |
| -0.892 | 4.308e-07 |
| -0.896 | 4.306e-07 |
| -0.9 | 4.328e-07 |
| -0.904 | 4.334e-07 |
| -0.908 | 4.338e-07 |
| -0.912 | 4.347e-07 |
| -0.916 | 4.363e-07 |
| -0.92 | 4.387e-07 |
| -0.924 | 4.399e-07 |
| -0.928 | 4.404e-07 |
| -0.932 | 4.422e-07 |
| -0.936 | 4.435e-07 |
| -0.94 | 4.447e-07 |
| -0.944 | 4.463e-07 |
| -0.948 | 4.493e-07 |
| -0.952 | 4.502e-07 |
| -0.956 | 4.536e-07 |
| -0.96 | 4.557e-07 |
| -0.964 | 4.578e-07 |
| -0.968 | 4.601e-07 |
| -0.972 | 4.634e-07 |
| -0.976 | 4.661e-07 |
| -0.98 | 4.682e-07 |
| -0.984 | 4.694e-07 |

**Figure SI-8** Cyclic and differential pulse voltammograms of **1b**.

| Potential (vs. Fc/Fc+) / V | CV |
| --- | --- |
| -0.598 | 7.001e-08 |
| -0.599 | 1.579e-07 |
| -0.6 | 1.885e-07 |
| -0.601 | 2e-07 |
| -0.602 | 2.108e-07 |
| -0.603 | 2.145e-07 |
| -0.604 | 2.211e-07 |
| -0.605 | 2.254e-07 |
| -0.606 | 2.298e-07 |
| -0.607 | 2.318e-07 |
| -0.608 | 2.398e-07 |
| -0.609 | 2.407e-07 |
| -0.61 | 2.449e-07 |
| -0.611 | 2.476e-07 |
| -0.612 | 2.522e-07 |
| -0.613 | 2.547e-07 |
| -0.614 | 2.587e-07 |
| -0.615 | 2.6e-07 |
| -0.616 | 2.642e-07 |
| -0.617 | 2.66e-07 |
| -0.618 | 2.697e-07 |
| -0.619 | 2.718e-07 |
| -0.62 | 2.734e-07 |
| -0.621 | 2.756e-07 |
| -0.622 | 2.776e-07 |
| -0.623 | 2.781e-07 |
| -0.624 | 2.838e-07 |
| -0.625 | 2.828e-07 |
| -0.626 | 2.869e-07 |
| -0.627 | 2.886e-07 |
| -0.628 | 2.906e-07 |
| -0.629 | 2.912e-07 |
| -0.63 | 2.945e-07 |
| -0.631 | 2.963e-07 |
| -0.632 | 2.967e-07 |
| -0.633 | 2.982e-07 |
| -0.634 | 3.034e-07 |
| -0.635 | 3.033e-07 |
| -0.636 | 3.071e-07 |
| -0.637 | 3.096e-07 |
| -0.638 | 3.115e-07 |
| -0.639 | 3.133e-07 |
| -0.64 | 3.163e-07 |
| -0.641 | 3.161e-07 |
| -0.642 | 3.175e-07 |
| -0.643 | 3.201e-07 |
| -0.644 | 3.211e-07 |
| -0.645 | 3.263e-07 |
| -0.646 | 3.26e-07 |
| -0.647 | 3.254e-07 |
| -0.648 | 3.275e-07 |
| -0.649 | 3.283e-07 |
| -0.65 | 3.332e-07 |
| -0.651 | 3.325e-07 |
| -0.652 | 3.35e-07 |
| -0.653 | 3.375e-07 |
| -0.654 | 3.388e-07 |
| -0.655 | 3.387e-07 |
| -0.656 | 3.424e-07 |
| -0.657 | 3.421e-07 |
| -0.658 | 3.424e-07 |
| -0.659 | 3.468e-07 |
| -0.66 | 3.473e-07 |
| -0.661 | 3.488e-07 |
| -0.662 | 3.516e-07 |
| -0.663 | 3.534e-07 |
| -0.664 | 3.542e-07 |
| -0.665 | 3.546e-07 |
| -0.666 | 3.592e-07 |
| -0.667 | 3.606e-07 |
| -0.668 | 3.611e-07 |
| -0.669 | 3.637e-07 |
| -0.67 | 3.661e-07 |
| -0.671 | 3.668e-07 |
| -0.672 | 3.682e-07 |
| -0.673 | 3.687e-07 |
| -0.674 | 3.697e-07 |
| -0.675 | 3.73e-07 |
| -0.676 | 3.736e-07 |
| -0.677 | 3.749e-07 |
| -0.678 | 3.773e-07 |
| -0.679 | 3.782e-07 |
| -0.68 | 3.796e-07 |
| -0.681 | 3.812e-07 |
| -0.682 | 3.835e-07 |
| -0.683 | 3.827e-07 |
| -0.684 | 3.846e-07 |
| -0.685 | 3.887e-07 |
| -0.686 | 3.889e-07 |
| -0.687 | 3.899e-07 |
| -0.688 | 3.932e-07 |
| -0.689 | 3.938e-07 |
| -0.69 | 3.954e-07 |
| -0.691 | 3.978e-07 |
| -0.692 | 3.982e-07 |
| -0.693 | 4.002e-07 |
| -0.694 | 4.015e-07 |
| -0.695 | 4.038e-07 |
| -0.696 | 4.101e-07 |
| -0.697 | 4.09e-07 |
| -0.698 | 4.103e-07 |
| -0.699 | 4.099e-07 |
| -0.7 | 4.104e-07 |
| -0.701 | 4.166e-07 |
| -0.702 | 4.154e-07 |
| -0.703 | 4.165e-07 |
| -0.704 | 4.196e-07 |
| -0.705 | 4.201e-07 |
| -0.706 | 4.209e-07 |
| -0.707 | 4.244e-07 |
| -0.708 | 4.251e-07 |
| -0.709 | 4.239e-07 |
| -0.71 | 4.276e-07 |
| -0.711 | 4.294e-07 |
| -0.712 | 4.297e-07 |
| -0.713 | 4.311e-07 |
| -0.714 | 4.342e-07 |
| -0.715 | 4.348e-07 |
| -0.716 | 4.35e-07 |
| -0.717 | 4.382e-07 |
| -0.718 | 4.378e-07 |
| -0.719 | 4.396e-07 |
| -0.72 | 4.415e-07 |
| -0.721 | 4.427e-07 |
| -0.722 | 4.429e-07 |
| -0.723 | 4.455e-07 |
| -0.724 | 4.452e-07 |
| -0.725 | 4.464e-07 |
| -0.726 | 4.477e-07 |
| -0.727 | 4.511e-07 |
| -0.728 | 4.514e-07 |
| -0.729 | 4.532e-07 |
| -0.73 | 4.56e-07 |
| -0.731 | 4.552e-07 |
| -0.732 | 4.557e-07 |
| -0.733 | 4.6e-07 |
| -0.734 | 4.596e-07 |
| -0.735 | 4.593e-07 |
| -0.736 | 4.633e-07 |
| -0.737 | 4.638e-07 |
| -0.738 | 4.645e-07 |
| -0.739 | 4.676e-07 |
| -0.74 | 4.674e-07 |
| -0.741 | 4.7e-07 |
| -0.742 | 4.707e-07 |
| -0.743 | 4.735e-07 |
| -0.744 | 4.72e-07 |
| -0.745 | 4.742e-07 |
| -0.746 | 4.769e-07 |
| -0.747 | 4.818e-07 |
| -0.748 | 4.8e-07 |
| -0.749 | 4.834e-07 |
| -0.75 | 4.809e-07 |
| -0.751 | 4.822e-07 |
| -0.752 | 4.85e-07 |
| -0.753 | 4.871e-07 |
| -0.754 | 4.865e-07 |
| -0.755 | 4.907e-07 |
| -0.756 | 4.898e-07 |
| -0.757 | 4.919e-07 |
| -0.758 | 4.924e-07 |
| -0.759 | 4.956e-07 |
| -0.76 | 4.911e-07 |
| -0.761 | 4.951e-07 |
| -0.762 | 4.98e-07 |
| -0.763 | 4.99e-07 |
| -0.764 | 4.983e-07 |
| -0.765 | 5.046e-07 |
| -0.766 | 5.025e-07 |
| -0.767 | 5.063e-07 |
| -0.768 | 5.069e-07 |
| -0.769 | 5.088e-07 |
| -0.77 | 5.077e-07 |
| -0.771 | 5.13e-07 |
| -0.772 | 5.108e-07 |
| -0.773 | 5.156e-07 |
| -0.774 | 5.143e-07 |
| -0.775 | 5.171e-07 |
| -0.776 | 5.165e-07 |
| -0.777 | 5.194e-07 |
| -0.778 | 5.209e-07 |
| -0.779 | 5.223e-07 |
| -0.78 | 5.216e-07 |
| -0.781 | 5.258e-07 |
| -0.782 | 5.234e-07 |
| -0.783 | 5.263e-07 |
| -0.784 | 5.285e-07 |
| -0.785 | 5.292e-07 |
| -0.786 | 5.266e-07 |
| -0.787 | 5.313e-07 |
| -0.788 | 5.308e-07 |
| -0.789 | 5.335e-07 |
| -0.79 | 5.339e-07 |
| -0.791 | 5.359e-07 |
| -0.792 | 5.367e-07 |
| -0.793 | 5.387e-07 |
| -0.794 | 5.389e-07 |
| -0.795 | 5.411e-07 |
| -0.796 | 5.388e-07 |
| -0.797 | 5.443e-07 |

| Potential (vs. Fc/Fc+) / V | Current / A |
| --- | --- |
| -0.002 | -1.871e-07 |
| -0.001 | -4.608e-07 |
| 0 | -5.627e-07 |
| 0.001 | -6.009e-07 |
| 0.002 | -6.23e-07 |
| 0.003 | -6.401e-07 |
| 0.004 | -6.523e-07 |
| 0.005 | -6.629e-07 |
| 0.006 | -6.705e-07 |
| 0.007 | -6.857e-07 |
| 0.008 | -6.835e-07 |
| 0.009 | -6.936e-07 |
| 0.01 | -7.05e-07 |
| 0.011 | -7.085e-07 |
| 0.012 | -7.126e-07 |
| 0.013 | -7.238e-07 |
| 0.014 | -7.247e-07 |
| 0.015 | -7.309e-07 |
| 0.016 | -7.352e-07 |
| 0.017 | -7.389e-07 |
| 0.018 | -7.405e-07 |
| 0.019 | -7.475e-07 |
| 0.02 | -7.474e-07 |
| 0.021 | -7.314e-07 |
| 0.022 | -7.43e-07 |
| 0.023 | -7.568e-07 |
| 0.024 | -7.563e-07 |
| 0.025 | -7.55e-07 |
| 0.026 | -7.633e-07 |
| 0.027 | -7.622e-07 |
| 0.028 | -7.625e-07 |
| 0.029 | -7.709e-07 |
| 0.03 | -7.664e-07 |
| 0.031 | -7.683e-07 |
| 0.032 | -7.71e-07 |
| 0.033 | -7.679e-07 |
| 0.034 | -7.62e-07 |
| 0.035 | -7.707e-07 |
| 0.036 | -7.717e-07 |
| 0.037 | -7.706e-07 |
| 0.038 | -7.705e-07 |
| 0.039 | -7.748e-07 |
| 0.04 | -7.743e-07 |
| 0.041 | -7.718e-07 |
| 0.042 | -7.763e-07 |
| 0.043 | -7.722e-07 |
| 0.044 | -7.712e-07 |
| 0.045 | -7.788e-07 |
| 0.046 | -7.73e-07 |
| 0.047 | -7.75e-07 |
| 0.048 | -7.736e-07 |
| 0.049 | -7.718e-07 |
| 0.05 | -7.705e-07 |
| 0.051 | -7.727e-07 |
| 0.052 | -7.72e-07 |
| 0.053 | -7.715e-07 |
| 0.054 | -7.691e-07 |
| 0.055 | -7.702e-07 |
| 0.056 | -7.664e-07 |
| 0.057 | -7.657e-07 |
| 0.058 | -7.723e-07 |
| 0.059 | -7.628e-07 |
| 0.06 | -7.619e-07 |
| 0.061 | -7.648e-07 |
| 0.062 | -7.627e-07 |
| 0.063 | -7.612e-07 |
| 0.064 | -7.634e-07 |
| 0.065 | -7.619e-07 |
| 0.066 | -7.628e-07 |
| 0.067 | -7.626e-07 |
| 0.068 | -7.594e-07 |
| 0.069 | -7.571e-07 |
| 0.07 | -7.58e-07 |
| 0.071 | -7.582e-07 |
| 0.072 | -7.621e-07 |
| 0.073 | -7.588e-07 |
| 0.074 | -7.584e-07 |
| 0.075 | -7.526e-07 |
| 0.076 | -7.506e-07 |
| 0.077 | -7.546e-07 |
| 0.078 | -7.501e-07 |
| 0.079 | -7.502e-07 |
| 0.08 | -7.52e-07 |
| 0.081 | -7.484e-07 |
| 0.082 | -7.455e-07 |
| 0.083 | -7.475e-07 |
| 0.084 | -7.469e-07 |
| 0.085 | -7.403e-07 |
| 0.086 | -7.42e-07 |
| 0.087 | -7.442e-07 |
| 0.088 | -7.425e-07 |
| 0.089 | -7.396e-07 |
| 0.09 | -7.441e-07 |
| 0.091 | -7.403e-07 |
| 0.092 | -7.404e-07 |
| 0.093 | -7.41e-07 |
| 0.094 | -7.378e-07 |
| 0.095 | -7.354e-07 |
| 0.096 | -7.384e-07 |
| 0.097 | -7.362e-07 |
| 0.098 | -7.392e-07 |
| 0.099 | -7.378e-07 |
| 0.1 | -7.357e-07 |
| 0.101 | -7.318e-07 |
| 0.102 | -7.314e-07 |
| 0.103 | -7.339e-07 |
| 0.104 | -7.348e-07 |
| 0.105 | -7.312e-07 |
| 0.106 | -7.334e-07 |
| 0.107 | -7.304e-07 |
| 0.108 | -7.295e-07 |
| 0.109 | -7.306e-07 |
| 0.11 | -7.285e-07 |
| 0.111 | -7.239e-07 |
| 0.112 | -7.295e-07 |
| 0.113 | -7.267e-07 |
| 0.114 | -7.263e-07 |
| 0.115 | -7.271e-07 |
| 0.116 | -7.282e-07 |
| 0.117 | -7.265e-07 |
| 0.118 | -7.266e-07 |
| 0.119 | -7.262e-07 |
| 0.12 | -7.253e-07 |
| 0.121 | -7.232e-07 |
| 0.122 | -7.278e-07 |
| 0.123 | -7.31e-07 |
| 0.124 | -7.302e-07 |
| 0.125 | -7.281e-07 |
| 0.126 | -7.259e-07 |
| 0.127 | -7.233e-07 |
| 0.128 | -7.284e-07 |
| 0.129 | -7.222e-07 |
| 0.13 | -7.245e-07 |
| 0.131 | -7.235e-07 |
| 0.132 | -7.273e-07 |
| 0.133 | -7.223e-07 |
| 0.134 | -7.254e-07 |
| 0.135 | -7.244e-07 |
| 0.136 | -7.226e-07 |
| 0.137 | -7.201e-07 |
| 0.138 | -7.263e-07 |
| 0.139 | -7.221e-07 |
| 0.14 | -7.237e-07 |
| 0.141 | -7.261e-07 |
| 0.142 | -7.251e-07 |
| 0.143 | -7.243e-07 |
| 0.144 | -7.297e-07 |
| 0.145 | -7.239e-07 |
| 0.146 | -7.254e-07 |
| 0.147 | -7.25e-07 |
| 0.148 | -7.285e-07 |
| 0.149 | -7.277e-07 |
| 0.15 | -7.295e-07 |
| 0.151 | -7.263e-07 |
| 0.152 | -7.279e-07 |
| 0.153 | -7.245e-07 |
| 0.154 | -7.321e-07 |
| 0.155 | -7.273e-07 |
| 0.156 | -7.293e-07 |
| 0.157 | -7.289e-07 |
| 0.158 | -7.316e-07 |
| 0.159 | -7.274e-07 |
| 0.16 | -7.329e-07 |
| 0.161 | -7.282e-07 |
| 0.162 | -7.29e-07 |
| 0.163 | -7.298e-07 |
| 0.164 | -7.338e-07 |
| 0.165 | -7.294e-07 |
| 0.166 | -7.356e-07 |
| 0.167 | -7.334e-07 |
| 0.168 | -7.373e-07 |
| 0.169 | -7.311e-07 |
| 0.17 | -7.416e-07 |
| 0.171 | -7.327e-07 |
| 0.172 | -7.371e-07 |
| 0.173 | -7.376e-07 |
| 0.174 | -7.442e-07 |
| 0.175 | -7.415e-07 |
| 0.176 | -7.452e-07 |
| 0.177 | -7.379e-07 |
| 0.178 | -7.424e-07 |
| 0.179 | -7.418e-07 |
| 0.18 | -7.461e-07 |
| 0.181 | -7.418e-07 |
| 0.182 | -7.468e-07 |
| 0.183 | -7.449e-07 |
| 0.184 | -7.476e-07 |
| 0.185 | -7.424e-07 |
| 0.186 | -7.516e-07 |
| 0.187 | -7.429e-07 |
| 0.188 | -7.483e-07 |
| 0.189 | -7.488e-07 |
| 0.19 | -7.534e-07 |
| 0.191 | -7.474e-07 |
| 0.192 | -7.56e-07 |
| 0.193 | -7.504e-07 |
| 0.194 | -7.576e-07 |
| 0.195 | -7.525e-07 |
| 0.196 | -7.6e-07 |
| 0.197 | -7.522e-07 |

| Potential (vs. Fc/Fc+) / V | DPV |
| --- | --- |
| -0.18 | -4.904e-07 |
| -0.176 | -4.973e-07 |
| -0.172 | -5.073e-07 |
| -0.168 | -5.196e-07 |
| -0.164 | -5.333e-07 |
| -0.16 | -5.477e-07 |
| -0.156 | -5.66e-07 |
| -0.152 | -5.818e-07 |
| -0.148 | -6.01e-07 |
| -0.144 | -6.208e-07 |
| -0.14 | -6.41e-07 |
| -0.136 | -6.636e-07 |
| -0.132 | -6.837e-07 |
| -0.128 | -7.049e-07 |
| -0.124 | -7.287e-07 |
| -0.12 | -7.484e-07 |
| -0.116 | -7.673e-07 |
| -0.112 | -7.846e-07 |
| -0.108 | -8.002e-07 |
| -0.104 | -8.166e-07 |
| -0.1 | -8.293e-07 |
| -0.096 | -8.423e-07 |
| -0.092 | -8.528e-07 |
| -0.088 | -8.617e-07 |
| -0.084 | -8.662e-07 |
| -0.08 | -8.748e-07 |
| -0.076 | -8.785e-07 |
| -0.072 | -8.86e-07 |
| -0.068 | -8.858e-07 |
| -0.064 | -8.875e-07 |
| -0.06 | -8.904e-07 |
| -0.056 | -8.922e-07 |
| -0.052 | -8.909e-07 |
| -0.048 | -8.895e-07 |
| -0.044 | -8.892e-07 |
| -0.04 | -8.851e-07 |
| -0.036 | -8.794e-07 |
| -0.032 | -8.699e-07 |
| -0.028 | -8.623e-07 |
| -0.024 | -8.528e-07 |
| -0.02 | -8.413e-07 |
| -0.016 | -8.281e-07 |
| -0.012 | -8.146e-07 |
| -0.008 | -7.954e-07 |
| -0.004 | -7.788e-07 |
| 0 | -7.601e-07 |
| 0.004 | -7.387e-07 |
| 0.008 | -7.192e-07 |
| 0.012 | -6.998e-07 |
| 0.016 | -6.811e-07 |
| 0.02 | -6.615e-07 |
| 0.024 | -6.435e-07 |
| 0.028 | -6.251e-07 |
| 0.032 | -6.068e-07 |
| 0.036 | -5.922e-07 |
| 0.04 | -5.784e-07 |
| 0.044 | -5.649e-07 |
| 0.048 | -5.523e-07 |
| 0.052 | -5.406e-07 |
| 0.056 | -5.306e-07 |
| 0.06 | -5.206e-07 |
| 0.064 | -5.125e-07 |
| 0.068 | -5.062e-07 |
| 0.072 | -4.998e-07 |
| 0.076 | -4.924e-07 |
| 0.08 | -4.89e-07 |
| 0.084 | -4.85e-07 |
| 0.088 | -4.803e-07 |
| 0.092 | -4.759e-07 |
| 0.096 | -4.769e-07 |
| 0.1 | -4.735e-07 |
| 0.104 | -4.723e-07 |
| 0.108 | -4.703e-07 |
| 0.112 | -4.708e-07 |
| 0.116 | -4.709e-07 |
| 0.12 | -4.717e-07 |
| 0.124 | -4.71e-07 |
| 0.128 | -4.723e-07 |
| 0.132 | -4.717e-07 |
| 0.136 | -4.746e-07 |
| 0.14 | -4.763e-07 |
| 0.144 | -4.769e-07 |
| 0.148 | -4.772e-07 |
| 0.152 | -4.806e-07 |
| 0.156 | -4.815e-07 |
| 0.16 | -4.838e-07 |
| 0.164 | -4.849e-07 |
| 0.168 | -4.845e-07 |
| 0.172 | -4.873e-07 |
| 0.176 | -4.888e-07 |
| 0.18 | -4.904e-07 |
| 0.184 | -4.925e-07 |
| 0.188 | -4.953e-07 |
| 0.192 | -4.973e-07 |
| 0.196 | -4.98e-07 |
| 0.2 | -5.01e-07 |
| 0.204 | -5.04e-07 |
| 0.208 | -5.06e-07 |
| 0.212 | -5.088e-07 |
| 0.216 | -5.119e-07 |
| 0.22 | -5.155e-07 |
| 0.224 | -5.186e-07 |
| 0.228 | -5.22e-07 |
| 0.232 | -5.27e-07 |
| 0.236 | -5.314e-07 |
| 0.24 | -5.347e-07 |
| 0.244 | -5.391e-07 |
| 0.248 | -5.436e-07 |
| 0.252 | -5.494e-07 |
| 0.256 | -5.547e-07 |
| 0.26 | -5.607e-07 |
| 0.264 | -5.678e-07 |
| 0.268 | -5.745e-07 |
| 0.272 | -5.814e-07 |
| 0.276 | -5.876e-07 |
| 0.28 | -5.955e-07 |
| 0.284 | -6.047e-07 |
| 0.288 | -6.143e-07 |
| 0.292 | -6.245e-07 |
| 0.296 | -6.351e-07 |
| 0.3 | -6.47e-07 |
| 0.304 | -6.594e-07 |
| 0.308 | -6.711e-07 |
| 0.312 | -6.885e-07 |
| 0.316 | -7.048e-07 |
| 0.32 | -7.213e-07 |
| 0.324 | -7.415e-07 |
| 0.328 | -7.61e-07 |
| 0.332 | -7.839e-07 |
| 0.336 | -8.08e-07 |
| 0.34 | -8.345e-07 |
| 0.344 | -8.654e-07 |
| 0.348 | -8.944e-07 |
| 0.352 | -9.301e-07 |
| 0.356 | -9.654e-07 |
| 0.36 | -1.01e-06 |
| 0.364 | -1.059e-06 |
| 0.368 | -1.111e-06 |
| 0.372 | -1.175e-06 |
| 0.376 | -1.25e-06 |
| 0.38 | -1.34e-06 |
| 0.384 | -1.441e-06 |
| 0.388 | -1.569e-06 |
| 0.392 | -1.726e-06 |
| 0.396 | -1.919e-06 |
| 0.4 | -2.157e-06 |
| 0.404 | -2.45e-06 |
| 0.408 | -2.815e-06 |
| 0.412 | -3.255e-06 |
| 0.416 | -3.798e-06 |
| 0.42 | -4.448e-06 |
| 0.424 | -5.233e-06 |
| 0.428 | -6.166e-06 |
| 0.432 | -7.268e-06 |
| 0.436 | -8.527e-06 |
| 0.44 | -9.967e-06 |
| 0.444 | -1.158e-05 |
| 0.448 | -1.333e-05 |
| 0.452 | -1.526e-05 |
| 0.456 | -1.733e-05 |
| 0.46 | -1.947e-05 |
| 0.464 | -2.166e-05 |
| 0.468 | -2.383e-05 |
| 0.472 | -2.594e-05 |
| 0.476 | -2.794e-05 |
| 0.48 | -2.975e-05 |
| 0.484 | -3.134e-05 |
| 0.488 | -3.263e-05 |
| 0.492 | -3.359e-05 |
| 0.496 | -3.418e-05 |
| 0.5 | -3.441e-05 |
| 0.504 | -3.426e-05 |
| 0.508 | -3.37e-05 |
| 0.512 | -3.277e-05 |
| 0.516 | -3.15e-05 |
| 0.52 | -2.996e-05 |
| 0.524 | -2.817e-05 |
| 0.528 | -2.62e-05 |
| 0.532 | -2.418e-05 |
| 0.536 | -2.213e-05 |
| 0.54 | -2.014e-05 |
| 0.544 | -1.827e-05 |
| 0.548 | -1.655e-05 |
| 0.552 | -1.499e-05 |
| 0.556 | -1.361e-05 |
| 0.56 | -1.237e-05 |
| 0.564 | -1.13e-05 |
| 0.568 | -1.036e-05 |
| 0.572 | -9.546e-06 |
| 0.576 | -8.813e-06 |
| 0.58 | -8.163e-06 |
| 0.584 | -7.597e-06 |
| 0.588 | -7.081e-06 |
| 0.592 | -6.606e-06 |
| 0.596 | -6.18e-06 |
| 0.6 | -5.788e-06 |
| 0.604 | -5.409e-06 |
| 0.608 | -5.048e-06 |
| 0.612 | -4.715e-06 |
| 0.616 | -4.384e-06 |

| Potential (vs. Fc/Fc+) / V | Current / A |
| --- | --- |
| -0.188 | 4.315e-07 |
| -0.192 | 4.233e-07 |
| -0.196 | 4.17e-07 |
| -0.2 | 4.108e-07 |
| -0.204 | 4.038e-07 |
| -0.208 | 4.006e-07 |
| -0.212 | 3.961e-07 |
| -0.216 | 3.929e-07 |
| -0.22 | 3.899e-07 |
| -0.224 | 3.875e-07 |
| -0.228 | 3.836e-07 |
| -0.232 | 3.822e-07 |
| -0.236 | 3.795e-07 |
| -0.24 | 3.788e-07 |
| -0.244 | 3.786e-07 |
| -0.248 | 3.767e-07 |
| -0.252 | 3.728e-07 |
| -0.256 | 3.737e-07 |
| -0.26 | 3.716e-07 |
| -0.264 | 3.703e-07 |
| -0.268 | 3.702e-07 |
| -0.272 | 3.69e-07 |
| -0.276 | 3.692e-07 |
| -0.28 | 3.689e-07 |
| -0.284 | 3.67e-07 |
| -0.288 | 3.677e-07 |
| -0.292 | 3.665e-07 |
| -0.296 | 3.669e-07 |
| -0.3 | 3.669e-07 |
| -0.304 | 3.671e-07 |
| -0.308 | 3.672e-07 |
| -0.312 | 3.677e-07 |
| -0.316 | 3.684e-07 |
| -0.32 | 3.678e-07 |
| -0.324 | 3.688e-07 |
| -0.328 | 3.693e-07 |
| -0.332 | 3.7e-07 |
| -0.336 | 3.713e-07 |
| -0.34 | 3.706e-07 |
| -0.344 | 3.721e-07 |
| -0.348 | 3.739e-07 |
| -0.352 | 3.751e-07 |
| -0.356 | 3.762e-07 |
| -0.36 | 3.785e-07 |
| -0.364 | 3.796e-07 |
| -0.368 | 3.82e-07 |
| -0.372 | 3.851e-07 |
| -0.376 | 3.861e-07 |
| -0.38 | 3.893e-07 |
| -0.384 | 3.908e-07 |
| -0.388 | 3.931e-07 |
| -0.392 | 3.976e-07 |
| -0.396 | 3.999e-07 |
| -0.4 | 4.028e-07 |
| -0.404 | 4.054e-07 |
| -0.408 | 4.087e-07 |
| -0.412 | 4.116e-07 |
| -0.416 | 4.158e-07 |
| -0.42 | 4.178e-07 |
| -0.424 | 4.217e-07 |
| -0.428 | 4.255e-07 |
| -0.432 | 4.276e-07 |
| -0.436 | 4.309e-07 |
| -0.44 | 4.335e-07 |
| -0.444 | 4.367e-07 |
| -0.448 | 4.396e-07 |
| -0.452 | 4.429e-07 |
| -0.456 | 4.462e-07 |
| -0.46 | 4.49e-07 |
| -0.464 | 4.508e-07 |
| -0.468 | 4.527e-07 |
| -0.472 | 4.566e-07 |
| -0.476 | 4.58e-07 |
| -0.48 | 4.608e-07 |
| -0.484 | 4.627e-07 |
| -0.488 | 4.656e-07 |
| -0.492 | 4.68e-07 |
| -0.496 | 4.691e-07 |
| -0.5 | 4.715e-07 |
| -0.504 | 4.74e-07 |
| -0.508 | 4.756e-07 |
| -0.512 | 4.772e-07 |
| -0.516 | 4.795e-07 |
| -0.52 | 4.811e-07 |
| -0.524 | 4.836e-07 |
| -0.528 | 4.848e-07 |
| -0.532 | 4.862e-07 |
| -0.536 | 4.875e-07 |
| -0.54 | 4.894e-07 |
| -0.544 | 4.907e-07 |
| -0.548 | 4.924e-07 |
| -0.552 | 4.943e-07 |
| -0.556 | 4.956e-07 |
| -0.56 | 4.967e-07 |
| -0.564 | 4.984e-07 |
| -0.568 | 4.99e-07 |
| -0.572 | 5.006e-07 |
| -0.576 | 5.019e-07 |
| -0.58 | 5.041e-07 |
| -0.584 | 5.061e-07 |
| -0.588 | 5.071e-07 |
| -0.592 | 5.085e-07 |
| -0.596 | 5.106e-07 |
| -0.6 | 5.14e-07 |
| -0.604 | 5.155e-07 |
| -0.608 | 5.172e-07 |
| -0.612 | 5.194e-07 |
| -0.616 | 5.225e-07 |
| -0.62 | 5.268e-07 |
| -0.624 | 5.283e-07 |
| -0.628 | 5.313e-07 |
| -0.632 | 5.348e-07 |
| -0.636 | 5.381e-07 |
| -0.64 | 5.404e-07 |
| -0.644 | 5.427e-07 |
| -0.648 | 5.475e-07 |
| -0.652 | 5.488e-07 |
| -0.656 | 5.511e-07 |
| -0.66 | 5.537e-07 |
| -0.664 | 5.552e-07 |
| -0.668 | 5.584e-07 |
| -0.672 | 5.6e-07 |
| -0.676 | 5.598e-07 |
| -0.68 | 5.615e-07 |
| -0.684 | 5.631e-07 |
| -0.688 | 5.645e-07 |
| -0.692 | 5.652e-07 |
| -0.696 | 5.664e-07 |
| -0.7 | 5.688e-07 |
| -0.704 | 5.701e-07 |
| -0.708 | 5.712e-07 |
| -0.712 | 5.751e-07 |
| -0.716 | 5.762e-07 |
| -0.72 | 5.788e-07 |
| -0.724 | 5.822e-07 |
| -0.728 | 5.86e-07 |
| -0.732 | 5.884e-07 |
| -0.736 | 5.912e-07 |
| -0.74 | 5.944e-07 |
| -0.744 | 5.977e-07 |
| -0.748 | 6.004e-07 |
| -0.752 | 6.039e-07 |
| -0.756 | 6.069e-07 |
| -0.76 | 6.115e-07 |
| -0.764 | 6.14e-07 |
| -0.768 | 6.178e-07 |
| -0.772 | 6.211e-07 |
| -0.776 | 6.248e-07 |
| -0.78 | 6.29e-07 |
| -0.784 | 6.33e-07 |
| -0.788 | 6.372e-07 |
| -0.792 | 6.405e-07 |
| -0.796 | 6.443e-07 |
| -0.8 | 6.479e-07 |
| -0.804 | 6.526e-07 |
| -0.808 | 6.554e-07 |
| -0.812 | 6.588e-07 |
| -0.816 | 6.629e-07 |
| -0.82 | 6.657e-07 |
| -0.824 | 6.684e-07 |
| -0.828 | 6.728e-07 |
| -0.832 | 6.756e-07 |
| -0.836 | 6.791e-07 |
| -0.84 | 6.802e-07 |
| -0.844 | 6.839e-07 |
| -0.848 | 6.855e-07 |
| -0.852 | 6.889e-07 |
| -0.856 | 6.909e-07 |
| -0.86 | 6.927e-07 |
| -0.864 | 6.943e-07 |
| -0.868 | 6.97e-07 |
| -0.872 | 6.996e-07 |
| -0.876 | 7.009e-07 |
| -0.88 | 7.041e-07 |
| -0.884 | 7.036e-07 |
| -0.888 | 7.055e-07 |
| -0.892 | 7.073e-07 |
| -0.896 | 7.105e-07 |
| -0.9 | 7.115e-07 |
| -0.904 | 7.126e-07 |
| -0.908 | 7.126e-07 |
| -0.912 | 7.155e-07 |
| -0.916 | 7.164e-07 |
| -0.92 | 7.162e-07 |
| -0.924 | 7.178e-07 |
| -0.928 | 7.186e-07 |
| -0.932 | 7.204e-07 |
| -0.936 | 7.2e-07 |
| -0.94 | 7.221e-07 |
| -0.944 | 7.201e-07 |
| -0.948 | 7.189e-07 |
| -0.952 | 7.176e-07 |
| -0.956 | 7.166e-07 |
| -0.96 | 7.158e-07 |
| -0.964 | 7.132e-07 |
| -0.968 | 7.113e-07 |
| -0.972 | 7.099e-07 |
| -0.976 | 7.091e-07 |
| -0.98 | 7.076e-07 |
| -0.984 | 7.076e-07 |

**Figure SI-9** Cyclic and differential pulse voltammograms of **1c**.

**Table SI-2** Summary of electrochemical studies

(V, vs ferrocene/ferrocenium, in CH_2_Cl_2_, TBAPF_6_ as supporting electrolyte.).

| **Compound** | **Ox2** | **Ox1** | **Red1** | **Red2** |
| --- | --- | --- | --- | --- |
| SubPc(BINOLate) | 0.71 V | 0.46 V | -1.57 V | -2.00 V |
| SubPc(O-MeBINOLate) |  | 0.50 V | -1.56 V | -2.02 V |

§3 Absorption/CD spectra of **1c** and their simulations.

All quantum chemical calculations were carried out using the B3LYP functional in combination with the 6-31G(d) basis set. The optimized coordinates of (*S*)-**1c** are as follows.

O	-0.11956200	0.03675205	-1.26359689

N	1.88677425	-1.08892925	-0.55609071

O	-2.89234318	0.39141009	1.66784289

N	0.65839621	0.04244330	1.12314016

N	1.92131975	1.27383867	-0.44827136

N	1.09701154	-2.28724637	1.33697939

N	1.18211371	2.32222756	1.55357536

N	3.54883864	0.12185039	-1.74772669

C	0.28450502	-0.76472339	3.19286306

C	1.79590152	-2.22917186	0.19349008

C	0.61030242	-1.13505778	1.82132100

C	1.87616417	2.34397294	0.40592991

C	3.58375439	-2.37548176	-1.28372732

C	0.31194910	0.66341698	3.26084242

C	0.65277537	1.14870406	1.92978359

C	3.01094245	-1.03573962	-1.33407402

C	2.82406512	-3.12168735	-0.32808509

C	3.66336834	2.56316523	-1.06233169

C	-1.10354385	0.97918885	-1.38739441

C	3.04579967	1.25435982	-1.23212886

C	0.06975836	-1.52640240	4.34423124

H	0.06937970	-2.61085160	4.29344235

C	2.93199553	3.24425223	-0.03790380

C	-2.40727121	0.67002217	-1.01819785

C	-3.44018520	1.64257702	-1.23304362

C	-0.78942229	2.23502830	-1.97402582

H	0.23388691	2.42170501	-2.28181525

C	-3.11187044	2.91774319	-1.80077967

C	-0.11226035	0.54922445	5.61470767

H	-0.27259437	1.03895802	6.57121158

C	-2.72752459	-0.66381046	-0.42191413

C	-1.76573716	3.18008195	-2.16440233

H	-1.51799912	4.14013743	-2.61140589

C	4.70232439	-2.94146775	-1.89919601

H	5.28790910	-2.36793382	-2.61100470

C	4.80056331	3.14489086	-1.62863805

H	5.36495691	2.61887984	-2.39239151

C	3.18854027	-4.42812302	0.00424677

H	2.62058880	-4.98680173	0.74163708

C	0.12764219	1.32006518	4.47984507

H	0.17047457	2.40358242	4.53249525

C	-0.14168769	-0.85746759	5.54771923

H	-0.32373066	-1.42889012	6.45372291

C	3.34284572	4.50193066	0.41093844

H	2.79677668	5.00930440	1.20017033

C	-2.56316216	-1.81394751	-2.63538576

H	-2.32020742	-0.86808824	-3.10692610

C	5.03711222	-4.25453007	-1.57847288

H	5.89311353	-4.72130333	-2.05773683

C	5.18138509	4.41009136	-1.19042199

H	6.05271302	4.88887017	-1.62845107

C	4.28932753	-4.98890880	-0.63831962

H	4.58103056	-6.00985602	-0.40836728

C	-4.13816975	3.87844232	-2.00352402

H	-3.86944406	4.84073380	-2.43406089

C	4.46106338	5.08069870	-0.18274560

H	4.78923553	6.06514191	0.13893519

C	-4.80212493	1.39012518	-0.90802235

H	-5.06982519	0.42380924	-0.49396411

C	-2.98523028	-0.76579528	0.94341912

C	-2.79548556	-1.84098215	-1.23044710

C	-5.44405716	3.60286207	-1.66917100

H	-6.22082808	4.34566561	-1.83009962

C	-3.12391244	-3.10199006	-0.62881459

C	-5.77513527	2.34165244	-1.11944332

H	-6.80868776	2.12126787	-0.86452320

C	-3.31302182	-2.01194437	1.53635218

H	-3.50487891	-2.07479192	2.60121358

C	-3.37762135	0.40650576	3.00176179

H	-4.42583037	0.08337208	3.05194785

H	-3.30416552	1.44492316	3.32971722

H	-2.76984190	-0.21999964	3.66615694

C	-3.37641592	-3.14755109	0.76330888

H	-3.62480813	-4.10147615	1.22278815

B	0.98655172	0.09346913	-0.34889839

C	-3.19533238	-4.26764928	-1.43892022

H	-3.44315143	-5.21459081	-0.96402046

C	-2.64351155	-2.96054382	-3.39172392

H	-2.46122770	-2.91240326	-4.46222125

C	-2.96005464	-4.20433116	-2.79195526

H	-3.01762598	-5.10146807	-3.40247056

Using the above optimized coordinates, TDDFT calculations were carried out at the B3LYP/6-31G(d) level. The results are plotted together with the experimentally obtained spectra (*rac*-**1c**) measured in CH₂Cl₂.

**Figure SI-10** Absorption spectra of *rac*-**1c** and TDDFT simulation.

**Figure SI-11** CD spectra of optically pure **1c** and the corresponding simulated spectra obtained at the B3LYP/6-31G(d) level.

§4 Structural optimization and TDDFT studies for **1b**

For structural optimization, the initial geometry was taken from the atomic coordinates determined by single-crystal X-ray diffraction. The optimized coordinates of **1b** are as follows.

O	-0.05132200	0.08372800	-1.54385300

O	4.70365000	-0.19416100	0.16662200

N	-0.31759400	-0.05552400	0.95138600

N	-2.08091000	-0.70230700	-0.49205200

N	-1.55559000	1.58838400	-0.20910000

N	-0.20536600	2.21676000	1.64470000

N	-3.62946500	0.96069200	-1.19224000

N	-1.22231500	-2.25095200	1.09419400

C	-2.10452000	-1.91538200	0.14057300

C	-0.39707100	-1.29189700	1.53752300

C	-3.29774800	-0.32422100	-0.99296000

C	-1.09444600	2.51737000	0.68528500

C	2.68467400	-1.05465300	-0.87568300

C	-3.39343000	-2.50531500	-0.19778600

C	0.28619800	-1.16224500	2.81851700

C	0.10656300	0.92331900	1.81151800

C	-4.13932000	-1.51102600	-0.90707700

C	2.35141200	0.32149100	-1.30383500

C	-1.98422800	3.66317000	0.55293100

C	-3.04355400	3.27486300	-0.32770900

C	-2.78864300	1.89588200	-0.72319300

C	1.07701900	-2.32824100	-2.35212900

H	0.86204300	-1.43558500	-2.91961800

C	2.00384900	-2.26755800	-1.27534400

C	0.60187700	0.22208400	2.98952000

C	1.09877300	0.81384300	-1.68409100

C	-3.97307900	-3.73600700	0.11896200

H	-3.41082700	-4.48203900	0.67189300

C	3.43250200	1.26785900	-1.27715700

C	0.56045100	-2.08347600	3.83155400

H	0.30458100	-3.13089600	3.70606000

C	1.19007300	0.67576300	4.17212000

H	1.41385700	1.72945100	4.30641500

C	2.34066100	-3.50798600	-0.63206400

C	3.81626800	-1.21105300	-0.07681700

C	4.62393100	0.95856100	-0.57752700

C	-5.45929900	-1.75548000	-1.29326000

H	-6.02995900	-0.99358600	-1.81519500

C	0.98670800	2.10036700	-2.27666300

H	0.00604500	2.39694600	-2.63554700

C	3.32432400	2.56785300	-1.86023600

C	2.06669800	2.93789800	-2.40587300

H	1.95791600	3.90860400	-2.88258200

C	-6.01416900	-2.99704800	-0.99476800

H	-7.03370400	-3.21536800	-1.30007500

C	-4.10785100	4.14327900	-0.58332700

H	-4.92013400	3.84037400	-1.23679800

C	-5.27992900	-3.97558000	-0.29718500

H	-5.74570700	-4.93194400	-0.07641700

C	-1.99717700	4.91663600	1.16975600

H	-1.20156300	5.20230500	1.85084300

C	4.15440900	-2.42814100	0.55424500

H	5.01678500	-2.44066500	1.21268000

C	0.46416900	-3.51116500	-2.70864100

H	-0.22874900	-3.52022500	-3.54591900

C	1.48288700	-0.25841500	5.16229000

H	1.95588700	0.06865500	6.08401500

C	1.17171600	-1.62131500	4.99411700

H	1.40976300	-2.32236300	5.78923800

C	5.69850000	1.82318300	-0.53477000

H	6.58521000	1.53932900	0.02222300

C	3.39702700	-3.54373400	0.31717600

H	3.63426400	-4.48425500	0.80780100

C	0.73350500	-4.70845000	-2.01009000

H	0.23083100	-5.62993800	-2.29093500

C	1.66429800	-4.70106900	-0.99736400

H	1.92270500	-5.62008300	-0.47608900

C	-3.04993100	5.78127000	0.88337400

H	-3.07256600	6.76719400	1.33914300

C	-4.09286000	5.39903200	0.01730700

H	-4.90198000	6.09672700	-0.17985200

C	5.60496100	3.06902100	-1.18859300

H	6.45344100	3.74698800	-1.15925400

C	4.44164300	3.44216900	-1.82790300

H	4.35349300	4.42158600	-2.29055800

B	-0.91772300	0.23603200	-0.39803500

**Figure SI-12** Absorption spectra of **1b** and TDDFT simulation.

§5 Dihedral angle dependence of the CD behavior of dimethoxy BINOL.

The dihedral angle dependence of the CD behavior of dimethoxy BINOL was investigated as follows.

Structural optimizations were carried out by constraining the torsion angle ∠C2–C1–C1′–C2′ from 0° to 180° in 10° increments. The definition of the angle for the (*S*)-isomer is shown in the following figure. The dihedral angle *θ* of BINOL was evaluated as the angle between the least-squares planes defined by ten carbon atoms of the naphthalene rings. For example, for a structure with ∠C2–C1–C1′–C2′ constrained to 110°, the resulting *θ* was 107.09°. Using these optimized coordinates, TDDFT calculations were performed.

The most stable structure was obtained starting from initial coordinates with acute and/or obtuse angles, and the dihedral angle of the optimized structure was *θ =* 89.1°

**Figure SI-13** Definition of the torsion angles (dihedral) of dimethoxy BINOL.

5-1 Optimized coordinate of dimethoxy BINOL with *θ* = 47.22° (∠C2-C1-C1’-C2’ = 0°).

O	0.64880800	1.06644000	2.57710900

O	-0.64880800	-1.06644000	2.57710900

C	0.00000000	1.56453100	1.48122700

C	0.00000000	0.75396300	0.32575600

C	-0.74529500	1.36350500	-0.79247100

C	-0.32711200	2.94638600	1.46042500

H	-0.18945100	3.53707600	2.35718400

C	-1.00680200	2.77455700	-0.84160800

C	0.00000000	-0.75396300	0.32575600

C	-0.76946500	3.54932000	0.31617900

H	-0.98285600	4.61547400	0.30122200

C	1.32026400	-0.60487800	-1.85756700

H	1.28288700	0.47435500	-1.82018200

C	-1.61581800	3.36869400	-1.98031600

H	-1.75115700	4.44812900	-1.98397800

C	-1.32026400	0.60487800	-1.85756700

H	-1.28288700	-0.47435500	-1.82018200

C	0.00000000	-1.56453100	1.48122700

C	0.74529500	-1.36350500	-0.79247100

C	-2.06757800	2.60065800	-3.02546300

H	-2.54390600	3.05769900	-3.88838400

C	1.00680200	-2.77455700	-0.84160800

C	-1.95180800	1.19634200	-2.93027100

H	-2.37605100	0.56626900	-3.70787500

C	0.32711200	-2.94638600	1.46042500

H	0.18945100	-3.53707600	2.35718400

C	-0.64292900	-1.77968800	3.79839200

H	-1.25255200	-2.69243200	3.74709000

H	-1.08463500	-1.10626500	4.53638900

H	0.37423000	-2.04416400	4.11597100

C	0.76946500	-3.54932000	0.31617900

H	0.98285600	-4.61547400	0.30122200

C	1.61581800	-3.36869400	-1.98031600

H	1.75115700	-4.44812900	-1.98397800

C	1.95180800	-1.19634200	-2.93027100

H	2.37605100	-0.56626900	-3.70787500

C	2.06757800	-2.60065800	-3.02546300

H	2.54390600	-3.05769900	-3.88838400

C	0.64292900	1.77968800	3.79839200

H	1.08463500	1.10626500	4.53638900

H	1.25255200	2.69243200	3.74709000

H	-0.37423000	2.04416400	4.11597100

**Figure SI-14** Optimized structure with the torsion angle ∠C2–C1–C1′–C2′ constrained to 0°. The dihedral angle between the naphthalene rings is *θ* = 47.22°.

5-2 The coordinates of the most stable dimethoxy BINOL. The dihedral angle is *θ* = 89.06°.

O	1.63810500	0.66874000	1.94163600

O	-1.63810500	-0.66874000	1.94163600

C	0.84316200	1.43609300	1.13680500

C	-0.00128800	0.74787600	0.27002300

C	-0.86177400	1.48587000	-0.59750400

C	0.86237400	2.85396900	1.16377400

H	1.52489700	3.37935600	1.84179700

C	-0.84316200	2.92084700	-0.56925600

C	0.00128800	-0.74787600	0.27002300

C	0.03632800	3.57092900	0.32915600

H	0.05491400	4.65804300	0.35434500

C	1.75302400	-0.84726800	-1.50731400

H	1.77690400	0.23700200	-1.54015600

C	-1.70154800	3.64870400	-1.43729100

H	-1.67412300	4.73573700	-1.40243400

C	-1.75302400	0.84726800	-1.50731400

H	-1.77690400	-0.23700200	-1.54015600

C	-0.84316200	-1.43609300	1.13680500

C	0.86177400	-1.48587000	-0.59750400

C	-2.54913900	2.99869300	-2.30317600

H	-3.20092900	3.56522700	-2.96271200

C	0.84316200	-2.92084700	-0.56925600

C	-2.57100100	1.58224800	-2.33444800

H	-3.24190300	1.07150100	-3.02048200

C	-0.86237400	-2.85396900	1.16377400

H	-1.52489700	-3.37935600	1.84179700

C	-2.52762600	-1.30785400	2.84027800

H	-3.26359500	-1.93035300	2.31376600

H	-3.04929200	-0.50310100	3.36199000

H	-1.99290800	-1.92533400	3.57452600

C	-0.03632800	-3.57092900	0.32915600

H	-0.05491400	-4.65804300	0.35434500

C	1.70154800	-3.64870400	-1.43729100

H	1.67412300	-4.73573700	-1.40243400

C	2.57100100	-1.58224800	-2.33444800

H	3.24190300	-1.07150100	-3.02048200

C	2.54913900	-2.99869300	-2.30317600

H	3.20092900	-3.56522700	-2.96271200

C	2.52762600	1.30785400	2.84027800

H	3.04929200	0.50310100	3.36199000

H	3.26359500	1.93035300	2.31376600

H	1.99290800	1.92533400	3.57452600

**Figure SI-15** Optimized most stable structure with *θ* = 89.06°.

5-3 Optimized coordinate of dimethoxy BINOL with *θ* = 107.09°.

This angle is close to that observed in the structure of **1c.**

O	1.90164600	0.66384100	1.62695600

O	-1.90164600	-0.66384100	1.62695600

C	0.99730100	1.43335300	0.94908600

C	0.00246600	0.74761900	0.25408200

C	-0.97031800	1.49546500	-0.48024500

C	1.04125100	2.85070700	0.95811600

H	1.81552100	3.36990200	1.51083800

C	-0.92875300	2.92996100	-0.46433400

C	-0.00246600	-0.74761900	0.25408200

C	0.09499800	3.57409600	0.27067000

H	0.13018500	4.66096800	0.28425700

C	1.98992500	-0.86993000	-1.25370500

H	2.02388500	0.21332000	-1.29506600

C	-1.90164600	3.66867700	-1.19123500

H	-1.85464700	4.75521000	-1.16286700

C	-1.98992500	0.86993000	-1.25370500

H	-2.02388500	-0.21332000	-1.29506600

C	-0.99730100	-1.43335300	0.94908600

C	0.97031800	-1.49546500	-0.48024500

C	-2.87763100	3.02999900	-1.91872200

H	-3.61553800	3.60416600	-2.47243200

C	0.92875300	-2.92996100	-0.46433400

C	-2.91367100	1.61413100	-1.95085400

H	-3.67900000	1.11054000	-2.53601900

C	-1.04125100	-2.85070700	0.95811600

H	-1.81552100	-3.36990200	1.51083800

C	-2.95137400	-1.29652800	2.33775600

H	-3.59259000	-1.89328600	1.67520700

H	-3.54186600	-0.48804900	2.77297100

H	-2.57019200	-1.93784400	3.14378300

C	-0.09499800	-3.57409600	0.27067000

H	-0.13018500	-4.66096800	0.28425700

C	1.90164600	-3.66867700	-1.19123500

H	1.85464700	-4.75521000	-1.16286700

C	2.91367100	-1.61413100	-1.95085400

H	3.67900000	-1.11054000	-2.53601900

C	2.87763100	-3.02999900	-1.91872200

H	3.61553800	-3.60416600	-2.47243200

C	2.95137400	1.29652800	2.33775600

H	3.54186600	0.48804900	2.77297100

H	3.59259000	1.89328600	1.67520700

H	2.57019200	1.93784400	3.14378300

**Figure SI-16** Optimized structure with the torsion angle ∠C2–C1–C1′–C2′ constrained to 110°. The dihedral angle between the naphthalene rings is *θ* = 107.09°.

5-4 Optimized coordinate of dimethoxy BINOL with *θ* = 107.09°. The dihedral 135.49

O	-2.22500800	0.94381000	-1.18855100

O	2.22500800	-0.94381000	-1.18855100

C	-1.13850800	1.53250200	-0.58128700

C	0.03648000	0.75487000	-0.58128500

C	1.16089000	1.44079300	0.08107300

C	-1.16089000	2.93427200	-0.36580500

H	-2.08668900	3.47957700	-0.49922000

C	1.18133500	2.87677500	0.17608600

C	-0.03648000	-0.75487000	-0.58128500

C	-0.00623200	3.60308100	-0.07160800

H	-0.00266200	4.68565700	0.02853400

C	-2.22074900	-0.79363300	0.78403200

H	-2.15948300	0.26669000	0.97795300

C	2.32490500	3.56500200	0.66697900

H	2.30941000	4.65262300	0.65719100

C	2.22074900	0.79363300	0.78403200

H	2.15948300	-0.26669000	0.97795300

C	1.13850800	-1.53250200	-0.58128700

C	-1.16089000	-1.44079300	0.08107300

C	3.38910200	2.88273400	1.20231300

H	4.25449900	3.41160300	1.59179400

C	-1.18133500	-2.87677500	0.17608600

C	3.29058400	1.47917100	1.31827700

H	4.05508100	0.92836400	1.86085200

C	1.16089000	-2.93427200	-0.36580500

H	2.08668900	-3.47957700	-0.49922000

C	3.47958600	-1.59845400	-1.20426400

H	3.79903800	-1.90591000	-0.19906300

H	4.18896700	-0.86520700	-1.59275000

H	3.47397500	-2.47658800	-1.86394500

C	0.00623200	-3.60308100	-0.07160800

H	0.00266200	-4.68565700	0.02853400

C	-2.32490500	-3.56500200	0.66697900

H	-2.30941000	-4.65262300	0.65719100

C	-3.29058400	-1.47917100	1.31827700

H	-4.05508100	-0.92836400	1.86085200

C	-3.38910200	-2.88273400	1.20231300

H	-4.25449900	-3.41160300	1.59179400

C	-3.47958600	1.59845400	-1.20426400

H	-4.18896700	0.86520700	-1.59275000

H	-3.79903800	1.90591000	-0.19906300

H	-3.47397500	2.47658800	-1.86394500

**Figure SI-17** Optimized structure with the torsion angle ∠C2–C1–C1′–C2′ constrained to 180°. The dihedral angle between the naphthalene rings is *θ* = 135.49°.

5-5 Dihedral angle–dependent CD spectra of dimethoxy BINOL

**Figure SI-18** Simulated CD spectra of dimethoxy BINOL as a function of the dihedral angle between the naphthalene rings, defined by ten carbon atoms, calculated at the B3LYP/6-31G(d) level. The most stable structure has a dihedral angle of θ = 88°. The optimized coordinates of dimethoxy BINOL at θ = 0°, 88°, 110°, and 180° are shown below. We also optimized the structures and carried out TDDFT calculations at θ = 20°, 30°, 40°, 50°, 60°, 70°, 80°, 90°, 100°, 120°, 130°, 140°, 150°, and 170°; however, these results are omitted. The TDDFT results for these angles are plotted as pale dotted lines in the above figure.

§6 Single Crystal Diffraction Studies

6-1 **1b**

**Figure SI-19** Molecular structure of **1b**. Thermal ellipsoids are shown at the 50% probability level.

| **Table 6-1-1** Crystal data and structure refinement for **1b**. | |
| --- | --- |
| Identification code | **1b** |
| Empirical formula | C_44_H_23_BN_6_O_2_ |
| Formula weight | 678.49 |
| Temperature/K | 100 |
| Crystal system | monoclinic |
| Space group | P2_1_/c |
| a/Å | 15.2940(3) |
| b/Å | 10.1912(2) |
| c/Å | 21.3972(4) |
| α/° | 90 |
| β/° | 106.137(2) |
| γ/° | 90 |
| Volume/Å^3^ | 3203.66(11) |
| Z | 4 |
| ρ_calc_g/cm^3^ | 1.407 |
| μ/mm^‑1^ | 0.706 |
| F(000) | 1400.0 |
| Crystal size/mm^3^ | 0.6 × 0.2 × 0.15 |
| Radiation | Cu Kα (λ = 1.54184) |
| 2Θ range for data collection/° | 6.016 to 142.444 |
| Index ranges | -18 ≤ h ≤ 12, -12 ≤ k ≤ 12, -25 ≤ l ≤ 25 |
| Reflections collected | 17876 |
| Independent reflections | 6039 [R_int_ = 0.0418, R_sigma_ = 0.0430] |
| Data/restraints/parameters | 6039/0/478 |
| Goodness-of-fit on F^2^ | 1.038 |
| Final R indexes [I>=2σ (I)] | R_1_ = 0.0440, wR_2_ = 0.1207 |
| Final R indexes [all data] | R_1_ = 0.0503, wR_2_ = 0.1253 |
| Largest diff. peak/hole / e Å^-3^ | 0.30/-0.29 |

| **Table 6-1-2** Fractional Atomic Coordinates (×10^4^) and Equivalent Isotropic Displacement Parameters (Å^2^×10^3^) for dt01_auto. U_eq_ is defined as 1/3 of the trace of the orthogonalised U_IJ_ tensor. | | | | |
| --- | --- | --- | --- | --- |
| **Atom** | ***x*** | ***y*** | ***z*** | **U(eq)** |
| O1 | 3127.9(6) | 6799.8(9) | 6558.6(5) | 22.0(2) |
| O2 | 985.9(7) | 3352.9(11) | 6901.4(5) | 30.8(3) |
| N5 | 2644.9(8) | 4985.1(11) | 5747.0(6) | 20.7(2) |
| N1 | 4075.5(8) | 5974.0(11) | 5899.5(6) | 21.2(3) |
| N3 | 2744.0(8) | 7124.0(11) | 5360.8(6) | 21.7(3) |
| N4 | 1355.3(8) | 5932.9(12) | 4994.4(6) | 23.7(3) |
| N2 | 4177.7(8) | 7851.0(12) | 5266.8(6) | 23.8(3) |
| N6 | 3979.8(8) | 3670.6(12) | 6052.4(6) | 22.7(3) |
| C1 | 4463.6(10) | 4759.4(14) | 6027.0(7) | 22.5(3) |
| C24 | 3067.7(9) | 3797.5(14) | 5867.8(7) | 21.4(3) |
| C8 | 4563.1(10) | 6817.4(14) | 5633.9(7) | 22.3(3) |
| C16 | 1870.1(10) | 6996.7(14) | 4974.8(7) | 23.1(3) |
| C35 | 2431.4(10) | 4491.5(14) | 7145.3(7) | 22.8(3) |
| C2 | 5387.6(10) | 4914.8(15) | 5971.9(7) | 24.0(3) |
| C23 | 2353.2(10) | 2826.2(14) | 5625.7(7) | 23.0(3) |
| C17 | 1771.3(9) | 4913.5(14) | 5355.2(7) | 21.6(3) |
| C7 | 5445.5(10) | 6199.1(15) | 5723.3(7) | 23.7(3) |
| C34 | 1942.0(10) | 5715.9(14) | 6906.0(7) | 23.0(3) |
| C15 | 1757.3(10) | 8009.3(14) | 4482.3(7) | 25.1(3) |
| C10 | 2626.6(10) | 8590.6(15) | 4560.8(7) | 25.7(3) |
| C9 | 3264.5(10) | 7935.5(14) | 5105.2(7) | 23.3(3) |
| C43 | 3926.4(10) | 5518.7(15) | 7734.8(7) | 26.1(3) |
| C44 | 3387.0(10) | 4406.5(15) | 7480.1(7) | 24.5(3) |
| C18 | 1542.8(10) | 3527.4(14) | 5309.7(7) | 23.4(3) |
| C25 | 2295.5(10) | 6821.5(14) | 6686.4(7) | 22.6(3) |
| C3 | 6128.4(10) | 4064.3(16) | 6093.1(7) | 27.5(3) |
| C33 | 999.7(10) | 5727.8(15) | 6876.5(7) | 25.5(3) |
| C22 | 2356.7(11) | 1463.3(15) | 5624.8(7) | 27.1(3) |
| C19 | 746.5(10) | 2870.8(15) | 4992.2(8) | 28.3(3) |
| C39 | 3800.6(11) | 3146.5(15) | 7607.5(7) | 27.8(3) |
| C36 | 1925.3(10) | 3350.6(15) | 7091.7(7) | 26.6(3) |
| C32 | 544.1(10) | 4534.6(16) | 6912.2(7) | 27.8(3) |
| C6 | 6243.9(10) | 6625.5(16) | 5593.6(7) | 27.5(3) |
| C26 | 1788.5(10) | 8004.0(15) | 6576.3(7) | 27.8(3) |
| C28 | 483.8(11) | 6910.6(16) | 6771.6(7) | 29.2(3) |
| C27 | 926.9(11) | 8063.8(16) | 6642.6(8) | 31.5(4) |
| C5 | 6981.2(10) | 5782.2(17) | 5739.1(7) | 30.4(3) |
| C11 | 2750.3(12) | 9552.4(16) | 4128.3(8) | 30.9(3) |
| C4 | 6924.8(10) | 4524.3(17) | 5986.8(8) | 30.5(3) |
| C14 | 1007.0(11) | 8392.8(16) | 3975.3(7) | 29.9(3) |
| C37 | 2327.5(12) | 2103.6(16) | 7226.8(8) | 31.3(4) |
| C42 | 4834.8(11) | 5396.3(17) | 8059.6(8) | 31.7(4) |
| C20 | 761.2(11) | 1515.8(16) | 5012.1(8) | 31.8(4) |
| C21 | 1549.6(11) | 822.2(15) | 5325.8(8) | 31.1(3) |
| C31 | -347.7(11) | 4507.7(18) | 6919.7(8) | 32.9(4) |
| C38 | 3251.4(12) | 2006.8(16) | 7457.5(8) | 31.7(4) |
| C41 | 5257.9(11) | 4158.9(18) | 8122.5(8) | 35.2(4) |
| C40 | 4752.0(11) | 3060.9(17) | 7907.6(8) | 33.1(4) |
| C13 | 1143.1(12) | 9361.3(17) | 3560.3(8) | 35.3(4) |
| C12 | 1994.0(12) | 9934.8(17) | 3635.2(8) | 35.9(4) |
| C30 | -827.9(11) | 5695.7(19) | 6866.5(8) | 36.2(4) |
| C29 | -436.7(11) | 6859.9(18) | 6781.1(8) | 35.3(4) |
| B1 | 3137.0(11) | 6253.7(16) | 5933.2(8) | 21.3(3) |

| **Table 6-1-3** Anisotropic Displacement Parameters (Å^2^×10^3^) for dt01_auto. The Anisotropic displacement factor exponent takes the form: -2π^2^[h^2^a*^2^U_11_+2hka*b*U_12_+…]. | | | | | | |
| --- | --- | --- | --- | --- | --- | --- |
| **Atom** | **U_11_** | **U_22_** | **U_33_** | **U_23_** | **U_13_** | **U_12_** |
| O1 | 22.8(5) | 22.9(5) | 20.6(5) | -0.7(4) | 6.6(4) | -0.2(4) |
| O2 | 28.7(6) | 31.4(6) | 33.8(6) | -2.3(5) | 11.6(5) | -4.5(5) |
| N5 | 22.3(6) | 20.8(6) | 19.5(6) | 0.4(5) | 6.7(5) | 1.1(5) |
| N1 | 21.9(6) | 20.5(6) | 21.4(6) | -0.3(5) | 6.2(5) | 0.7(5) |
| N3 | 22.6(6) | 21.1(6) | 21.9(6) | 0.1(5) | 7.1(5) | 2.0(5) |
| N4 | 22.2(6) | 25.8(6) | 23.7(6) | -1.4(5) | 7.2(5) | 2.6(5) |
| N2 | 27.1(6) | 22.9(6) | 22.6(6) | -0.4(5) | 8.9(5) | -0.4(5) |
| N6 | 23.6(6) | 23.8(6) | 20.6(6) | 0.6(5) | 5.9(5) | 0.8(5) |
| C1 | 25.6(7) | 22.9(7) | 18.1(7) | 0.1(5) | 4.8(5) | 2.3(6) |
| C24 | 24.6(7) | 21.1(7) | 19.2(7) | 0.0(5) | 7.2(5) | 0.9(6) |
| C8 | 23.3(7) | 23.7(7) | 20.4(7) | -1.9(5) | 7.1(5) | -2.5(6) |
| C16 | 22.9(7) | 24.8(7) | 21.6(7) | -1.8(5) | 6.3(6) | 4.1(5) |
| C35 | 26.4(7) | 25.3(7) | 18.0(6) | 0.5(5) | 8.5(5) | 0.5(6) |
| C2 | 23.1(7) | 28.2(7) | 19.5(7) | -3.4(6) | 4.0(5) | 0.4(6) |
| C23 | 24.4(7) | 25.7(7) | 20.4(7) | -0.8(6) | 8.7(6) | -1.8(6) |
| C17 | 20.1(6) | 25.2(7) | 20.6(7) | -2.3(5) | 7.7(5) | 0.3(5) |
| C7 | 23.2(7) | 27.2(7) | 20.3(7) | -3.3(6) | 5.5(5) | -0.6(6) |
| C34 | 24.5(7) | 27.4(7) | 17.6(7) | -3.2(6) | 6.6(5) | 1.0(6) |
| C15 | 29.3(8) | 24.3(7) | 22.4(7) | -0.8(6) | 8.2(6) | 6.5(6) |
| C10 | 31.6(8) | 23.4(7) | 23.4(7) | -0.3(6) | 9.9(6) | 5.7(6) |
| C9 | 27.4(7) | 21.6(7) | 22.7(7) | 0.4(5) | 9.7(6) | 0.9(6) |
| C43 | 25.8(7) | 30.4(8) | 22.2(7) | 4.0(6) | 7.0(6) | 3.9(6) |
| C44 | 27.6(7) | 28.1(8) | 19.4(7) | 3.0(6) | 9.4(6) | 3.4(6) |
| C18 | 24.7(7) | 24.7(7) | 22.7(7) | -2.3(6) | 9.8(6) | -0.3(6) |
| C25 | 23.2(7) | 25.3(7) | 18.4(7) | -2.7(5) | 4.3(5) | 1.4(6) |
| C3 | 26.6(8) | 28.2(8) | 26.9(8) | -4.5(6) | 6.0(6) | 4.2(6) |
| C33 | 25.0(7) | 33.0(8) | 18.6(7) | -2.8(6) | 6.2(6) | 0.9(6) |
| C22 | 32.6(8) | 23.5(7) | 26.2(7) | -0.6(6) | 10.1(6) | 0.5(6) |
| C19 | 24.9(7) | 31.2(8) | 29.9(8) | -3.6(6) | 9.5(6) | -2.2(6) |
| C39 | 34.1(8) | 29.3(8) | 21.6(7) | 5.3(6) | 10.4(6) | 6.3(6) |
| C36 | 28.4(8) | 31.9(8) | 21.5(7) | -0.7(6) | 10.6(6) | -1.4(6) |
| C32 | 28.8(8) | 34.3(8) | 21.0(7) | -4.2(6) | 8.0(6) | -1.6(6) |
| C6 | 27.7(8) | 33.0(8) | 22.6(7) | -3.0(6) | 8.3(6) | -2.9(6) |
| C26 | 31.8(8) | 25.4(8) | 26.2(8) | -0.1(6) | 8.2(6) | 2.6(6) |
| C28 | 28.3(8) | 36.8(9) | 22.6(7) | -4.4(6) | 7.0(6) | 4.8(6) |
| C27 | 33.1(8) | 29.9(8) | 30.3(8) | -2.0(6) | 6.6(6) | 10.7(7) |
| C5 | 23.7(7) | 43.4(9) | 26.5(8) | -6.4(7) | 10.9(6) | -2.7(7) |
| C11 | 40.2(9) | 27.8(8) | 28.7(8) | 3.9(6) | 15.9(7) | 6.5(7) |
| C4 | 23.0(7) | 39.1(9) | 28.6(8) | -8.1(7) | 6.1(6) | 4.4(6) |
| C14 | 32.1(8) | 32.0(8) | 24.7(8) | -0.9(6) | 6.4(6) | 10.4(6) |
| C37 | 42.8(9) | 26.2(8) | 28.8(8) | 1.5(6) | 16.5(7) | -3.5(7) |
| C42 | 28.2(8) | 37.7(9) | 27.1(8) | 3.2(7) | 4.3(6) | 0.6(7) |
| C20 | 31.9(8) | 30.3(8) | 34.7(8) | -6.1(7) | 12.0(7) | -9.3(7) |
| C21 | 39.1(9) | 22.3(7) | 34.8(8) | -4.8(6) | 15.3(7) | -5.6(6) |
| C31 | 27.6(8) | 45.4(10) | 26.6(8) | -9.4(7) | 9.0(6) | -8.5(7) |
| C38 | 43.4(9) | 26.9(8) | 28.1(8) | 6.6(6) | 15.4(7) | 7.5(7) |
| C41 | 28.1(8) | 44.3(10) | 30.2(8) | 7.3(7) | 3.0(6) | 8.1(7) |
| C40 | 35.1(9) | 37.2(9) | 27.4(8) | 8.9(7) | 9.2(7) | 13.4(7) |
| C13 | 41.6(9) | 39.7(9) | 24.3(8) | 4.5(7) | 8.6(7) | 17.4(8) |
| C12 | 50.5(10) | 34.8(9) | 26.4(8) | 9.7(7) | 17.3(7) | 14.0(8) |
| C30 | 22.2(7) | 58.2(11) | 28.5(8) | -12.0(8) | 7.3(6) | -1.6(7) |
| C29 | 26.5(8) | 45.8(10) | 32.7(9) | -7.6(7) | 6.6(7) | 6.9(7) |
| B1 | 22.3(8) | 20.9(8) | 20.6(8) | 0.5(6) | 6.0(6) | 0.6(6) |

| **Table 6-1-4** Bond Lengths for **1b**. | | | | | | |
| --- | --- | --- | --- | --- | --- | --- |
| **Atom** | **Atom** | **Length/Å** |  | **Atom** | **Atom** | **Length/Å** |
| O1 | C25 | 1.3746(17) |  | C34 | C33 | 1.4250(19) |
| O1 | B1 | 1.4530(18) |  | C15 | C10 | 1.422(2) |
| O2 | C36 | 1.3803(18) |  | C15 | C14 | 1.398(2) |
| O2 | C32 | 1.3842(19) |  | C10 | C9 | 1.457(2) |
| N5 | C24 | 1.3627(18) |  | C10 | C11 | 1.396(2) |
| N5 | C17 | 1.3678(18) |  | C43 | C44 | 1.419(2) |
| N5 | B1 | 1.4933(19) |  | C43 | C42 | 1.375(2) |
| N1 | C1 | 1.3671(18) |  | C44 | C39 | 1.424(2) |
| N1 | C8 | 1.3614(18) |  | C18 | C19 | 1.390(2) |
| N1 | B1 | 1.4845(19) |  | C25 | C26 | 1.417(2) |
| N3 | C16 | 1.3682(18) |  | C3 | C4 | 1.382(2) |
| N3 | C9 | 1.3634(19) |  | C33 | C32 | 1.414(2) |
| N3 | B1 | 1.4961(19) |  | C33 | C28 | 1.424(2) |
| N4 | C16 | 1.3474(19) |  | C22 | C21 | 1.386(2) |
| N4 | C17 | 1.3443(19) |  | C19 | C20 | 1.382(2) |
| N2 | C8 | 1.3479(19) |  | C39 | C38 | 1.417(2) |
| N2 | C9 | 1.3450(19) |  | C39 | C40 | 1.420(2) |
| N6 | C1 | 1.3431(19) |  | C36 | C37 | 1.406(2) |
| N6 | C24 | 1.3462(18) |  | C32 | C31 | 1.369(2) |
| C1 | C2 | 1.4591(19) |  | C6 | C5 | 1.383(2) |
| C24 | C23 | 1.459(2) |  | C26 | C27 | 1.365(2) |
| C8 | C7 | 1.453(2) |  | C28 | C27 | 1.421(2) |
| C16 | C15 | 1.451(2) |  | C28 | C29 | 1.415(2) |
| C35 | C34 | 1.472(2) |  | C5 | C4 | 1.399(2) |
| C35 | C44 | 1.440(2) |  | C11 | C12 | 1.387(2) |
| C35 | C36 | 1.384(2) |  | C14 | C13 | 1.382(2) |
| C2 | C7 | 1.425(2) |  | C37 | C38 | 1.365(2) |
| C2 | C3 | 1.392(2) |  | C42 | C41 | 1.407(2) |
| C23 | C18 | 1.428(2) |  | C20 | C21 | 1.398(2) |
| C23 | C22 | 1.389(2) |  | C31 | C30 | 1.404(3) |
| C17 | C18 | 1.452(2) |  | C41 | C40 | 1.366(2) |
| C7 | C6 | 1.395(2) |  | C13 | C12 | 1.395(3) |
| C34 | C25 | 1.387(2) |  | C30 | C29 | 1.364(3) |

| **Table 6-1-5** Bond Angles for **1b**. | | | | | | | | |
| --- | --- | --- | --- | --- | --- | --- | --- | --- |
| **Atom** | **Atom** | **Atom** | **Angle/˚** |  | **Atom** | **Atom** | **Atom** | **Angle/˚** |
| C25 | O1 | B1 | 115.84(11) |  | C42 | C43 | C44 | 121.30(15) |
| C36 | O2 | C32 | 118.00(12) |  | C43 | C44 | C35 | 122.98(13) |
| C24 | N5 | C17 | 113.58(12) |  | C43 | C44 | C39 | 117.84(14) |
| C24 | N5 | B1 | 122.75(12) |  | C39 | C44 | C35 | 119.00(14) |
| C17 | N5 | B1 | 122.94(12) |  | C23 | C18 | C17 | 107.11(12) |
| C1 | N1 | B1 | 122.43(12) |  | C19 | C18 | C23 | 121.19(14) |
| C8 | N1 | C1 | 113.39(12) |  | C19 | C18 | C17 | 131.52(14) |
| C8 | N1 | B1 | 123.24(12) |  | O1 | C25 | C34 | 121.60(12) |
| C16 | N3 | B1 | 122.73(12) |  | O1 | C25 | C26 | 118.36(13) |
| C9 | N3 | C16 | 113.37(12) |  | C34 | C25 | C26 | 120.03(13) |
| C9 | N3 | B1 | 122.78(12) |  | C4 | C3 | C2 | 117.84(15) |
| C17 | N4 | C16 | 116.89(12) |  | C32 | C33 | C34 | 119.85(14) |
| C9 | N2 | C8 | 117.04(12) |  | C32 | C33 | C28 | 118.47(14) |
| C1 | N6 | C24 | 116.51(12) |  | C28 | C33 | C34 | 121.57(14) |
| N1 | C1 | C2 | 105.20(12) |  | C21 | C22 | C23 | 117.96(15) |
| N6 | C1 | N1 | 122.86(13) |  | C20 | C19 | C18 | 117.48(15) |
| N6 | C1 | C2 | 130.53(13) |  | C38 | C39 | C44 | 119.47(14) |
| N5 | C24 | C23 | 105.44(12) |  | C38 | C39 | C40 | 121.36(14) |
| N6 | C24 | N5 | 122.64(13) |  | C40 | C39 | C44 | 119.12(15) |
| N6 | C24 | C23 | 130.69(13) |  | O2 | C36 | C35 | 122.37(14) |
| N1 | C8 | C7 | 105.53(12) |  | O2 | C36 | C37 | 114.96(14) |
| N2 | C8 | N1 | 122.35(13) |  | C35 | C36 | C37 | 122.66(14) |
| N2 | C8 | C7 | 130.94(13) |  | O2 | C32 | C33 | 119.81(13) |
| N3 | C16 | C15 | 105.67(12) |  | C31 | C32 | O2 | 118.38(14) |
| N4 | C16 | N3 | 122.60(13) |  | C31 | C32 | C33 | 121.70(15) |
| N4 | C16 | C15 | 129.98(13) |  | C5 | C6 | C7 | 117.77(15) |
| C44 | C35 | C34 | 124.86(13) |  | C27 | C26 | C25 | 121.34(14) |
| C36 | C35 | C34 | 117.71(13) |  | C27 | C28 | C33 | 117.21(14) |
| C36 | C35 | C44 | 117.22(14) |  | C29 | C28 | C33 | 118.49(15) |
| C7 | C2 | C1 | 106.97(12) |  | C29 | C28 | C27 | 124.27(15) |
| C3 | C2 | C1 | 132.35(14) |  | C26 | C27 | C28 | 120.62(14) |
| C3 | C2 | C7 | 120.64(14) |  | C6 | C5 | C4 | 121.50(14) |
| C18 | C23 | C24 | 107.13(12) |  | C12 | C11 | C10 | 117.60(15) |
| C22 | C23 | C24 | 132.56(14) |  | C3 | C4 | C5 | 121.56(15) |
| C22 | C23 | C18 | 120.20(14) |  | C13 | C14 | C15 | 117.43(15) |
| N5 | C17 | C18 | 105.60(12) |  | C38 | C37 | C36 | 119.30(15) |
| N4 | C17 | N5 | 122.45(13) |  | C43 | C42 | C41 | 120.18(16) |
| N4 | C17 | C18 | 130.29(13) |  | C19 | C20 | C21 | 121.64(15) |
| C2 | C7 | C8 | 107.20(12) |  | C22 | C21 | C20 | 121.47(15) |
| C6 | C7 | C8 | 132.16(14) |  | C32 | C31 | C30 | 118.83(16) |
| C6 | C7 | C2 | 120.61(14) |  | C37 | C38 | C39 | 120.78(14) |
| C25 | C34 | C35 | 126.74(13) |  | C40 | C41 | C42 | 119.99(15) |
| C25 | C34 | C33 | 117.56(13) |  | C41 | C40 | C39 | 121.02(15) |
| C33 | C34 | C35 | 115.65(13) |  | C14 | C13 | C12 | 121.87(15) |
| C10 | C15 | C16 | 106.98(12) |  | C11 | C12 | C13 | 121.59(15) |
| C14 | C15 | C16 | 131.98(14) |  | C29 | C30 | C31 | 121.46(14) |
| C14 | C15 | C10 | 120.93(14) |  | C30 | C29 | C28 | 120.61(16) |
| C15 | C10 | C9 | 107.44(13) |  | O1 | B1 | N5 | 116.36(12) |
| C11 | C10 | C15 | 120.56(14) |  | O1 | B1 | N1 | 112.03(12) |
| C11 | C10 | C9 | 131.88(14) |  | O1 | B1 | N3 | 114.98(12) |
| N3 | C9 | C10 | 105.24(13) |  | N5 | B1 | N3 | 103.71(11) |
| N2 | C9 | N3 | 122.57(13) |  | N1 | B1 | N5 | 104.08(11) |
| N2 | C9 | C10 | 130.66(13) |  | N1 | B1 | N3 | 104.36(11) |

| **Table 6-1-6** Torsion Angles for **1b**. | | | | | | | | | | |
| --- | --- | --- | --- | --- | --- | --- | --- | --- | --- | --- |
| **A** | **B** | **C** | **D** | **Angle/˚** |  | **A** | **B** | **C** | **D** | **Angle/˚** |
| O1 | C25 | C26 | C27 | 174.63(13) |  | C34 | C33 | C32 | C31 | 176.55(14) |
| O2 | C36 | C37 | C38 | -175.94(14) |  | C34 | C33 | C28 | C27 | 5.2(2) |
| O2 | C32 | C31 | C30 | -173.86(13) |  | C34 | C33 | C28 | C29 | -176.82(14) |
| N5 | C24 | C23 | C18 | 5.71(14) |  | C15 | C10 | C9 | N3 | -6.25(15) |
| N5 | C24 | C23 | C22 | -178.17(15) |  | C15 | C10 | C9 | N2 | 159.57(15) |
| N5 | C17 | C18 | C23 | -6.68(15) |  | C15 | C10 | C11 | C12 | 1.6(2) |
| N5 | C17 | C18 | C19 | 178.33(15) |  | C15 | C14 | C13 | C12 | 0.5(2) |
| N1 | C1 | C2 | C7 | -7.91(15) |  | C10 | C15 | C14 | C13 | -0.6(2) |
| N1 | C1 | C2 | C3 | 174.45(15) |  | C10 | C11 | C12 | C13 | -1.6(2) |
| N1 | C8 | C7 | C2 | 7.24(15) |  | C9 | N3 | C16 | N4 | 154.54(13) |
| N1 | C8 | C7 | C6 | -174.97(15) |  | C9 | N3 | C16 | C15 | -11.75(16) |
| N3 | C16 | C15 | C10 | 7.00(15) |  | C9 | N3 | B1 | O1 | 94.87(16) |
| N3 | C16 | C15 | C14 | -176.98(15) |  | C9 | N3 | B1 | N5 | -136.97(13) |
| N4 | C16 | C15 | C10 | -157.90(14) |  | C9 | N3 | B1 | N1 | -28.25(17) |
| N4 | C16 | C15 | C14 | 18.1(3) |  | C9 | N2 | C8 | N1 | -7.7(2) |
| N4 | C17 | C18 | C23 | 158.57(14) |  | C9 | N2 | C8 | C7 | 157.99(14) |
| N4 | C17 | C18 | C19 | -16.4(3) |  | C9 | C10 | C11 | C12 | 177.09(15) |
| N2 | C8 | C7 | C2 | -160.28(15) |  | C43 | C44 | C39 | C38 | 169.53(13) |
| N2 | C8 | C7 | C6 | 17.5(3) |  | C43 | C44 | C39 | C40 | -7.9(2) |
| N6 | C1 | C2 | C7 | 158.50(14) |  | C43 | C42 | C41 | C40 | -5.3(2) |
| N6 | C1 | C2 | C3 | -19.1(3) |  | C44 | C35 | C34 | C25 | 31.3(2) |
| N6 | C24 | C23 | C18 | -161.55(14) |  | C44 | C35 | C34 | C33 | -151.33(14) |
| N6 | C24 | C23 | C22 | 14.6(3) |  | C44 | C35 | C36 | O2 | 166.75(13) |
| C1 | N1 | C8 | N2 | 155.71(13) |  | C44 | C35 | C36 | C37 | -13.2(2) |
| C1 | N1 | C8 | C7 | -13.15(16) |  | C44 | C43 | C42 | C41 | 2.6(2) |
| C1 | N1 | B1 | O1 | 95.63(15) |  | C44 | C39 | C38 | C37 | -3.7(2) |
| C1 | N1 | B1 | N5 | -30.91(17) |  | C44 | C39 | C40 | C41 | 5.4(2) |
| C1 | N1 | B1 | N3 | -139.35(13) |  | C18 | C23 | C22 | C21 | -1.7(2) |
| C1 | N6 | C24 | N5 | -8.93(19) |  | C18 | C19 | C20 | C21 | -1.4(2) |
| C1 | N6 | C24 | C23 | 156.45(14) |  | C25 | O1 | B1 | N5 | -46.75(17) |
| C1 | C2 | C7 | C8 | 0.44(15) |  | C25 | O1 | B1 | N1 | -166.31(11) |
| C1 | C2 | C7 | C6 | -177.66(13) |  | C25 | O1 | B1 | N3 | 74.76(15) |
| C1 | C2 | C3 | C4 | 179.68(15) |  | C25 | C34 | C33 | C32 | 161.90(14) |
| C24 | N5 | C17 | N4 | -155.64(13) |  | C25 | C34 | C33 | C28 | -14.2(2) |
| C24 | N5 | C17 | C18 | 11.06(15) |  | C25 | C26 | C27 | C28 | -5.3(2) |
| C24 | N5 | B1 | O1 | -93.87(15) |  | C3 | C2 | C7 | C8 | 178.41(13) |
| C24 | N5 | B1 | N1 | 29.90(16) |  | C3 | C2 | C7 | C6 | 0.3(2) |
| C24 | N5 | B1 | N3 | 138.83(12) |  | C33 | C34 | C25 | O1 | -165.22(12) |
| C24 | N6 | C1 | N1 | 7.8(2) |  | C33 | C34 | C25 | C26 | 13.5(2) |
| C24 | N6 | C1 | C2 | -156.50(14) |  | C33 | C32 | C31 | C30 | 2.4(2) |
| C24 | C23 | C18 | C17 | 0.61(15) |  | C33 | C28 | C27 | C26 | 4.7(2) |
| C24 | C23 | C18 | C19 | 176.22(13) |  | C33 | C28 | C29 | C30 | -2.2(2) |
| C24 | C23 | C22 | C21 | -177.42(14) |  | C22 | C23 | C18 | C17 | -176.08(13) |
| C8 | N1 | C1 | N6 | -154.33(13) |  | C22 | C23 | C18 | C19 | -0.5(2) |
| C8 | N1 | C1 | C2 | 13.39(16) |  | C19 | C20 | C21 | C22 | -0.8(2) |
| C8 | N1 | B1 | O1 | -96.20(15) |  | C36 | O2 | C32 | C33 | 23.2(2) |
| C8 | N1 | B1 | N5 | 137.27(13) |  | C36 | O2 | C32 | C31 | -160.53(13) |
| C8 | N1 | B1 | N3 | 28.82(17) |  | C36 | C35 | C34 | C25 | -154.07(14) |
| C8 | N2 | C9 | N3 | 8.2(2) |  | C36 | C35 | C34 | C33 | 23.26(18) |
| C8 | N2 | C9 | C10 | -155.56(15) |  | C36 | C35 | C44 | C43 | -161.25(13) |
| C8 | C7 | C6 | C5 | 179.90(15) |  | C36 | C35 | C44 | C39 | 13.81(19) |
| C16 | N3 | C9 | N2 | -155.86(13) |  | C36 | C37 | C38 | C39 | 4.7(2) |
| C16 | N3 | C9 | C10 | 11.40(16) |  | C32 | O2 | C36 | C35 | -15.2(2) |
| C16 | N3 | B1 | O1 | -98.00(16) |  | C32 | O2 | C36 | C37 | 164.73(13) |
| C16 | N3 | B1 | N5 | 30.16(17) |  | C32 | C33 | C28 | C27 | -170.99(14) |
| C16 | N3 | B1 | N1 | 138.87(13) |  | C32 | C33 | C28 | C29 | 7.0(2) |
| C16 | N4 | C17 | N5 | 7.40(19) |  | C32 | C31 | C30 | C29 | 2.8(2) |
| C16 | N4 | C17 | C18 | -155.71(14) |  | C6 | C5 | C4 | C3 | 0.5(2) |
| C16 | C15 | C10 | C9 | -0.47(16) |  | C28 | C33 | C32 | O2 | 168.94(13) |
| C16 | C15 | C10 | C11 | 176.05(13) |  | C28 | C33 | C32 | C31 | -7.2(2) |
| C16 | C15 | C14 | C13 | -176.12(15) |  | C27 | C28 | C29 | C30 | 175.63(16) |
| C35 | C34 | C25 | O1 | 12.1(2) |  | C11 | C10 | C9 | N3 | 177.77(15) |
| C35 | C34 | C25 | C26 | -169.17(14) |  | C11 | C10 | C9 | N2 | -16.4(3) |
| C35 | C34 | C33 | C32 | -15.69(19) |  | C14 | C15 | C10 | C9 | -177.02(13) |
| C35 | C34 | C33 | C28 | 168.21(13) |  | C14 | C15 | C10 | C11 | -0.5(2) |
| C35 | C44 | C39 | C38 | -5.8(2) |  | C14 | C13 | C12 | C11 | 0.5(3) |
| C35 | C44 | C39 | C40 | 176.80(13) |  | C42 | C43 | C44 | C35 | 179.11(13) |
| C35 | C36 | C37 | C38 | 4.0(2) |  | C42 | C43 | C44 | C39 | 4.0(2) |
| C2 | C7 | C6 | C5 | -2.6(2) |  | C42 | C41 | C40 | C39 | 1.3(2) |
| C2 | C3 | C4 | C5 | -2.7(2) |  | C31 | C30 | C29 | C28 | -2.8(3) |
| C23 | C18 | C19 | C20 | 2.0(2) |  | C38 | C39 | C40 | C41 | -171.96(15) |
| C23 | C22 | C21 | C20 | 2.3(2) |  | C40 | C39 | C38 | C37 | 173.70(15) |
| C17 | N5 | C24 | N6 | 157.89(13) |  | C29 | C28 | C27 | C26 | -173.22(15) |
| C17 | N5 | C24 | C23 | -10.65(15) |  | B1 | O1 | C25 | C34 | 80.76(16) |
| C17 | N5 | B1 | O1 | 96.54(15) |  | B1 | O1 | C25 | C26 | -98.02(15) |
| C17 | N5 | B1 | N1 | -139.69(12) |  | B1 | N5 | C24 | N6 | -12.6(2) |
| C17 | N5 | B1 | N3 | -30.76(16) |  | B1 | N5 | C24 | C23 | 178.87(12) |
| C17 | N4 | C16 | N3 | -7.9(2) |  | B1 | N5 | C17 | N4 | 14.8(2) |
| C17 | N4 | C16 | C15 | 154.76(14) |  | B1 | N5 | C17 | C18 | -178.49(12) |
| C17 | C18 | C19 | C20 | 176.42(15) |  | B1 | N1 | C1 | N6 | 14.9(2) |
| C7 | C2 | C3 | C4 | 2.3(2) |  | B1 | N1 | C1 | C2 | -177.37(12) |
| C7 | C6 | C5 | C4 | 2.2(2) |  | B1 | N1 | C8 | N2 | -13.4(2) |
| C34 | C35 | C44 | C43 | 13.4(2) |  | B1 | N1 | C8 | C7 | 177.71(12) |
| C34 | C35 | C44 | C39 | -171.58(13) |  | B1 | N3 | C16 | N4 | -13.7(2) |
| C34 | C35 | C36 | O2 | -8.3(2) |  | B1 | N3 | C16 | C15 | -179.98(12) |
| C34 | C35 | C36 | C37 | 171.78(13) |  | B1 | N3 | C9 | N2 | 12.4(2) |
| C34 | C25 | C26 | C27 | -4.2(2) |  | B1 | N3 | C9 | C10 | 179.62(12) |
| C34 | C33 | C32 | O2 | -7.3(2) |  |  |  |  |  |  |

| **Table 6-1-7** Hydrogen Atom Coordinates (Å×10^4^) and Isotropic Displacement Parameters (Å^2^×10^3^) for **1b**. | | | | |
| --- | --- | --- | --- | --- |
| **Atom** | ***x*** | ***y*** | ***z*** | **U(eq)** |
| H43 | 3660.63 | 6346.89 | 7681.08 | 31 |
| H3 | 6088.46 | 3214.17 | 6240.76 | 33 |
| H22 | 2884.32 | 995.34 | 5819 | 32 |
| H19 | 223.57 | 3326.5 | 4774.73 | 34 |
| H6 | 6279.23 | 7449.37 | 5415.33 | 33 |
| H26 | 2047.85 | 8754.69 | 6456.44 | 33 |
| H27 | 627.07 | 8866.41 | 6602.92 | 38 |
| H5 | 7527.01 | 6057.54 | 5670.76 | 36 |
| H11 | 3319.1 | 9923.28 | 4169.39 | 37 |
| H4 | 7435.37 | 3985.1 | 6082.24 | 37 |
| H14 | 438.2 | 8010.65 | 3919.5 | 36 |
| H37 | 1967.58 | 1352.92 | 7159.62 | 38 |
| H42 | 5170.21 | 6133.74 | 8238.14 | 38 |
| H20 | 233.35 | 1053.5 | 4811.85 | 38 |
| H21 | 1532.22 | -89.68 | 5333.92 | 37 |
| H31 | -630.6 | 3716.55 | 6959.23 | 39 |
| H38 | 3523.7 | 1183.09 | 7517.07 | 38 |
| H41 | 5882.18 | 4088.68 | 8310.32 | 42 |
| H40 | 5034.61 | 2244.42 | 7958.6 | 40 |
| H13 | 653.33 | 9638.82 | 3221.43 | 42 |
| H12 | 2056.01 | 10589.26 | 3347.63 | 43 |
| H30 | -1426 | 5688.48 | 6890.1 | 43 |
| H29 | -778.28 | 7627.52 | 6728.59 | 42 |

6-2 **1c**

**Figure SI-20** Molecular structure of **1c**. Thermal ellipsoids are shown at the 50% probability level.

| **Table 6-2-1** Crystal data and structure refinement for **1c**. | |
| --- | --- |
| Identification code | **1c** |
| Empirical formula | C_45_H_27_BN_6_O_2_ |
| Formula weight | 694.53 |
| Temperature/K | 100.15 |
| Crystal system | monoclinic |
| Space group | P2_1_/n |
| a/Å | 12.8291(3) |
| b/Å | 12.2202(2) |
| c/Å | 21.2801(4) |
| α/° | 90 |
| β/° | 97.477(2) |
| γ/° | 90 |
| Volume/Å^3^ | 3307.80(11) |
| Z | 4 |
| ρ_calc_g/cm^3^ | 1.395 |
| μ/mm^‑1^ | 0.695 |
| F(000) | 1440.0 |
| Crystal size/mm^3^ | 0.19 × 0.126 × 0.097 |
| Radiation | Cu Kα (λ = 1.54184) |
| 2Θ range for data collection/° | 7.634 to 142.66 |
| Index ranges | -15 ≤ h ≤ 15, -14 ≤ k ≤ 4, -25 ≤ l ≤ 26 |
| Reflections collected | 17304 |
| Independent reflections | 6227 [R_int_ = 0.0286, R_sigma_ = 0.0327] |
| Data/restraints/parameters | 6227/0/488 |
| Goodness-of-fit on F^2^ | 1.042 |
| Final R indexes [I>=2σ (I)] | R_1_ = 0.0670, wR_2_ = 0.1852 |
| Final R indexes [all data] | R_1_ = 0.0743, wR_2_ = 0.1919 |
| Largest diff. peak/hole / e Å^-3^ | 1.31/-0.48 |

| **Table 6-2-2** Fractional Atomic Coordinates (×10^4^) and Equivalent Isotropic Displacement Parameters (Å^2^×10^3^) for dt13_auto. U_eq_ is defined as 1/3 of the trace of the orthogonalised U_IJ_ tensor. | | | | |
| --- | --- | --- | --- | --- |
| **Atom** | ***x*** | ***y*** | ***z*** | **U(eq)** |
| O1 | 6198.5(14) | 4062.6(14) | 2667.8(8) | 29.0(4) |
| N1 | 5909.5(16) | 2833.7(17) | 3492.8(9) | 25.9(4) |
| O2 | 3249.5(16) | 4987.5(18) | 1575.8(10) | 42.0(5) |
| N5 | 4425.5(16) | 3819.3(17) | 3021.5(9) | 26.0(4) |
| N3 | 5730.3(16) | 4701.3(17) | 3727.1(9) | 26.5(4) |
| N6 | 4429.0(16) | 1871.5(17) | 2981.6(9) | 27.0(4) |
| N4 | 4029.3(17) | 5502.1(17) | 3502.5(10) | 28.7(5) |
| N2 | 6966.2(16) | 3602.0(18) | 4381.8(10) | 29.1(5) |
| C23 | 2819.6(19) | 3074(2) | 2777.5(11) | 27.6(5) |
| C1 | 5394.7(19) | 1884(2) | 3322.3(11) | 26.1(5) |
| C24 | 3942.6(19) | 2837(2) | 2879.0(11) | 26.3(5) |
| C16 | 5006(2) | 5489(2) | 3821.2(11) | 27.6(5) |
| C7 | 6734.4(19) | 1566(2) | 4141.4(11) | 28.5(5) |
| C18 | 2692(2) | 4190(2) | 2946.1(11) | 28.2(5) |
| C17 | 3742(2) | 4626(2) | 3140.6(11) | 26.9(5) |
| C8 | 6636.6(18) | 2736(2) | 4018.7(11) | 26.3(5) |
| C2 | 5954.3(19) | 1036(2) | 3709.5(12) | 28.0(5) |
| C10 | 6343(2) | 5483(2) | 4661.1(12) | 30.0(5) |
| C25 | 6086(2) | 4983(2) | 2294.1(11) | 28.7(5) |
| C9 | 6464.1(19) | 4553(2) | 4251.7(11) | 28.1(5) |
| C22 | 1946(2) | 2411(2) | 2611.0(12) | 31.1(5) |
| C15 | 5425(2) | 6061(2) | 4398.6(12) | 29.9(5) |
| C34 | 5441(2) | 4977(2) | 1718.3(12) | 31.6(6) |
| C33 | 5394(2) | 5948(2) | 1337.8(12) | 33.0(6) |
| C26 | 6697(2) | 5902(2) | 2501.3(12) | 31.9(6) |
| C28 | 5984(2) | 6892(2) | 1564.8(12) | 33.1(6) |
| C20 | 838(2) | 3959(2) | 2783.9(12) | 35.6(6) |
| C35 | 4798(2) | 3983(2) | 1496.8(12) | 36.5(6) |
| C27 | 6646(2) | 6840(2) | 2150.6(13) | 33.5(6) |
| C6 | 7378(2) | 962(2) | 4592.6(13) | 33.5(6) |
| C11 | 6922(2) | 5833(2) | 5226.3(12) | 36.7(6) |
| C3 | 5808(2) | -90(2) | 3738.5(13) | 33.3(6) |
| C19 | 1695(2) | 4629(2) | 2953.4(12) | 32.0(6) |
| C21 | 958(2) | 2864(2) | 2608.6(13) | 35.6(6) |
| C14 | 5064(2) | 6960(2) | 4711.3(13) | 36.1(6) |
| C37 | 6400(3) | 2937(2) | 1365.0(13) | 41.0(7) |
| C5 | 7239(2) | -159(2) | 4606.2(13) | 37.2(6) |
| C12 | 6576(3) | 6772(2) | 5512.6(12) | 43.0(7) |
| C4 | 6461(2) | -676(2) | 4187.7(14) | 38.4(6) |
| C29 | 5890(2) | 7861(2) | 1198.2(14) | 38.3(6) |
| C13 | 5667(3) | 7312(2) | 5264.7(13) | 40.9(7) |
| C32 | 4785(2) | 6007(2) | 729.8(13) | 40.1(7) |
| C44 | 3710(2) | 4022(2) | 1428.7(12) | 39.5(7) |
| C36 | 5297(3) | 3001(2) | 1344.0(12) | 39.2(7) |
| C30 | 5289(3) | 7897(2) | 626.5(14) | 44.0(7) |
| C41 | 4635(3) | 2061(2) | 1134.0(13) | 45.9(8) |
| C31 | 4731(3) | 6953(3) | 385.1(14) | 46.0(7) |
| C43 | 3073(3) | 3093(3) | 1223.9(14) | 45.1(7) |
| C45 | 2153(2) | 5111(4) | 1382.7(14) | 53.7(9) |
| C42 | 3534(3) | 2170(3) | 1088.3(14) | 48.2(8) |
| B1 | 5601(2) | 3899(2) | 3184.5(13) | 26.8(6) |
| C40 | 5117(3) | 1107(3) | 967.3(15) | 49.8(8) |
| C38 | 6829(3) | 1995(3) | 1158.5(15) | 49.0(8) |
| C39 | 6183(3) | 1044(3) | 967.6(16) | 48.0(8) |

| **Table 6-2-3** Anisotropic Displacement Parameters (Å^2^×10^3^) for dt13_auto. The Anisotropic displacement factor exponent takes the form: -2π^2^[h^2^a*^2^U_11_+2hka*b*U_12_+…]. | | | | | | |
| --- | --- | --- | --- | --- | --- | --- |
| **Atom** | **U_11_** | **U_22_** | **U_33_** | **U_23_** | **U_13_** | **U_12_** |
| O1 | 34.2(9) | 32.7(9) | 20.9(8) | 3.4(7) | 6.5(7) | -1.0(7) |
| N1 | 27.5(10) | 30.1(11) | 20.3(10) | 1.8(8) | 3.8(8) | -2.0(8) |
| O2 | 36.6(10) | 51.3(12) | 37.6(11) | 3.6(9) | 3.2(8) | 0.9(9) |
| N5 | 30.1(10) | 28.8(10) | 19.3(9) | 2.2(8) | 4.5(8) | -1.9(8) |
| N3 | 31.9(11) | 28.3(10) | 19.3(9) | 2.2(8) | 3.8(8) | -5.2(8) |
| N6 | 30.1(10) | 31.7(11) | 19.1(9) | -0.3(8) | 2.4(8) | -1.5(8) |
| N4 | 34.9(11) | 27.9(11) | 23.9(10) | 4.5(8) | 5.9(8) | -1.8(9) |
| N2 | 31.0(11) | 34.3(11) | 21.9(10) | 1.6(8) | 3.7(8) | -6.4(9) |
| C23 | 30.7(12) | 35.9(13) | 16.1(11) | 2.7(9) | 2.4(9) | -0.9(10) |
| C1 | 31.1(12) | 28.9(12) | 19.1(11) | -0.4(9) | 5.5(9) | -3.7(10) |
| C24 | 30.9(12) | 31.0(12) | 16.8(11) | 0.0(9) | 2.5(9) | -3.3(10) |
| C16 | 34.8(13) | 26.6(12) | 22.6(11) | 3.6(9) | 8.3(10) | -5.1(10) |
| C7 | 28.6(12) | 34.1(13) | 23.2(12) | 2.3(10) | 4.9(9) | -1.6(10) |
| C18 | 32.6(13) | 32.6(13) | 19.1(11) | 4.9(9) | 2.7(9) | -1.3(10) |
| C17 | 33.3(13) | 27.5(12) | 20.3(11) | 5.8(9) | 5.0(9) | 0.3(10) |
| C8 | 25.1(11) | 34.6(13) | 19.3(11) | 2.9(9) | 3.7(9) | -3.2(10) |
| C2 | 30.0(12) | 30.6(13) | 24.2(12) | 0.6(10) | 6.9(10) | -0.2(10) |
| C10 | 38.3(14) | 29.7(13) | 23.1(12) | 2.0(10) | 8.7(10) | -11.9(10) |
| C25 | 32.7(13) | 32.5(13) | 22.8(12) | 1.8(10) | 11.3(10) | -2.2(10) |
| C9 | 31.3(12) | 32.1(13) | 21.4(11) | 2.6(10) | 5.0(9) | -8.8(10) |
| C22 | 33.4(13) | 37.5(14) | 22.3(12) | -0.8(10) | 3.2(10) | -4.4(11) |
| C15 | 40.1(14) | 27.9(12) | 23.5(12) | 2.3(10) | 10.5(10) | -9.0(10) |
| C34 | 42.1(14) | 32.9(13) | 20.8(12) | -0.7(10) | 8.0(10) | -3.7(11) |
| C33 | 44.1(15) | 34.1(14) | 22.4(12) | 0.9(10) | 10.0(11) | -3.3(11) |
| C26 | 32.4(13) | 39.6(15) | 24.3(12) | -0.9(11) | 5.7(10) | -5.9(11) |
| C28 | 40.2(14) | 33.6(14) | 28.3(13) | 1.2(10) | 15.2(11) | -1.9(11) |
| C20 | 29.8(13) | 50.3(16) | 27.1(13) | 4.4(11) | 5.1(10) | 3.6(11) |
| C35 | 59.8(18) | 33.5(14) | 15.9(11) | 2.4(10) | 3.8(11) | -7.4(12) |
| C27 | 37.2(14) | 32.8(14) | 32.8(13) | -3.6(11) | 13.6(11) | -6.8(11) |
| C6 | 32.9(13) | 39.0(15) | 27.9(13) | 4.8(11) | 1.1(10) | -0.5(11) |
| C11 | 50.9(16) | 38.8(15) | 20.4(12) | 2.9(11) | 4.6(11) | -13.9(12) |
| C3 | 33.6(13) | 32.1(14) | 34.3(14) | 2.0(11) | 5.0(11) | -2.1(10) |
| C19 | 37.3(14) | 37.0(14) | 22.1(12) | 3.8(10) | 5.9(10) | 3.4(11) |
| C21 | 31.3(13) | 47.2(16) | 27.7(13) | 0.6(11) | 1.9(10) | -6.6(11) |
| C14 | 48.1(16) | 31.7(13) | 31.1(14) | -0.3(11) | 15.3(12) | -7.8(12) |
| C37 | 70(2) | 32.4(14) | 24.6(13) | 5.7(11) | 19.5(13) | 7.8(13) |
| C5 | 39.0(14) | 40.4(15) | 31.7(14) | 9.4(12) | 2.1(11) | 2.6(12) |
| C12 | 69(2) | 41.9(16) | 19.0(12) | -3.3(11) | 8.5(12) | -21.6(15) |
| C4 | 42.1(15) | 32.5(14) | 40.3(15) | 7.4(12) | 4.8(12) | -1.5(12) |
| C29 | 47.4(16) | 33.3(14) | 37.9(15) | 3.4(12) | 19.3(13) | -1.6(12) |
| C13 | 62.4(19) | 34.4(14) | 29.8(14) | -4.8(11) | 21.1(13) | -10.6(13) |
| C32 | 51.8(17) | 42.8(16) | 25.9(13) | 4.1(12) | 5.5(12) | -5.6(13) |
| C44 | 49.1(16) | 47.7(17) | 19.8(12) | 4.0(11) | -2.7(11) | -14.7(13) |
| C36 | 62.1(19) | 35.6(15) | 20.7(12) | 2.6(11) | 9.2(12) | -4.7(13) |
| C30 | 59.4(19) | 38.6(15) | 36.7(15) | 12.6(12) | 16.8(14) | 2.3(13) |
| C41 | 87(2) | 31.0(14) | 20.7(13) | 3.2(11) | 11.9(14) | 1.3(14) |
| C31 | 58.2(19) | 52.1(18) | 27.6(14) | 10.2(13) | 4.7(13) | -3.2(15) |
| C43 | 54.6(18) | 48.9(18) | 29.8(14) | 5.1(13) | -1.7(13) | -10.0(14) |
| C45 | 36.3(16) | 94(3) | 29.3(15) | -4.0(16) | 0.3(12) | -4.6(16) |
| C42 | 54.8(19) | 60(2) | 27.7(14) | 3.7(13) | -2.8(13) | -16.0(16) |
| B1 | 29.2(14) | 30.1(14) | 20.9(13) | 2.0(11) | 2.1(10) | -3.0(11) |
| C40 | 63(2) | 44.8(17) | 42.3(17) | 0.8(14) | 10.0(15) | -2.1(15) |
| C38 | 62(2) | 49.1(18) | 36.3(16) | 5.6(13) | 9.3(14) | 17.2(15) |
| C39 | 59(2) | 41.7(17) | 44.0(17) | 4.1(13) | 11.0(15) | 8.8(14) |

| **Table 6-2-4** Bond Lengths for **1c**. | | | | | | |
| --- | --- | --- | --- | --- | --- | --- |
| **Atom** | **Atom** | **Length/Å** |  | **Atom** | **Atom** | **Length/Å** |
| O1 | C25 | 1.374(3) |  | C25 | C34 | 1.386(4) |
| O1 | B1 | 1.434(3) |  | C25 | C26 | 1.408(4) |
| N1 | C1 | 1.361(3) |  | C22 | C21 | 1.383(4) |
| N1 | C8 | 1.366(3) |  | C15 | C14 | 1.394(4) |
| N1 | B1 | 1.488(3) |  | C34 | C33 | 1.433(4) |
| O2 | C44 | 1.374(4) |  | C34 | C35 | 1.509(4) |
| O2 | C45 | 1.422(4) |  | C33 | C28 | 1.429(4) |
| N5 | C24 | 1.367(3) |  | C33 | C32 | 1.424(4) |
| N5 | C17 | 1.365(3) |  | C26 | C27 | 1.364(4) |
| N5 | B1 | 1.506(3) |  | C28 | C27 | 1.416(4) |
| N3 | C16 | 1.370(3) |  | C28 | C29 | 1.415(4) |
| N3 | C9 | 1.375(3) |  | C20 | C19 | 1.380(4) |
| N3 | B1 | 1.507(3) |  | C20 | C21 | 1.402(4) |
| N6 | C1 | 1.352(3) |  | C35 | C44 | 1.384(4) |
| N6 | C24 | 1.339(3) |  | C35 | C36 | 1.418(4) |
| N4 | C16 | 1.345(3) |  | C6 | C5 | 1.382(4) |
| N4 | C17 | 1.342(3) |  | C11 | C12 | 1.398(4) |
| N2 | C8 | 1.345(3) |  | C3 | C4 | 1.385(4) |
| N2 | C9 | 1.340(3) |  | C14 | C13 | 1.390(4) |
| C23 | C24 | 1.458(3) |  | C37 | C36 | 1.412(5) |
| C23 | C18 | 1.425(4) |  | C37 | C38 | 1.373(4) |
| C23 | C22 | 1.391(4) |  | C5 | C4 | 1.399(4) |
| C1 | C2 | 1.454(3) |  | C12 | C13 | 1.384(5) |
| C16 | C15 | 1.454(3) |  | C29 | C30 | 1.354(4) |
| C7 | C8 | 1.456(4) |  | C32 | C31 | 1.367(4) |
| C7 | C2 | 1.423(3) |  | C44 | C43 | 1.433(4) |
| C7 | C6 | 1.394(4) |  | C36 | C41 | 1.463(4) |
| C18 | C17 | 1.458(3) |  | C30 | C31 | 1.417(5) |
| C18 | C19 | 1.389(4) |  | C41 | C42 | 1.410(5) |
| C2 | C3 | 1.392(4) |  | C41 | C40 | 1.387(5) |
| C10 | C9 | 1.452(4) |  | C43 | C42 | 1.323(5) |
| C10 | C15 | 1.425(4) |  | C40 | C39 | 1.370(5) |
| C10 | C11 | 1.396(4) |  | C38 | C39 | 1.453(5) |

| **Table 6-2-5** Bond Angles for **1c**. | | | | | | | | |
| --- | --- | --- | --- | --- | --- | --- | --- | --- |
| **Atom** | **Atom** | **Atom** | **Angle/˚** |  | **Atom** | **Atom** | **Atom** | **Angle/˚** |
| C25 | O1 | B1 | 122.1(2) |  | C14 | C15 | C16 | 132.3(3) |
| C1 | N1 | C8 | 113.6(2) |  | C14 | C15 | C10 | 120.8(2) |
| C1 | N1 | B1 | 122.5(2) |  | C25 | C34 | C33 | 118.1(2) |
| C8 | N1 | B1 | 123.5(2) |  | C25 | C34 | C35 | 121.3(2) |
| C44 | O2 | C45 | 117.7(3) |  | C33 | C34 | C35 | 120.6(2) |
| C24 | N5 | B1 | 121.3(2) |  | C28 | C33 | C34 | 119.8(2) |
| C17 | N5 | C24 | 113.1(2) |  | C32 | C33 | C34 | 122.2(2) |
| C17 | N5 | B1 | 124.0(2) |  | C32 | C33 | C28 | 118.0(2) |
| C16 | N3 | C9 | 112.3(2) |  | C27 | C26 | C25 | 120.9(2) |
| C16 | N3 | B1 | 124.0(2) |  | C27 | C28 | C33 | 119.5(2) |
| C9 | N3 | B1 | 122.1(2) |  | C29 | C28 | C33 | 119.1(3) |
| C24 | N6 | C1 | 116.9(2) |  | C29 | C28 | C27 | 121.4(3) |
| C17 | N4 | C16 | 116.6(2) |  | C19 | C20 | C21 | 121.6(3) |
| C9 | N2 | C8 | 117.2(2) |  | C44 | C35 | C34 | 120.5(3) |
| C18 | C23 | C24 | 107.3(2) |  | C44 | C35 | C36 | 119.0(3) |
| C22 | C23 | C24 | 132.1(2) |  | C36 | C35 | C34 | 120.5(3) |
| C22 | C23 | C18 | 120.4(2) |  | C26 | C27 | C28 | 120.0(2) |
| N1 | C1 | C2 | 105.7(2) |  | C5 | C6 | C7 | 118.2(3) |
| N6 | C1 | N1 | 122.1(2) |  | C10 | C11 | C12 | 117.6(3) |
| N6 | C1 | C2 | 130.3(2) |  | C4 | C3 | C2 | 118.0(3) |
| N5 | C24 | C23 | 105.6(2) |  | C20 | C19 | C18 | 118.1(3) |
| N6 | C24 | N5 | 123.3(2) |  | C22 | C21 | C20 | 120.9(3) |
| N6 | C24 | C23 | 129.0(2) |  | C13 | C14 | C15 | 117.8(3) |
| N3 | C16 | C15 | 106.2(2) |  | C38 | C37 | C36 | 118.5(3) |
| N4 | C16 | N3 | 122.7(2) |  | C6 | C5 | C4 | 121.1(3) |
| N4 | C16 | C15 | 129.6(2) |  | C13 | C12 | C11 | 121.7(3) |
| C2 | C7 | C8 | 107.1(2) |  | C3 | C4 | C5 | 121.6(3) |
| C6 | C7 | C8 | 132.3(2) |  | C30 | C29 | C28 | 121.3(3) |
| C6 | C7 | C2 | 120.5(2) |  | C12 | C13 | C14 | 121.6(3) |
| C23 | C18 | C17 | 106.8(2) |  | C31 | C32 | C33 | 121.0(3) |
| C19 | C18 | C23 | 120.6(2) |  | O2 | C44 | C35 | 117.6(2) |
| C19 | C18 | C17 | 132.4(2) |  | O2 | C44 | C43 | 120.3(3) |
| N5 | C17 | C18 | 106.0(2) |  | C35 | C44 | C43 | 122.1(3) |
| N4 | C17 | N5 | 123.2(2) |  | C35 | C36 | C41 | 118.2(3) |
| N4 | C17 | C18 | 129.0(2) |  | C37 | C36 | C35 | 121.4(3) |
| N1 | C8 | C7 | 105.4(2) |  | C37 | C36 | C41 | 120.4(3) |
| N2 | C8 | N1 | 122.2(2) |  | C29 | C30 | C31 | 120.1(3) |
| N2 | C8 | C7 | 131.0(2) |  | C42 | C41 | C36 | 118.9(3) |
| C7 | C2 | C1 | 107.2(2) |  | C40 | C41 | C36 | 118.5(3) |
| C3 | C2 | C1 | 132.1(2) |  | C40 | C41 | C42 | 122.5(3) |
| C3 | C2 | C7 | 120.6(2) |  | C32 | C31 | C30 | 120.4(3) |
| C15 | C10 | C9 | 107.5(2) |  | C42 | C43 | C44 | 119.2(3) |
| C11 | C10 | C9 | 132.1(3) |  | C43 | C42 | C41 | 122.5(3) |
| C11 | C10 | C15 | 120.4(2) |  | O1 | B1 | N1 | 108.8(2) |
| O1 | C25 | C34 | 120.9(2) |  | O1 | B1 | N5 | 116.8(2) |
| O1 | C25 | C26 | 117.4(2) |  | O1 | B1 | N3 | 118.8(2) |
| C34 | C25 | C26 | 121.7(2) |  | N1 | B1 | N5 | 104.2(2) |
| N3 | C9 | C10 | 105.6(2) |  | N1 | B1 | N3 | 103.59(19) |
| N2 | C9 | N3 | 122.8(2) |  | N5 | B1 | N3 | 103.0(2) |
| N2 | C9 | C10 | 129.8(2) |  | C39 | C40 | C41 | 121.7(3) |
| C21 | C22 | C23 | 118.4(3) |  | C37 | C38 | C39 | 121.5(3) |
| C10 | C15 | C16 | 106.7(2) |  | C40 | C39 | C38 | 119.2(3) |

| **Table 6-2-6** Torsion Angles for **1c**. | | | | | | | | | | |
| --- | --- | --- | --- | --- | --- | --- | --- | --- | --- | --- |
| **A** | **B** | **C** | **D** | **Angle/˚** |  | **A** | **B** | **C** | **D** | **Angle/˚** |
| O1 | C25 | C34 | C33 | -177.2(2) |  | C9 | N2 | C8 | C7 | -156.8(2) |
| O1 | C25 | C34 | C35 | 3.2(4) |  | C9 | C10 | C15 | C16 | -1.2(3) |
| O1 | C25 | C26 | C27 | 178.6(2) |  | C9 | C10 | C15 | C14 | 175.8(2) |
| N1 | C1 | C2 | C7 | 5.4(3) |  | C9 | C10 | C11 | C12 | -178.8(3) |
| N1 | C1 | C2 | C3 | -178.6(3) |  | C22 | C23 | C24 | N5 | 178.8(2) |
| O2 | C44 | C43 | C42 | -179.6(3) |  | C22 | C23 | C24 | N6 | -17.3(4) |
| N3 | C16 | C15 | C10 | -6.7(3) |  | C22 | C23 | C18 | C17 | 175.6(2) |
| N3 | C16 | C15 | C14 | 176.7(3) |  | C22 | C23 | C18 | C19 | 0.7(4) |
| N6 | C1 | C2 | C7 | -158.6(2) |  | C15 | C10 | C9 | N3 | 8.7(3) |
| N6 | C1 | C2 | C3 | 17.4(5) |  | C15 | C10 | C9 | N2 | -156.1(2) |
| N4 | C16 | C15 | C10 | 159.0(2) |  | C15 | C10 | C11 | C12 | -1.1(4) |
| N4 | C16 | C15 | C14 | -17.6(4) |  | C15 | C14 | C13 | C12 | -2.1(4) |
| C23 | C18 | C17 | N5 | 5.9(2) |  | C34 | C25 | C26 | C27 | 2.4(4) |
| C23 | C18 | C17 | N4 | -158.9(2) |  | C34 | C33 | C28 | C27 | 3.0(4) |
| C23 | C18 | C19 | C20 | -1.0(4) |  | C34 | C33 | C28 | C29 | -176.9(2) |
| C23 | C22 | C21 | C20 | -1.5(4) |  | C34 | C33 | C32 | C31 | 178.1(3) |
| C1 | N1 | C8 | N2 | -157.3(2) |  | C34 | C35 | C44 | O2 | -0.6(4) |
| C1 | N1 | C8 | C7 | 10.5(3) |  | C34 | C35 | C44 | C43 | -179.6(2) |
| C1 | N1 | B1 | O1 | -92.2(3) |  | C34 | C35 | C36 | C37 | -3.0(4) |
| C1 | N1 | B1 | N5 | 33.1(3) |  | C34 | C35 | C36 | C41 | 179.8(2) |
| C1 | N1 | B1 | N3 | 140.6(2) |  | C33 | C34 | C35 | C44 | -65.0(3) |
| C1 | N6 | C24 | N5 | 9.4(3) |  | C33 | C34 | C35 | C36 | 113.8(3) |
| C1 | N6 | C24 | C23 | -152.0(2) |  | C33 | C28 | C27 | C26 | -1.7(4) |
| C1 | C2 | C3 | C4 | -177.1(3) |  | C33 | C28 | C29 | C30 | -2.7(4) |
| C24 | N5 | C17 | N4 | 154.7(2) |  | C33 | C32 | C31 | C30 | 0.4(5) |
| C24 | N5 | C17 | C18 | -11.2(3) |  | C26 | C25 | C34 | C33 | -1.1(4) |
| C24 | N5 | B1 | O1 | 89.2(3) |  | C26 | C25 | C34 | C35 | 179.3(2) |
| C24 | N5 | B1 | N1 | -30.8(3) |  | C28 | C33 | C32 | C31 | -2.7(4) |
| C24 | N5 | B1 | N3 | -138.7(2) |  | C28 | C29 | C30 | C31 | 0.4(5) |
| C24 | N6 | C1 | N1 | -7.5(3) |  | C35 | C34 | C33 | C28 | 178.0(2) |
| C24 | N6 | C1 | C2 | 154.3(2) |  | C35 | C34 | C33 | C32 | -2.8(4) |
| C24 | C23 | C18 | C17 | 0.8(2) |  | C35 | C44 | C43 | C42 | -0.7(4) |
| C24 | C23 | C18 | C19 | -174.2(2) |  | C35 | C36 | C41 | C42 | 0.4(4) |
| C24 | C23 | C22 | C21 | 173.9(2) |  | C35 | C36 | C41 | C40 | 178.3(3) |
| C16 | N3 | C9 | N2 | 152.5(2) |  | C27 | C28 | C29 | C30 | 177.4(3) |
| C16 | N3 | C9 | C10 | -13.6(3) |  | C6 | C7 | C8 | N1 | 177.0(3) |
| C16 | N3 | B1 | O1 | 104.8(3) |  | C6 | C7 | C8 | N2 | -16.7(5) |
| C16 | N3 | B1 | N1 | -134.5(2) |  | C6 | C7 | C2 | C1 | 177.8(2) |
| C16 | N3 | B1 | N5 | -26.2(3) |  | C6 | C7 | C2 | C3 | 1.2(4) |
| C16 | N4 | C17 | N5 | -8.8(3) |  | C6 | C5 | C4 | C3 | 0.9(5) |
| C16 | N4 | C17 | C18 | 153.6(2) |  | C11 | C10 | C9 | N3 | -173.4(3) |
| C16 | C15 | C14 | C13 | -179.8(3) |  | C11 | C10 | C9 | N2 | 21.8(4) |
| C7 | C2 | C3 | C4 | -1.6(4) |  | C11 | C10 | C15 | C16 | -179.5(2) |
| C7 | C6 | C5 | C4 | -1.3(4) |  | C11 | C10 | C15 | C14 | -2.4(4) |
| C18 | C23 | C24 | N5 | -7.2(2) |  | C11 | C12 | C13 | C14 | -1.4(4) |
| C18 | C23 | C24 | N6 | 156.8(2) |  | C19 | C18 | C17 | N5 | -180.0(3) |
| C18 | C23 | C22 | C21 | 0.6(4) |  | C19 | C18 | C17 | N4 | 15.2(4) |
| C17 | N5 | C24 | N6 | -153.5(2) |  | C19 | C20 | C21 | C22 | 1.2(4) |
| C17 | N5 | C24 | C23 | 11.6(3) |  | C21 | C20 | C19 | C18 | 0.1(4) |
| C17 | N5 | B1 | O1 | -106.5(3) |  | C37 | C36 | C41 | C42 | -176.9(3) |
| C17 | N5 | B1 | N1 | 133.5(2) |  | C37 | C36 | C41 | C40 | 1.0(4) |
| C17 | N5 | B1 | N3 | 25.6(3) |  | C37 | C38 | C39 | C40 | 2.9(5) |
| C17 | N4 | C16 | N3 | 8.2(3) |  | C29 | C28 | C27 | C26 | 178.2(3) |
| C17 | N4 | C16 | C15 | -155.4(2) |  | C29 | C30 | C31 | C32 | 0.8(5) |
| C17 | C18 | C19 | C20 | -174.4(2) |  | C32 | C33 | C28 | C27 | -176.3(3) |
| C8 | N1 | C1 | N6 | 155.5(2) |  | C32 | C33 | C28 | C29 | 3.8(4) |
| C8 | N1 | C1 | C2 | -10.1(3) |  | C44 | C35 | C36 | C37 | 175.8(2) |
| C8 | N1 | B1 | O1 | 96.3(3) |  | C44 | C35 | C36 | C41 | -1.4(4) |
| C8 | N1 | B1 | N5 | -138.4(2) |  | C44 | C43 | C42 | C41 | -0.3(4) |
| C8 | N1 | B1 | N3 | -30.9(3) |  | C36 | C35 | C44 | O2 | -179.5(2) |
| C8 | N2 | C9 | N3 | -7.9(3) |  | C36 | C35 | C44 | C43 | 1.6(4) |
| C8 | N2 | C9 | C10 | 154.6(2) |  | C36 | C37 | C38 | C39 | -4.8(4) |
| C8 | C7 | C2 | C1 | 0.6(3) |  | C36 | C41 | C42 | C43 | 0.5(4) |
| C8 | C7 | C2 | C3 | -175.9(2) |  | C36 | C41 | C40 | C39 | -3.0(5) |
| C8 | C7 | C6 | C5 | 176.5(3) |  | C41 | C40 | C39 | C38 | 1.1(5) |
| C2 | C7 | C8 | N1 | -6.4(3) |  | C45 | O2 | C44 | C35 | 167.0(2) |
| C2 | C7 | C8 | N2 | 159.9(3) |  | C45 | O2 | C44 | C43 | -14.1(4) |
| C2 | C7 | C6 | C5 | 0.2(4) |  | C42 | C41 | C40 | C39 | 174.8(3) |
| C2 | C3 | C4 | C5 | 0.6(4) |  | B1 | O1 | C25 | C34 | -96.9(3) |
| C10 | C15 | C14 | C13 | 4.0(4) |  | B1 | O1 | C25 | C26 | 86.8(3) |
| C10 | C11 | C12 | C13 | 2.9(4) |  | B1 | N1 | C1 | N6 | -16.7(3) |
| C25 | O1 | B1 | N1 | -179.8(2) |  | B1 | N1 | C1 | C2 | 177.6(2) |
| C25 | O1 | B1 | N5 | 62.7(3) |  | B1 | N1 | C8 | N2 | 14.8(4) |
| C25 | O1 | B1 | N3 | -61.8(3) |  | B1 | N1 | C8 | C7 | -177.4(2) |
| C25 | C34 | C33 | C28 | -1.5(4) |  | B1 | N5 | C24 | N6 | 12.4(3) |
| C25 | C34 | C33 | C32 | 177.7(3) |  | B1 | N5 | C24 | C23 | 177.5(2) |
| C25 | C34 | C35 | C44 | 114.5(3) |  | B1 | N5 | C17 | N4 | -10.7(4) |
| C25 | C34 | C35 | C36 | -66.6(3) |  | B1 | N5 | C17 | C18 | -176.6(2) |
| C25 | C26 | C27 | C28 | -0.9(4) |  | B1 | N3 | C16 | N4 | 11.9(4) |
| C9 | N3 | C16 | N4 | -154.1(2) |  | B1 | N3 | C16 | C15 | 178.8(2) |
| C9 | N3 | C16 | C15 | 12.9(3) |  | B1 | N3 | C9 | N2 | -13.7(3) |
| C9 | N3 | B1 | O1 | -90.6(3) |  | B1 | N3 | C9 | C10 | -179.8(2) |
| C9 | N3 | B1 | N1 | 30.1(3) |  | C40 | C41 | C42 | C43 | -177.3(3) |
| C9 | N3 | B1 | N5 | 138.5(2) |  | C38 | C37 | C36 | C35 | -174.3(3) |
| C9 | N2 | C8 | N1 | 7.6(3) |  | C38 | C37 | C36 | C41 | 2.8(4) |

| **Table 6-2-7** Hydrogen Atom Coordinates (Å×10^4^) and Isotropic Displacement Parameters (Å^2^×10^3^) for **1c**. | | | | |
| --- | --- | --- | --- | --- |
| **Atom** | ***x*** | ***y*** | ***z*** | **U(eq)** |
| H22 | 2025.99 | 1664.56 | 2501.73 | 37 |
| H26 | 7150.52 | 5869.36 | 2890.94 | 38 |
| H20 | 150.84 | 4246.17 | 2785.96 | 43 |
| H27 | 7055.16 | 7457.59 | 2299.46 | 40 |
| H6 | 7897.88 | 1311.29 | 4883.11 | 40 |
| H11 | 7529.03 | 5447 | 5409.95 | 44 |
| H3 | 5277.3 | -446.53 | 3458.69 | 40 |
| H19 | 1604.79 | 5368.72 | 3071.81 | 38 |
| H21 | 352.05 | 2428.74 | 2486.58 | 43 |
| H14 | 4428.31 | 7319.87 | 4551.48 | 43 |
| H37 | 6835.85 | 3533.36 | 1518.58 | 49 |
| H5 | 7680.06 | -586.14 | 4904.79 | 45 |
| H12 | 6974.94 | 7045.52 | 5886.79 | 52 |
| H4 | 6377.6 | -1446.28 | 4212.34 | 46 |
| H29 | 6256.88 | 8499.76 | 1356.46 | 46 |
| H13 | 5448.73 | 7937.9 | 5477.88 | 49 |
| H32 | 4411.35 | 5379.28 | 561.24 | 48 |
| H30 | 5240.59 | 8555.04 | 386.8 | 53 |
| H31 | 4317.31 | 6979.32 | -18.7 | 55 |
| H43 | 2328.13 | 3139.86 | 1185.86 | 54 |
| H45A | 1996.94 | 4965.49 | 927.33 | 80 |
| H45B | 1940.23 | 5859.85 | 1471.18 | 80 |
| H45C | 1765.01 | 4593.02 | 1616.66 | 80 |
| H42 | 3106.55 | 1556.58 | 955.06 | 58 |
| H40 | 4697.06 | 481.91 | 849.88 | 60 |
| H38 | 7564.13 | 1962.97 | 1139.92 | 59 |
| H39 | 6496.93 | 386.96 | 844.57 | 58 |

§7 Magnetic Conductive Atomic Force Microscopy (mc-AFM) Experiments

Magnetic conductive atomic force microscopy (mc-AFM) measurements were carried out using a Bruker Multimode 8 equipped with a conductive AFM module.

A solution of 1c (3 mg in 1 mL of toluene) was prepared and filtered through a membrane filter with a pore size of 0.45 μm. The resulting solution was deposited onto a HOPG (Veeco, ZYH) substrate by spin coating (3000 rpm for 30 s). The substrate was then mounted onto the AFM apparatus using a conductive paste (Fujikura Kasei, Dotite).

A Bruker MFMV AFM tip was magnetized for 20 min using a Nd magnet (Niroku Seisakusho Co., Ltd.) with a surface magnetic flux density of 0.49 T. Conductivity measurements were performed at various positions on the substrate, with a total of 40 measurements. After reversing the magnetization direction of the tip, an additional 40 measurements were carried out. The direction of the tip magnetization was defined as follows.
All measured current–voltage (I–V) curves are shown in pale colors, and the averages are plotted as solid lines. The spin polarization percentage (SP%) is presented as a function of the applied bias. The blue line represents the current when the magnet’s north pole is oriented upward relative to the substrate, whereas the purple line corresponds to the current when the magnet’s north pole is oriented downward.

**Figure SI-21** Definition of magnetization direction.

SP (%) = $\frac{\text{I}_{\text{up}}\text{ }-\text{ }\text{I}_{\text{down}}}{\text{I}_{\text{up}}\text{ + }\text{I}_{\text{down}}}$× 100%

[1]	W. H. Pirkle, J. L. Schreiner, *J. Org. Chem.* **1981**, *46*, 4988-4991.
