# Supplementary material for: Coordination Chemistry of Boron Subphthalocyanine With BINOL Derivatives: Unexpected Oxidation to Oxa[4]Helicene and Properties of the Resulting Axially Coordinated Complexes Containing Oxa[4]Helicene and/or BINOL Derivatives
Source: Chemistry. 2026 Jul 25;32(40):e71412. doi: 10.1002/chem.71412 (PMC13716020; doi:10.1002/chem.71412)
Supplement: Supplementary file 1 [file CHEM-32-e71412-s001.zip › REVISED_chem71412-sup-0001-SuppMat.pdf]

Supporting Information

Coordination Chemistry of Boron Subphthalocyanine with BINOL Derivatives: Unexpected Oxidation to Oxa[4]helicene and Properties of the Resulting Axially Coordinated Complexes Containing Oxa[4]helicene and/or BINOL Derivatives

Shafikul Islam,[a,b] Kazuyuki Asahi,[c] Hiroyuki Nishikawa,* [c] Daiki Tauchi,[d] Masashi Hasegawa,* [d], and Ken-ichi Sugiura* [a]

[a] *Department of Chemistry, Graduate School of Science, Tokyo Metropolitan University, 1-1 Minami-Osawa, Hachi-Oji, Tokyo 192-0397, Japan*

[b] *Department of Chemistry, Jahangirnagar University, Savar, Dhaka-1342, Bangladesh*

[c] *Graduate School of Science and Engineering, Ibaraki University, 2-1-1 Bunkyo, Mito, Ibaraki 310-8512, Japan*

[d] *Department of Chemistry, Graduate School of Science, Kitasato University, 1–15-1 Kitasato Minami-ku, Sagamihara, Kanagawa 252–0373, Japan*

§1 Synthesis

1-1 General

The following chemicals were prepared according to the literature. Toluene (Kanto Chemical Co., Ltd.) used in the complex formation reaction was distilled over P_2O_5 prior to use. All other chemicals were used as received. Methoxy-BINOL was also prepared according to the literature.[1] Thin-layer chromatography (TLC) was performed using Merck/Supelco silica gel 60 F254 plates. Silica gel flash column chromatography was performed using a Silica Gel 60N (40–50 μm, spherical, neutral) from Kanto Chemical Co., Inc. An EARTH MAN CLT-120 clip light (200 W, Takagi Co., Ltd., Japan) was used as the light source in the light-irradiation control experiment (CE#2).

1-2 Synthesis of the complexes, **1b** and **1c**.

(General Procedure)

A mixture of chloroboronsubphthalocyanine **1d** (83.4 mg, 194 μmol, TCI Co., Ltd.), silver triflate (67.3 mg, 262 μmol, 1.4 equiv, TCI Co., Ltd.), and distilled toluene (3 mL) was purged with nitrogen and stirred at room temperature for 2 h. During the reaction, the color of the mixture changed from pink to violet. The progress of the reaction was monitored by TLC (R_f = 0.78 for **1d** and R_f = 0.04 for **1e** in toluene/ethyl acetate = 9:1). To avoid formation of the μ-oxo dimer of subphthalocyanine (R_f = 0.18), the reaction time at this stage should be minimized.

Subsequently, *rac*-methoxyBINOL (116.3 mg, 387 μmol, 2.0 equiv)[1] and diisopropylethylamine (31.3 mg, 242 μmol, 1.25 equiv, TCI Co., Ltd.) were added, and the mixture was stirred at 50 °C for 14 h. This stage was also monitored by TLC (R_f = 0.72 for **1c**). The reaction was quenched, and the solvent was removed under reduced pressure. The residue was subjected to column chromatography on silica gel, eluting with toluene/ethyl acetate (9:1), to afford the crude product. Further purification by silica gel chromatography using the same eluent yielded compound **1c** as a pinkish-red solid (72.1 mg, 54%). The μ-oxo dimer of subphthalocyanine was also isolated as a byproduct (24.9 mg, 32%).

Similar reactions were carried out using the following reagents to afford **1b** and (*R*)- and (*S*)-**1c**.

Table SI-1 Summary of complex formation reactions.

**1d**	AgOTf	PhMe	BINOLs	iPr_2NEt	Reaction time	Products (yield)
86.3 mg (1 eq.)	66.8 mg (1.3 eq.)	3.0 mL	(*R*)-methoxy BINOL 120.7 mg (2.0 eq.)	31.3 mg (1.3 eq.)	16h At 50 °C	(*R*)-**1c** (73.1 mg, 53%) μ-oxo dimer (25.1 mg, 31%)
90.2 mg (1 eq.)	69.8 mg (1.3 eq.)	3.0 mL	(*S*)-methoxy BINOL 126.3 mg (2.0 eq.)	33.8 mg (1.3 eq.)	16h At 50 °C	(*S*)-**1c** 75.2 mg, 52% μ-oxo dimer (27.8 mg, 32%)
52.2 mg (1 eq.)	40.0 mg (1.3 eq.)	4.0 mL	*rac*-BINOL 104.1 mg	17.5 mg (1.1 eq.)	16h At 40 °C	**1b** 44.5 mg, 54%

			(3.0 eq.)			μ-oxo dimer (15.2 mg, 31%)
51.8 mg (1 eq.)	41.1 mg (1.3 eq.)	3.0 mL	*(R)*-BINOL 137.5 mg (4.0 eq.)	13.3 mg (1.1 eq.)	16h At 40 °C	**1b** 43.2 mg, 53% μ-oxo dimer (14.8 mg, 30%)
50.2 mg (1 eq.)	40.5 mg (1.3 eq.)	3.0 mL	*(S)* -BINOL 135.8 mg (4.0 eq.)	13.3 mg (1.1 eq.)	16h At 40 °C	**1b** 41.8 mg, 53% μ-oxo dimer (14.6 mg, 31%)

1c: ^{1}H NMR (500 MHz, $CDCl_3$) δ 8.71-8.66 (*AA'BB'*-spin system, 6H), 7.87 (d, *J* = 8.9Hz, 1H), 7.84-7.80 (*AA'BB'*-spin system, 6H), 7.69 (d, *J* =8.2, 1H), 7.59 (d, *J* = 8.0Hz, 1H), 7.34 (d, *J* = 9.0, 1H), 7.13 (dt, *J* = 6.6 and 1.0 Hz, 1H), 7.09 (d, *J* = 9.0 Hz, 1H), 7.03 (dt, *J* = 6.6 and 0.85 Hz, 1H), 6.96 (dt, *J* = 6.7 and 1.2 Hz, 1H), 6.80 (dt, *J* = 6.6 and 1.2 Hz, 1H), 6.72 (d, *J* = 8.7 Hz, 1H), 6.37 (d, *J* = 8.1 Hz, 1H), 5.59 (d, *J* = 8.8 Hz, 1H), and 3.24 (s, 3H) ppm; ^{13}C NMR (125 MHz, CDCl3) δ 154.36 (quaternary, q, determined by DEPT-135), 151.13 (q), 151.05 (q), 148.16 (q), 133.51 (q), 133.05 (q), 130.76 (q), 129.47 (q), 129.40 (tertiary, t), 129.07 (t), 128.57 (q), 128.53 (t), 127.60 (t), 127.34 (t), 125.99 (t), 125.62 (t), 125.48 (t), 125.00 (t), 123.39 (t), 123.24 (t), 122.01 (t), 120.46 (t), 118.65 (q), 113.78 (t), and 56.55 (methyl) ppm; HR-MALDI-TOF mass (m/z) Calcd for $C_{45}H_{27}BN_6O_2$ $[M]^+$ 694.2295, found $[M]^+$ 694.8883; UV (CH_2Cl_2) λmax (10^4 ε $dm^3mol^{-1}cm^{-1}$) 271 (4.27), 300 (4.37), and 564 (8.71) nm; emission (nm) 577 nm (excitation at 564nm, quantum yield: 13%).

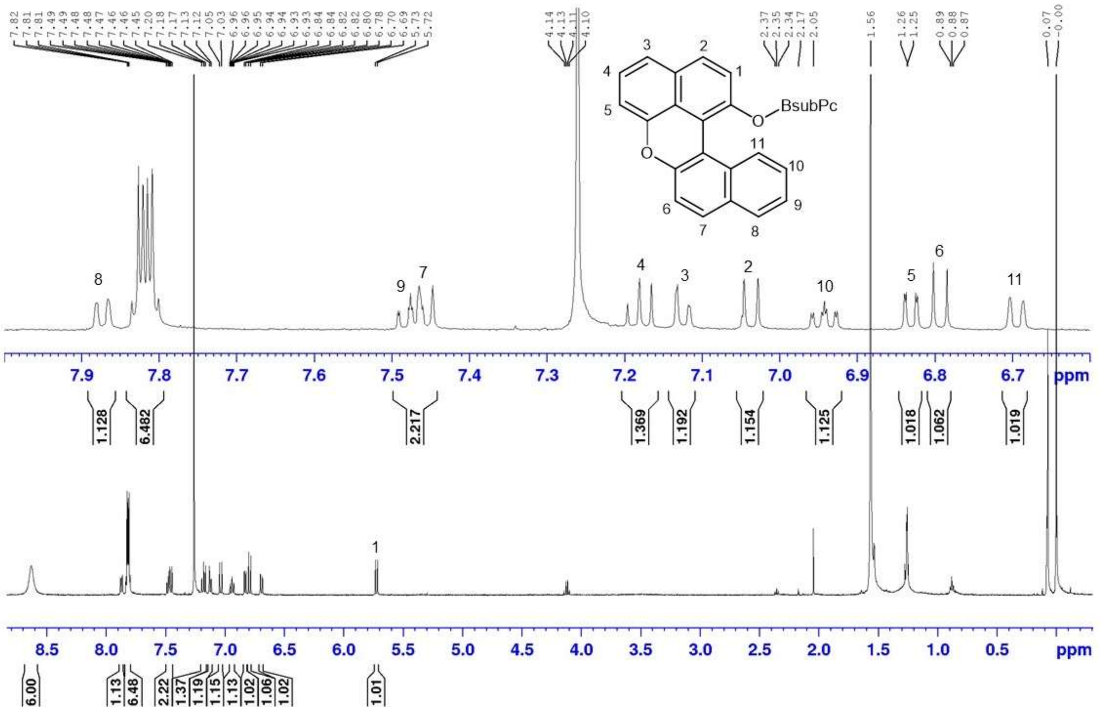

Figure SI-1 ^{1}H NMR spectrum of **1b** in $CDCl_3$.

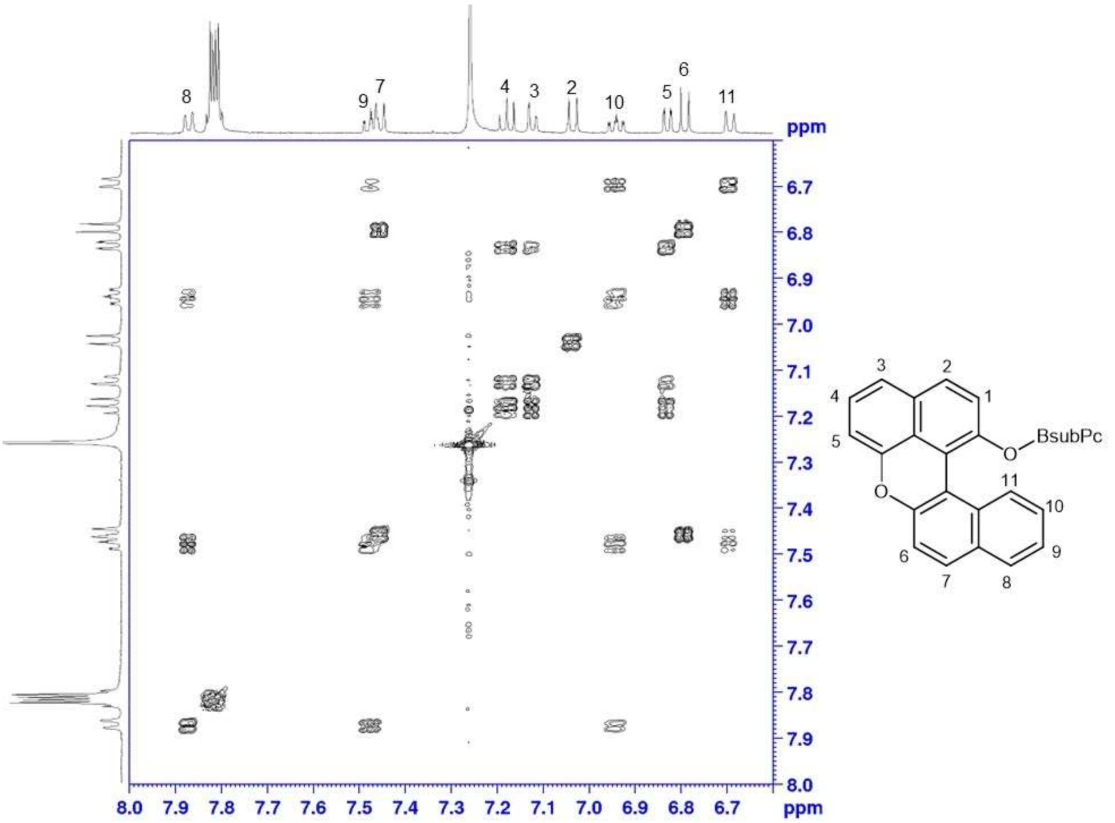

Figure SI-2 COSY spectrum of **1b** in $CDCl_3$.

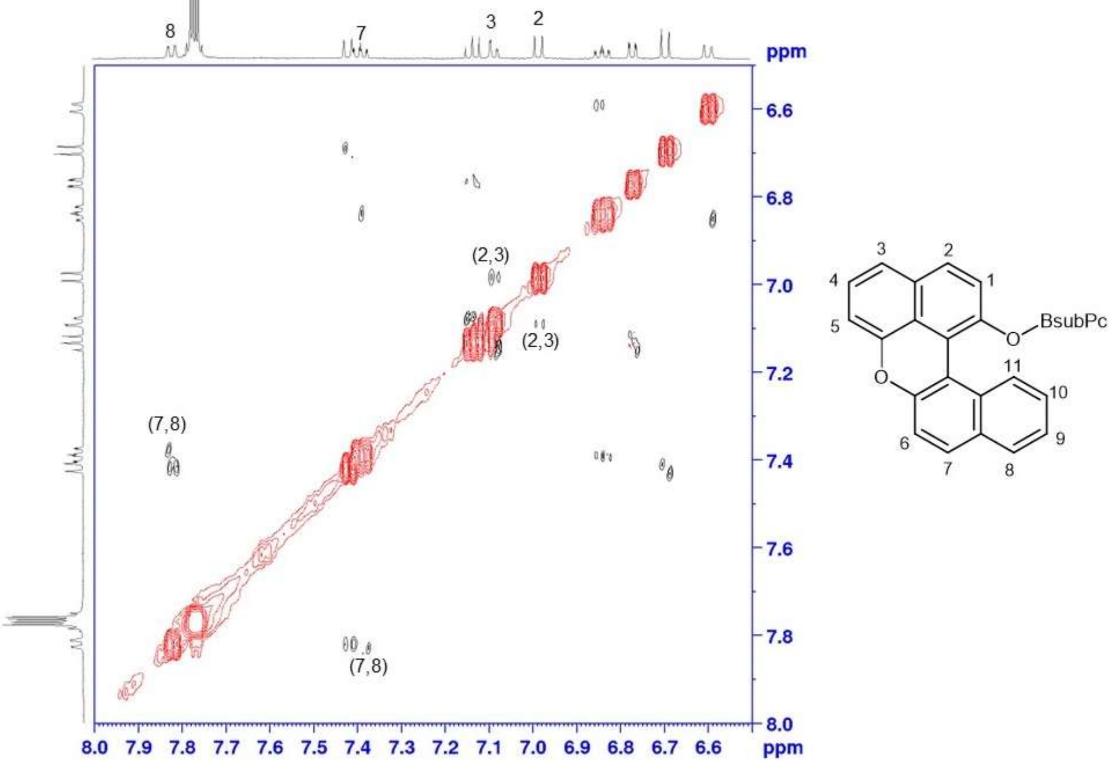

Figure SI-3 NOESY spectrum of **1b** in CD_2Cl_2.

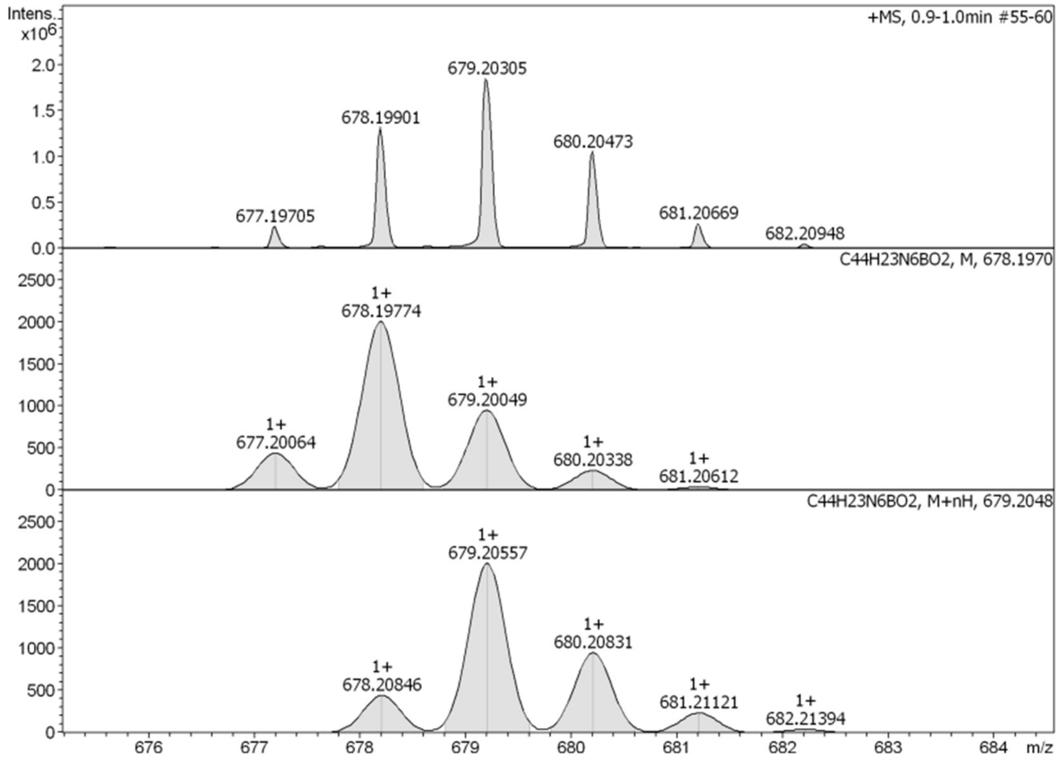

Figure SI-4 APCI mass spectrum of **1b**.

1c: ^{1}H NMR (500 MHz, $CDCl_3$) δ 8.63 (broad s, 6H), 7.87 (d, *J* = 8.0 Hz, 1H), 7.83-7.80 (*AA'BB'*-spin system, 6H), 7.48 (t, *J* = 8.0 Hz, 1H), 7.45 (d, *J* = 8.6 Hz, 1H), 7.18 (t, *J* = 7.9 Hz, 1H), 7.12 (d, *J* = 7.2 Hz, 1H), 7.03 (d, *J* = 8.8 Hz, 1H), 6.94 (dt, *J* = 6.8 and 1.3 Hz, 1H), 6.83 (dd, *J* = 7.4 and 1.1 Hz, 1H), 6.79 (d, *J* = 8.7 Hz, 1H), 6.69 (d, *J* = 8.5 Hz, 1H), and 5.72 (d, *J* = 8.8 Hz, 1H) ppm; 151.74 (q), 149.23 (q), 149.20 (q), 142.04 (q), 130.12 (q), 129.92 (q), 129.62 (q), 129.02 (t), 128.35 (t), 128.00 (q), 126.97 (q), 126.53 (t), 125.98 (t), 125.23 (t), 124.48 (q), 124.26 (t), 124.03 (t), 123.25 (t), 123.18 (t), 120.96 (t), 118.39 (t), 115.07 (t), 114.26 (q), 111.83 (q), and 106.53 (t) ppm; HR-MALDI-TOF mass (m/z) Calcd for $C_{44}H_{23}BN_6O_2$ $[M]^+$ 678.1982, found $[M]^+$678.9257; UV (CH_2Cl_2) λmax (10^4 ε $dm^3mol^{-1}cm^{-1}$) 263 (6.90), 304 (5.67), and 566 (9.04) nm; emission (nm) 578 nm (excitation at 566nm, quantum yield: 4%).

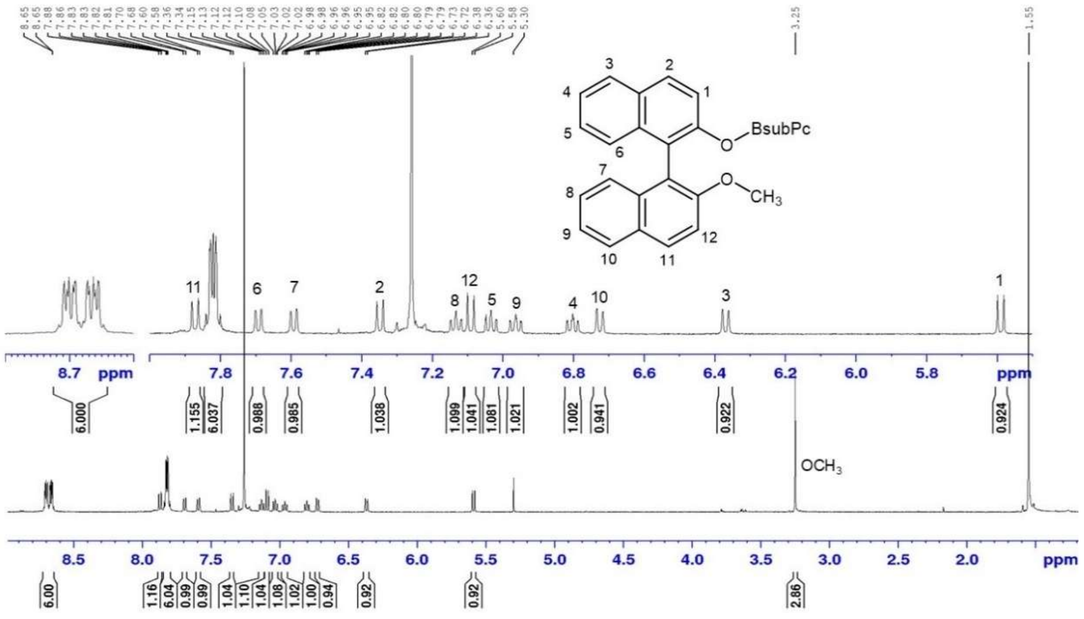

Figure SI-5 ^{1}H NMR spectrum of **1c** in $CDCl_3$.

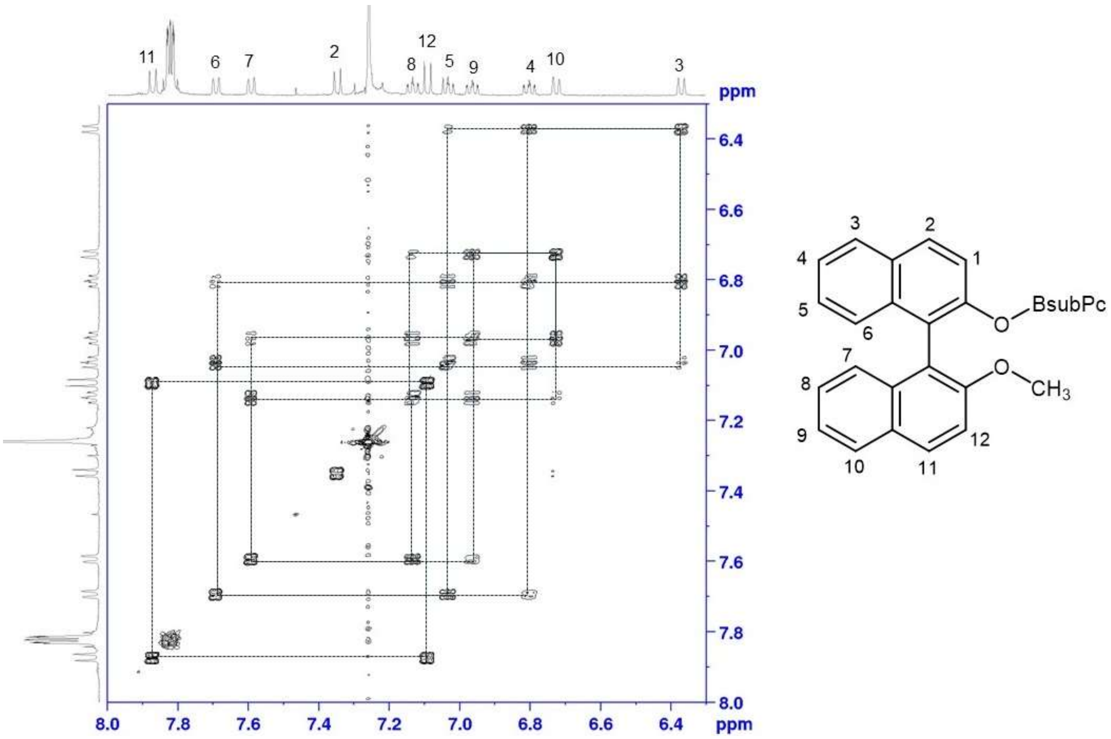

Figure SI-6 COSY spectrum of **1c** in $CDCl_3$.

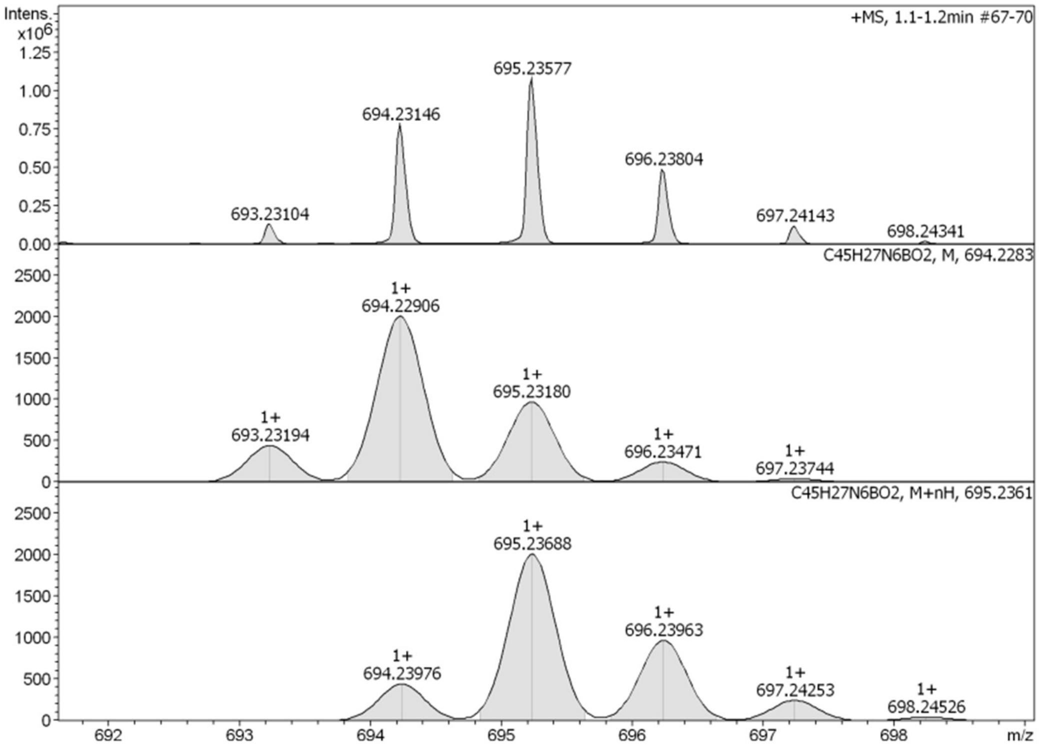

Figure SI-7 APCI mass spectrum of **1c**.

§2 Electrochemistry

Cyclic voltammetry (CV) and differential pulse voltammetry (DPV) were recorded using a BAS instrument with a glassy carbon working electrode, an Ag/AgCl reference electrode, and a platinum counter electrode. Distilled CH_2Cl_2 was used as the solvent, and recrystallized tetrabutylammonium PF_6 was used as the supporting electrolyte. The potentials were calibrated against the ferrocene/ferrocenium redox couple and referenced to 0 V.

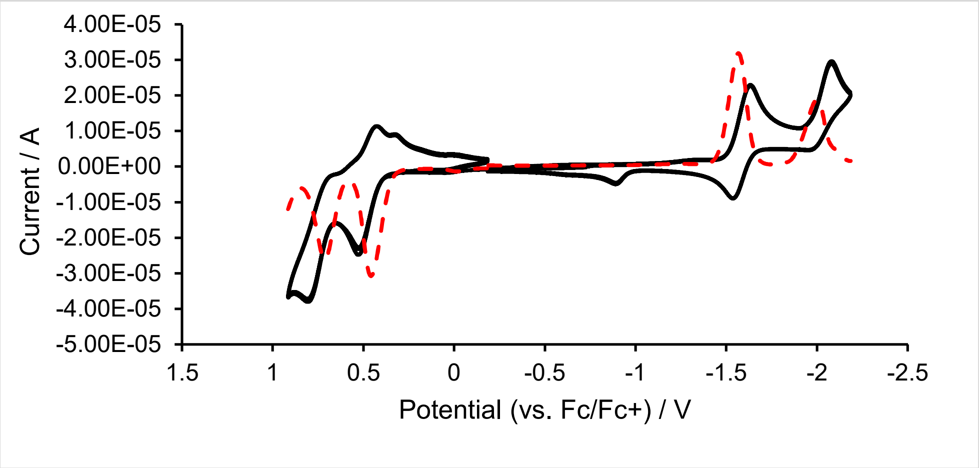

Figure SI-8 Cyclic and differential pulse voltammograms of **1b**.

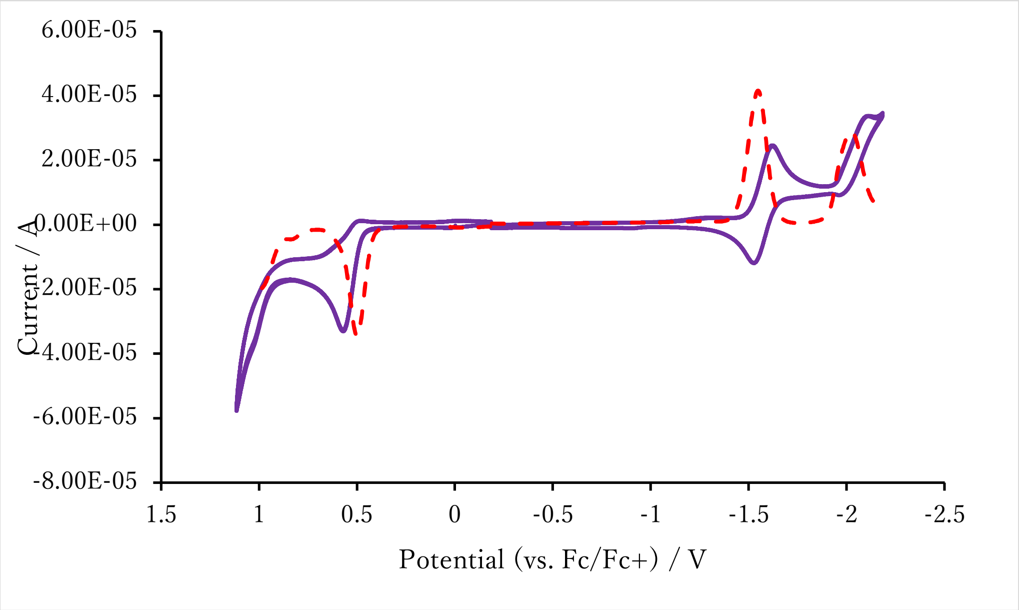

Figure SI-9 Cyclic and differential pulse voltammograms of **1c**.

Table SI-2 Summary of electrochemical studies
(V, vs ferrocene/ferrocenium, in CH_2Cl_2, $TBAPF_6$ as supporting electrolyte.).

**Compound**	**Ox2**	**Ox1**	**Red1**	**Red2**
SubPc(BINOLate)	0.71 V	0.46 V	-1.57 V	-2.00 V
SubPc(O-MeBINOLate)		0.50 V	-1.56 V	-2.02 V

§3 Absorption/CD spectra of **1c** and their simulations.

All quantum chemical calculations were carried out using the B3LYP functional in combination with the 6-31G(d) basis set. The optimized coordinates of (*S*)-**1c** are as follows.

O	-0.11956200	0.03675205	-1.26359689
N	1.88677425	-1.08892925	-0.55609071
O	-2.89234318	0.39141009	1.66784289
N	0.65839621	0.04244330	1.12314016
N	1.92131975	1.27383867	-0.44827136
N	1.09701154	-2.28724637	1.33697939
N	1.18211371	2.32222756	1.55357536
N	3.54883864	0.12185039	-1.74772669
C	0.28450502	-0.76472339	3.19286306
C	1.79590152	-2.22917186	0.19349008
C	0.61030242	-1.13505778	1.82132100
C	1.87616417	2.34397294	0.40592991
C	3.58375439	-2.37548176	-1.28372732
C	0.31194910	0.66341698	3.26084242
C	0.65277537	1.14870406	1.92978359
C	3.01094245	-1.03573962	-1.33407402
C	2.82406512	-3.12168735	-0.32808509
C	3.66336834	2.56316523	-1.06233169
C	-1.10354385	0.97918885	-1.38739441
C	3.04579967	1.25435982	-1.23212886
C	0.06975836	-1.52640240	4.34423124
H	0.06937970	-2.61085160	4.29344235
C	2.93199553	3.24425223	-0.03790380
C	-2.40727121	0.67002217	-1.01819785
C	-3.44018520	1.64257702	-1.23304362
C	-0.78942229	2.23502830	-1.97402582
H	0.23388691	2.42170501	-2.28181525
C	-3.11187044	2.91774319	-1.80077967
C	-0.11226035	0.54922445	5.61470767
H	-0.27259437	1.03895802	6.57121158
C	-2.72752459	-0.66381046	-0.42191413
C	-1.76573716	3.18008195	-2.16440233
H	-1.51799912	4.14013743	-2.61140589

C	4.70232439	-2.94146775	-1.89919601
H	5.28790910	-2.36793382	-2.61100470
C	4.80056331	3.14489086	-1.62863805
H	5.36495691	2.61887984	-2.39239151
C	3.18854027	-4.42812302	0.00424677
H	2.62058880	-4.98680173	0.74163708
C	0.12764219	1.32006518	4.47984507
H	0.17047457	2.40358242	4.53249525
C	-0.14168769	-0.85746759	5.54771923
H	-0.32373066	-1.42889012	6.45372291
C	3.34284572	4.50193066	0.41093844
H	2.79677668	5.00930440	1.20017033
C	-2.56316216	-1.81394751	-2.63538576
H	-2.32020742	-0.86808824	-3.10692610
C	5.03711222	-4.25453007	-1.57847288
H	5.89311353	-4.72130333	-2.05773683
C	5.18138509	4.41009136	-1.19042199
H	6.05271302	4.88887017	-1.62845107
C	4.28932753	-4.98890880	-0.63831962
H	4.58103056	-6.00985602	-0.40836728
C	-4.13816975	3.87844232	-2.00352402
H	-3.86944406	4.84073380	-2.43406089
C	4.46106338	5.08069870	-0.18274560
H	4.78923553	6.06514191	0.13893519
C	-4.80212493	1.39012518	-0.90802235
H	-5.06982519	0.42380924	-0.49396411
C	-2.98523028	-0.76579528	0.94341912
C	-2.79548556	-1.84098215	-1.23044710
C	-5.44405716	3.60286207	-1.66917100
H	-6.22082808	4.34566561	-1.83009962
C	-3.12391244	-3.10199006	-0.62881459
C	-5.77513527	2.34165244	-1.11944332
H	-6.80868776	2.12126787	-0.86452320
C	-3.31302182	-2.01194437	1.53635218
H	-3.50487891	-2.07479192	2.60121358
C	-3.37762135	0.40650576	3.00176179
H	-4.42583037	0.08337208	3.05194785
H	-3.30416552	1.44492316	3.32971722

H	-2.76984190	-0.21999964	3.66615694
C	-3.37641592	-3.14755109	0.76330888
H	-3.62480813	-4.10147615	1.22278815
B	0.98655172	0.09346913	-0.34889839
C	-3.19533238	-4.26764928	-1.43892022
H	-3.44315143	-5.21459081	-0.96402046
C	-2.64351155	-2.96054382	-3.39172392
H	-2.46122770	-2.91240326	-4.46222125
C	-2.96005464	-4.20433116	-2.79195526
H	-3.01762598	-5.10146807	-3.40247056

Using the above optimized coordinates, TDDFT calculations were carried out at the B3LYP/6-31G(d) level. The results are plotted together with the experimentally obtained spectra (*rac*-**1c**) measured in CH_2Cl_2.

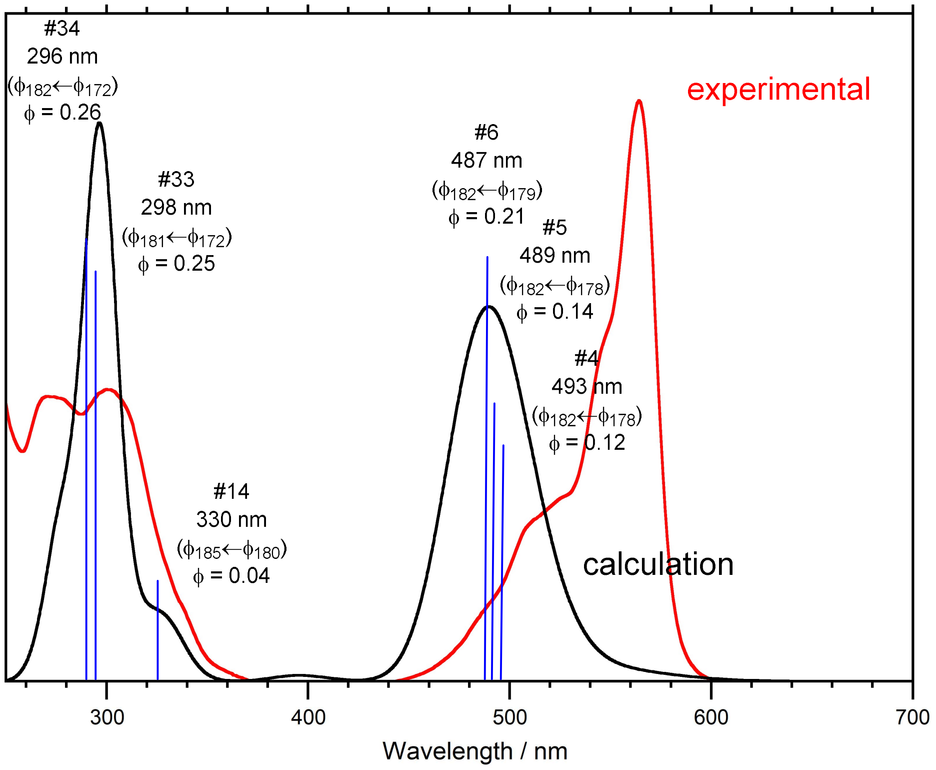

Figure SI-10 Absorption spectra of *rac*-**1c** and TDDFT simulation.

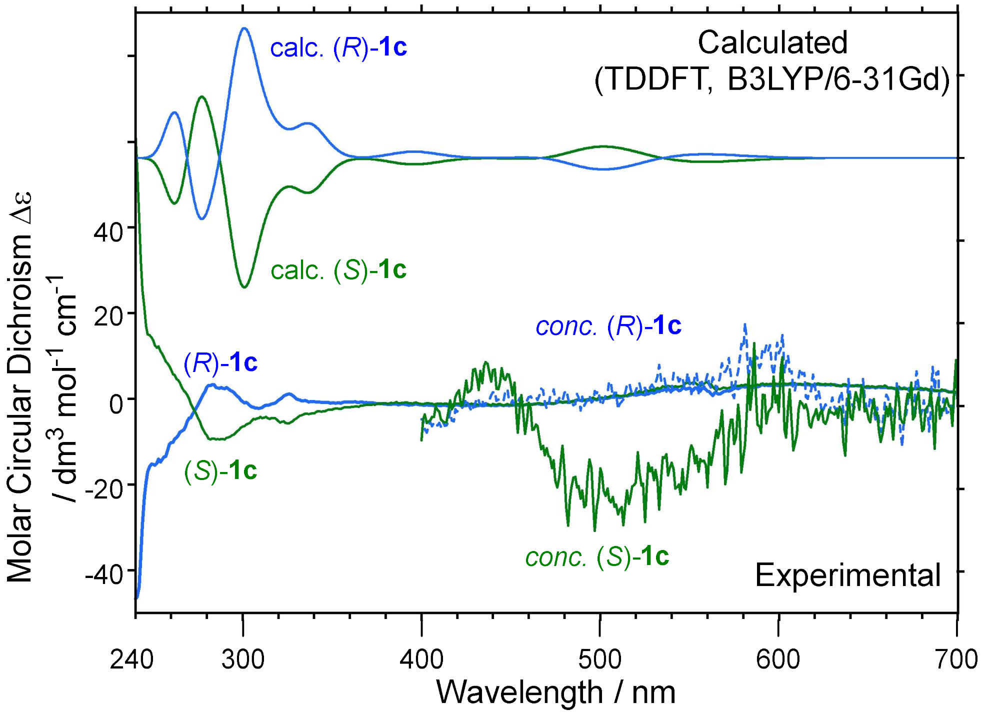

Figure SI-11 CD spectra of optically pure **1c** and the corresponding simulated spectra obtained at the B3LYP/6-31G(d) level.

§4 Structural optimization and TDDFT studies for **1b**

For structural optimization, the initial geometry was taken from the atomic coordinates determined by single-crystal X-ray diffraction. The optimized coordinates of **1b** are as follows.

O	-0.05132200	0.08372800	-1.54385300
O	4.70365000	-0.19416100	0.16662200
N	-0.31759400	-0.05552400	0.95138600
N	-2.08091000	-0.70230700	-0.49205200
N	-1.55559000	1.58838400	-0.20910000
N	-0.20536600	2.21676000	1.64470000
N	-3.62946500	0.96069200	-1.19224000
N	-1.22231500	-2.25095200	1.09419400
C	-2.10452000	-1.91538200	0.14057300
C	-0.39707100	-1.29189700	1.53752300
C	-3.29774800	-0.32422100	-0.99296000
C	-1.09444600	2.51737000	0.68528500
C	2.68467400	-1.05465300	-0.87568300
C	-3.39343000	-2.50531500	-0.19778600
C	0.28619800	-1.16224500	2.81851700
C	0.10656300	0.92331900	1.81151800
C	-4.13932000	-1.51102600	-0.90707700
C	2.35141200	0.32149100	-1.30383500
C	-1.98422800	3.66317000	0.55293100
C	-3.04355400	3.27486300	-0.32770900
C	-2.78864300	1.89588200	-0.72319300
C	1.07701900	-2.32824100	-2.35212900
H	0.86204300	-1.43558500	-2.91961800
C	2.00384900	-2.26755800	-1.27534400
C	0.60187700	0.22208400	2.98952000
C	1.09877300	0.81384300	-1.68409100
C	-3.97307900	-3.73600700	0.11896200
H	-3.41082700	-4.48203900	0.67189300
C	3.43250200	1.26785900	-1.27715700
C	0.56045100	-2.08347600	3.83155400
H	0.30458100	-3.13089600	3.70606000
C	1.19007300	0.67576300	4.17212000
H	1.41385700	1.72945100	4.30641500

C	2.34066100	-3.50798600	-0.63206400
C	3.81626800	-1.21105300	-0.07681700
C	4.62393100	0.95856100	-0.57752700
C	-5.45929900	-1.75548000	-1.29326000
H	-6.02995900	-0.99358600	-1.81519500
C	0.98670800	2.10036700	-2.27666300
H	0.00604500	2.39694600	-2.63554700
C	3.32432400	2.56785300	-1.86023600
C	2.06669800	2.93789800	-2.40587300
H	1.95791600	3.90860400	-2.88258200
C	-6.01416900	-2.99704800	-0.99476800
H	-7.03370400	-3.21536800	-1.30007500
C	-4.10785100	4.14327900	-0.58332700
H	-4.92013400	3.84037400	-1.23679800
C	-5.27992900	-3.97558000	-0.29718500
H	-5.74570700	-4.93194400	-0.07641700
C	-1.99717700	4.91663600	1.16975600
H	-1.20156300	5.20230500	1.85084300
C	4.15440900	-2.42814100	0.55424500
H	5.01678500	-2.44066500	1.21268000
C	0.46416900	-3.51116500	-2.70864100
H	-0.22874900	-3.52022500	-3.54591900
C	1.48288700	-0.25841500	5.16229000
H	1.95588700	0.06865500	6.08401500
C	1.17171600	-1.62131500	4.99411700
H	1.40976300	-2.32236300	5.78923800
C	5.69850000	1.82318300	-0.53477000
H	6.58521000	1.53932900	0.02222300
C	3.39702700	-3.54373400	0.31717600
H	3.63426400	-4.48425500	0.80780100
C	0.73350500	-4.70845000	-2.01009000
H	0.23083100	-5.62993800	-2.29093500
C	1.66429800	-4.70106900	-0.99736400
H	1.92270500	-5.62008300	-0.47608900
C	-3.04993100	5.78127000	0.88337400
H	-3.07256600	6.76719400	1.33914300
C	-4.09286000	5.39903200	0.01730700
H	-4.90198000	6.09672700	-0.17985200

C	5.60496100	3.06902100	-1.18859300
H	6.45344100	3.74698800	-1.15925400
C	4.44164300	3.44216900	-1.82790300
H	4.35349300	4.42158600	-2.29055800
B	-0.91772300	0.23603200	-0.39803500

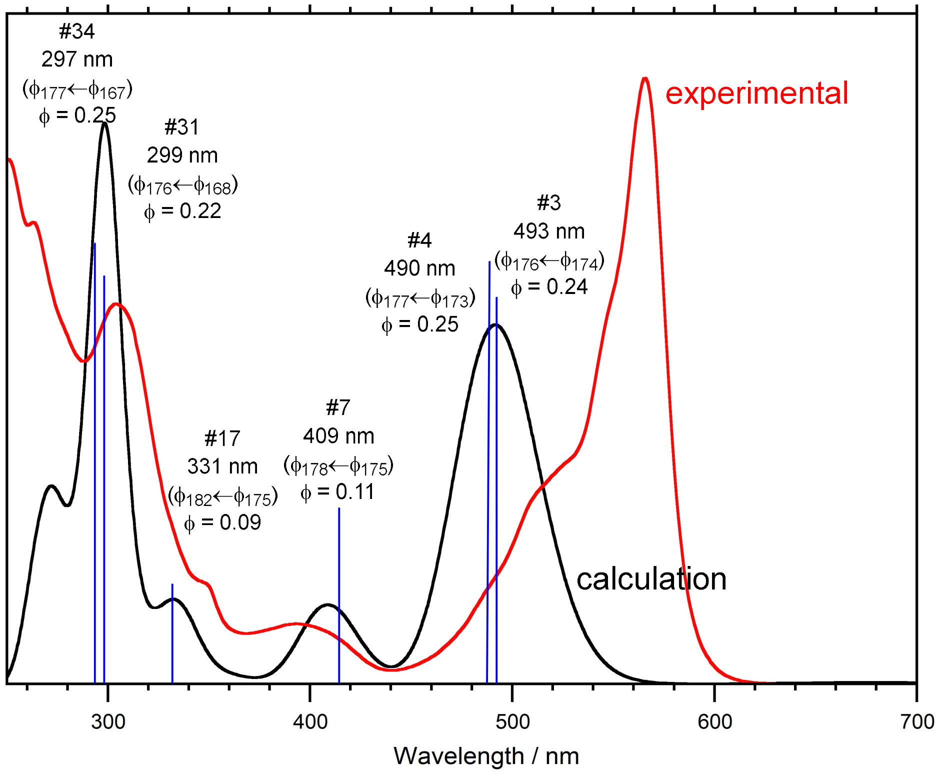

Figure SI-12 Absorption spectra of **1b** and TDDFT simulation.

§5 Dihedral angle dependence of the CD behavior of dimethoxy BINOL.

The dihedral angle dependence of the CD behavior of dimethoxy BINOL was investigated as follows.

Structural optimizations were carried out by constraining the torsion angle ∠C2–C1–C1′–C2′ from 0° to 180° in 10° increments. The definition of the angle for the (*S*)-isomer is shown in the following figure. The dihedral angle θ of BINOL was evaluated as the angle between the least-squares planes defined by ten carbon atoms of the naphthalene rings. For example, for a structure with ∠C2–C1–C1′–C2′ constrained to 110°, the resulting θ was 107.09°. Using these optimized coordinates, TDDFT calculations were performed.

The most stable structure was obtained starting from initial coordinates with acute and/or obtuse angles, and the dihedral angle of the optimized structure was θ = 89.1°

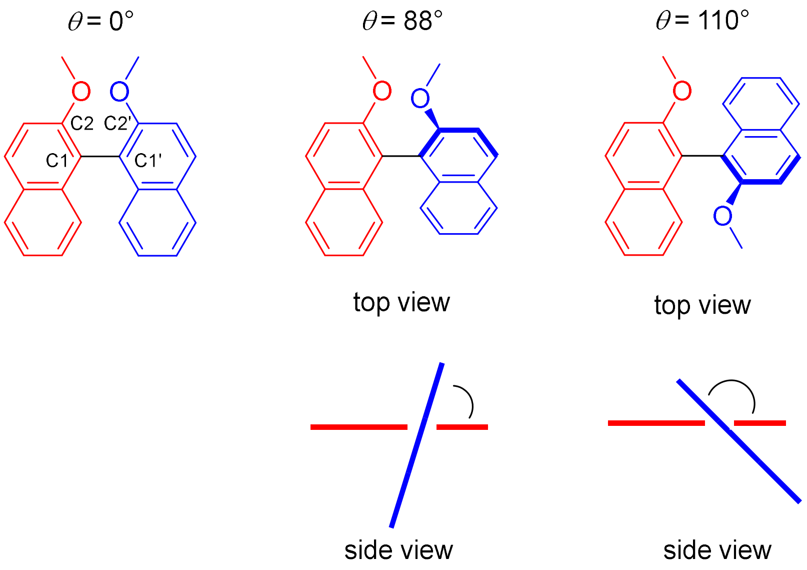

Figure SI-13 Definition of the torsion angles (dihedral) of dimethoxy BINOL.

5-1 Optimized coordinate of dimethoxy BINOL with θ = 47.22° (∠C2-C1-C1'-C2' = 0°).

O	0.64880800	1.06644000	2.57710900
O	-0.64880800	-1.06644000	2.57710900
C	0.00000000	1.56453100	1.48122700
C	0.00000000	0.75396300	0.32575600
C	-0.74529500	1.36350500	-0.79247100
C	-0.32711200	2.94638600	1.46042500
H	-0.18945100	3.53707600	2.35718400
C	-1.00680200	2.77455700	-0.84160800
C	0.00000000	-0.75396300	0.32575600
C	-0.76946500	3.54932000	0.31617900

H	-0.98285600	4.61547400	0.30122200
C	1.32026400	-0.60487800	-1.85756700
H	1.28288700	0.47435500	-1.82018200
C	-1.61581800	3.36869400	-1.98031600
H	-1.75115700	4.44812900	-1.98397800
C	-1.32026400	0.60487800	-1.85756700
H	-1.28288700	-0.47435500	-1.82018200
C	0.00000000	-1.56453100	1.48122700
C	0.74529500	-1.36350500	-0.79247100
C	-2.06757800	2.60065800	-3.02546300
H	-2.54390600	3.05769900	-3.88838400
C	1.00680200	-2.77455700	-0.84160800
C	-1.95180800	1.19634200	-2.93027100
H	-2.37605100	0.56626900	-3.70787500
C	0.32711200	-2.94638600	1.46042500
H	0.18945100	-3.53707600	2.35718400
C	-0.64292900	-1.77968800	3.79839200
H	-1.25255200	-2.69243200	3.74709000
H	-1.08463500	-1.10626500	4.53638900
H	0.37423000	-2.04416400	4.11597100
C	0.76946500	-3.54932000	0.31617900
H	0.98285600	-4.61547400	0.30122200
C	1.61581800	-3.36869400	-1.98031600
H	1.75115700	-4.44812900	-1.98397800
C	1.95180800	-1.19634200	-2.93027100
H	2.37605100	-0.56626900	-3.70787500
C	2.06757800	-2.60065800	-3.02546300
H	2.54390600	-3.05769900	-3.88838400
C	0.64292900	1.77968800	3.79839200
H	1.08463500	1.10626500	4.53638900
H	1.25255200	2.69243200	3.74709000
H	-0.37423000	2.04416400	4.11597100

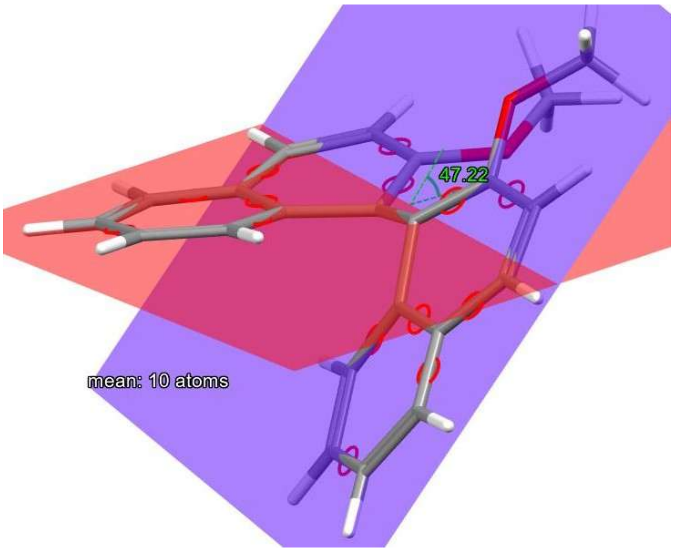

Figure SI-14 Optimized structure with the torsion angle ∠C2–C1–C1′–C2′ constrained to 0°. The dihedral angle between the naphthalene rings is $\theta = 47.22°$.

5-2 The coordinates of the most stable dimethoxy BINOL. The dihedral angle is $\theta = 89.06°$.

O	1.63810500	0.66874000	1.94163600
O	-1.63810500	-0.66874000	1.94163600
C	0.84316200	1.43609300	1.13680500
C	-0.00128800	0.74787600	0.27002300
C	-0.86177400	1.48587000	-0.59750400
C	0.86237400	2.85396900	1.16377400
H	1.52489700	3.37935600	1.84179700
C	-0.84316200	2.92084700	-0.56925600
C	0.00128800	-0.74787600	0.27002300
C	0.03632800	3.57092900	0.32915600
H	0.05491400	4.65804300	0.35434500
C	1.75302400	-0.84726800	-1.50731400
H	1.77690400	0.23700200	-1.54015600
C	-1.70154800	3.64870400	-1.43729100
H	-1.67412300	4.73573700	-1.40243400
C	-1.75302400	0.84726800	-1.50731400
H	-1.77690400	-0.23700200	-1.54015600
C	-0.84316200	-1.43609300	1.13680500
C	0.86177400	-1.48587000	-0.59750400
C	-2.54913900	2.99869300	-2.30317600
H	-3.20092900	3.56522700	-2.96271200

C	0.84316200	-2.92084700	-0.56925600
C	-2.57100100	1.58224800	-2.33444800
H	-3.24190300	1.07150100	-3.02048200
C	-0.86237400	-2.85396900	1.16377400
H	-1.52489700	-3.37935600	1.84179700
C	-2.52762600	-1.30785400	2.84027800
H	-3.26359500	-1.93035300	2.31376600
H	-3.04929200	-0.50310100	3.36199000
H	-1.99290800	-1.92533400	3.57452600
C	-0.03632800	-3.57092900	0.32915600
H	-0.05491400	-4.65804300	0.35434500
C	1.70154800	-3.64870400	-1.43729100
H	1.67412300	-4.73573700	-1.40243400
C	2.57100100	-1.58224800	-2.33444800
H	3.24190300	-1.07150100	-3.02048200
C	2.54913900	-2.99869300	-2.30317600
H	3.20092900	-3.56522700	-2.96271200
C	2.52762600	1.30785400	2.84027800
H	3.04929200	0.50310100	3.36199000
H	3.26359500	1.93035300	2.31376600
H	1.99290800	1.92533400	3.57452600

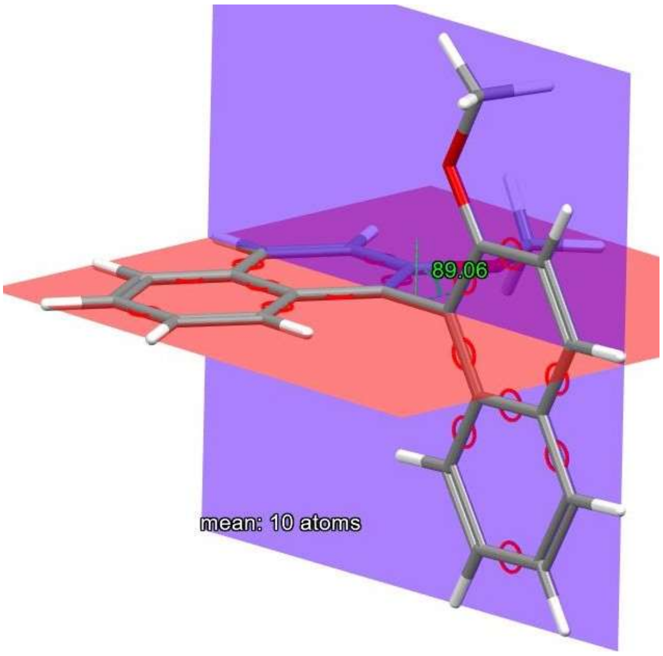

Figure SI-15 Optimized most stable structure with $\theta = 89.06°$.

5-3 Optimized coordinate of dimethoxy BINOL with θ = 107.09°.

This angle is close to that observed in the structure of **1c.**

O	1.90164600	0.66384100	1.62695600
O	-1.90164600	-0.66384100	1.62695600
C	0.99730100	1.43335300	0.94908600
C	0.00246600	0.74761900	0.25408200
C	-0.97031800	1.49546500	-0.48024500
C	1.04125100	2.85070700	0.95811600
H	1.81552100	3.36990200	1.51083800
C	-0.92875300	2.92996100	-0.46433400
C	-0.00246600	-0.74761900	0.25408200
C	0.09499800	3.57409600	0.27067000
H	0.13018500	4.66096800	0.28425700
C	1.98992500	-0.86993000	-1.25370500
H	2.02388500	0.21332000	-1.29506600
C	-1.90164600	3.66867700	-1.19123500
H	-1.85464700	4.75521000	-1.16286700
C	-1.98992500	0.86993000	-1.25370500
H	-2.02388500	-0.21332000	-1.29506600
C	-0.99730100	-1.43335300	0.94908600
C	0.97031800	-1.49546500	-0.48024500
C	-2.87763100	3.02999900	-1.91872200
H	-3.61553800	3.60416600	-2.47243200
C	0.92875300	-2.92996100	-0.46433400
C	-2.91367100	1.61413100	-1.95085400
H	-3.67900000	1.11054000	-2.53601900
C	-1.04125100	-2.85070700	0.95811600
H	-1.81552100	-3.36990200	1.51083800
C	-2.95137400	-1.29652800	2.33775600
H	-3.59259000	-1.89328600	1.67520700
H	-3.54186600	-0.48804900	2.77297100
H	-2.57019200	-1.93784400	3.14378300
C	-0.09499800	-3.57409600	0.27067000
H	-0.13018500	-4.66096800	0.28425700
C	1.90164600	-3.66867700	-1.19123500
H	1.85464700	-4.75521000	-1.16286700

C	2.91367100	-1.61413100	-1.95085400
H	3.67900000	-1.11054000	-2.53601900
C	2.87763100	-3.02999900	-1.91872200
H	3.61553800	-3.60416600	-2.47243200
C	2.95137400	1.29652800	2.33775600
H	3.54186600	0.48804900	2.77297100
H	3.59259000	1.89328600	1.67520700
H	2.57019200	1.93784400	3.14378300

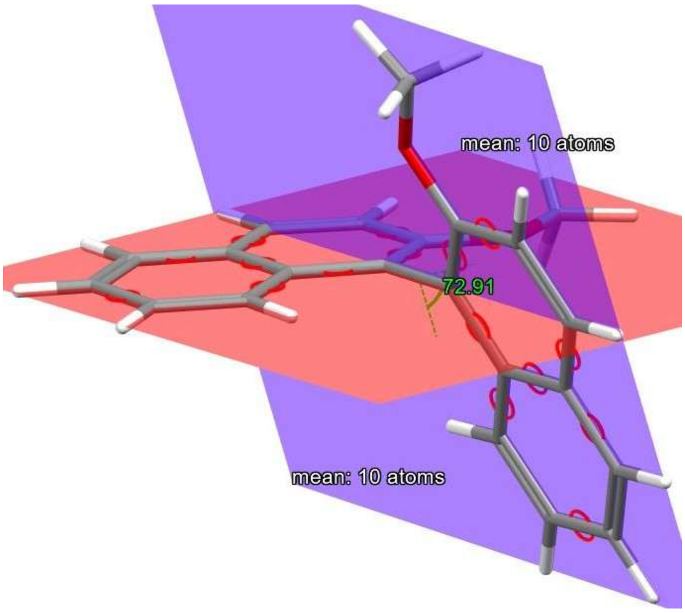

Figure SI-16 Optimized structure with the torsion angle ∠C2–C1–C1′–C2′ constrained to 110°. The dihedral angle between the naphthalene rings is θ = 107.09°.

5-4 Optimized coordinate of dimethoxy BINOL with θ = 107.09°. The dihedral 135.49

O	-2.22500800	0.94381000	-1.18855100
O	2.22500800	-0.94381000	-1.18855100
C	-1.13850800	1.53250200	-0.58128700
C	0.03648000	0.75487000	-0.58128500
C	1.16089000	1.44079300	0.08107300
C	-1.16089000	2.93427200	-0.36580500
H	-2.08668900	3.47957700	-0.49922000
C	1.18133500	2.87677500	0.17608600
C	-0.03648000	-0.75487000	-0.58128500
C	-0.00623200	3.60308100	-0.07160800

H	-0.00266200	4.68565700	0.02853400
C	-2.22074900	-0.79363300	0.78403200
H	-2.15948300	0.26669000	0.97795300
C	2.32490500	3.56500200	0.66697900
H	2.30941000	4.65262300	0.65719100
C	2.22074900	0.79363300	0.78403200
H	2.15948300	-0.26669000	0.97795300
C	1.13850800	-1.53250200	-0.58128700
C	-1.16089000	-1.44079300	0.08107300
C	3.38910200	2.88273400	1.20231300
H	4.25449900	3.41160300	1.59179400
C	-1.18133500	-2.87677500	0.17608600
C	3.29058400	1.47917100	1.31827700
H	4.05508100	0.92836400	1.86085200
C	1.16089000	-2.93427200	-0.36580500
H	2.08668900	-3.47957700	-0.49922000
C	3.47958600	-1.59845400	-1.20426400
H	3.79903800	-1.90591000	-0.19906300
H	4.18896700	-0.86520700	-1.59275000
H	3.47397500	-2.47658800	-1.86394500
C	0.00623200	-3.60308100	-0.07160800
H	0.00266200	-4.68565700	0.02853400
C	-2.32490500	-3.56500200	0.66697900
H	-2.30941000	-4.65262300	0.65719100
C	-3.29058400	-1.47917100	1.31827700
H	-4.05508100	-0.92836400	1.86085200
C	-3.38910200	-2.88273400	1.20231300
H	-4.25449900	-3.41160300	1.59179400
C	-3.47958600	1.59845400	-1.20426400
H	-4.18896700	0.86520700	-1.59275000
H	-3.79903800	1.90591000	-0.19906300
H	-3.47397500	2.47658800	-1.86394500

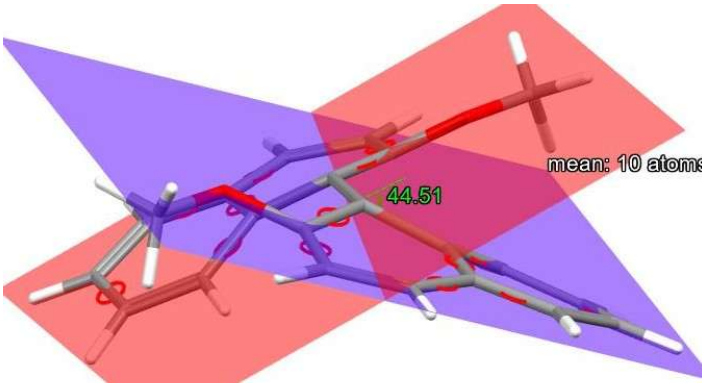

Figure SI-17 Optimized structure with the torsion angle ∠C2–C1–C1′–C2′ constrained to 180°. The dihedral angle between the naphthalene rings is θ = 135.49°.

5-5 Dihedral angle–dependent CD spectra of dimethoxy BINOL

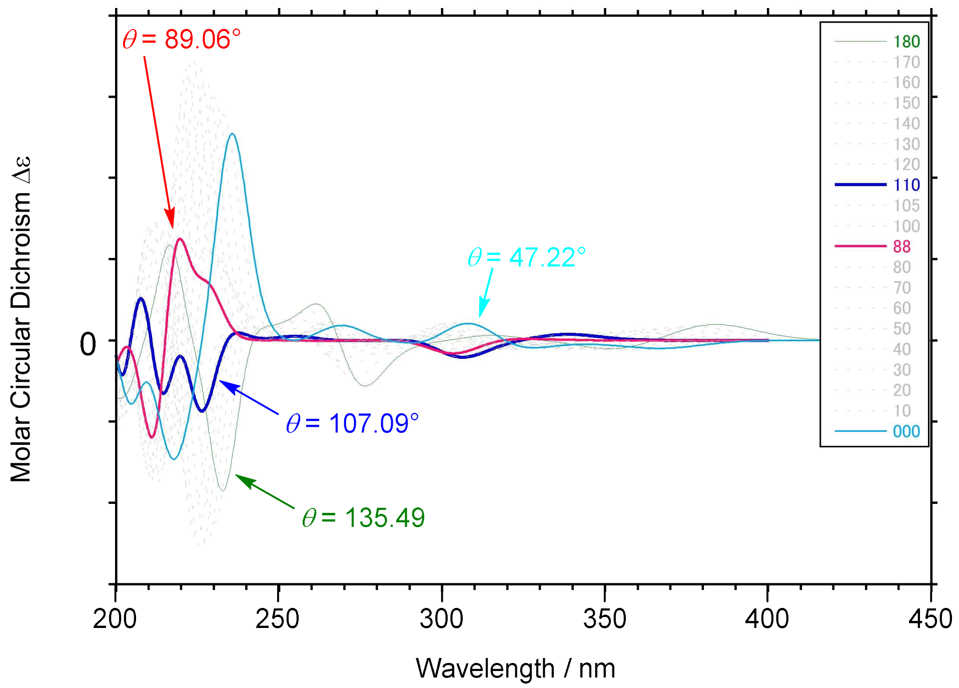

Figure SI-18 Simulated CD spectra of dimethoxy BINOL as a function of the dihedral angle between the naphthalene rings, defined by ten carbon atoms, calculated at the B3LYP/6-31G(d) level. The most stable structure has a dihedral angle of θ = 88°. The optimized coordinates of dimethoxy BINOL at θ = 0°, 88°, 110°, and 180° are shown below. We also optimized the structures and carried out TDDFT calculations at θ = 20°, 30°, 40°, 50°, 60°, 70°, 80°, 90°, 100°, 120°, 130°, 140°, 150°, and 170°; however, these results are omitted. The TDDFT results for these angles are plotted as pale dotted lines in the above figure.

§6 Single Crystal Diffraction Studies

6-1 **1b**

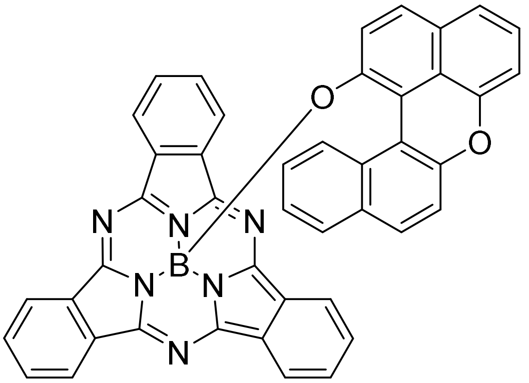

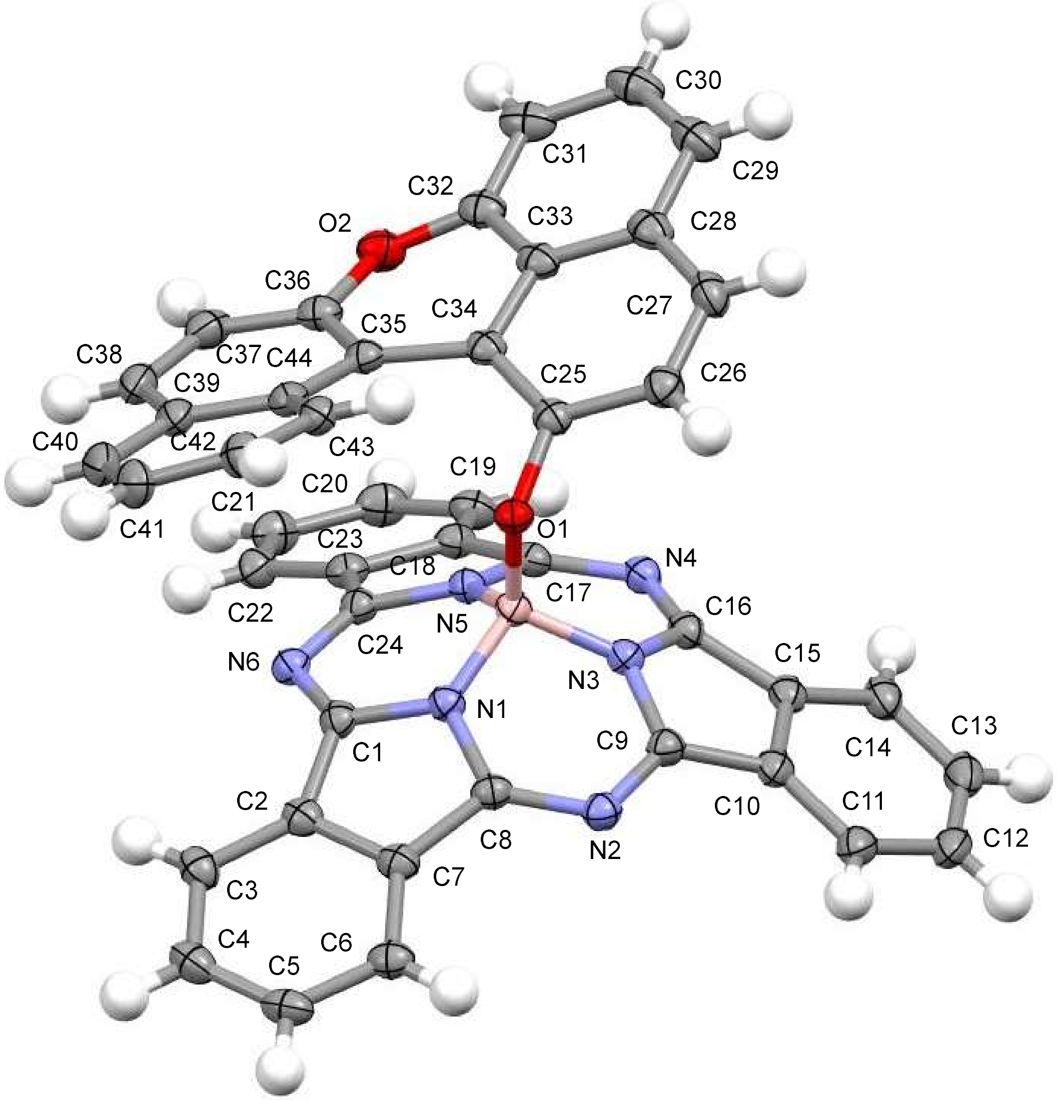

Figure SI-19 Molecular structure of **1b**. Thermal ellipsoids are shown at the 50% probability level.

Table 6-1-1 Crystal data and structure refinement for **1b**.

Identification code	**1b**
Empirical formula	$C_{44}H_{23}BN_6O_2$
Formula weight	678.49
Temperature/K	100
Crystal system	monoclinic
Space group	$P2_1/c$
a/Å	15.2940(3)
b/Å	10.1912(2)
c/Å	21.3972(4)
α/°	90
β/°	106.137(2)
γ/°	90
Volume/Å^3	3203.66(11)
Z	4
ρ_{calc}g/cm^3	1.407
μ/mm^{-1}	0.706
F(000)	1400.0
Crystal size/mm^3	0.6 × 0.2 × 0.15
Radiation	Cu Kα (λ = 1.54184)
2Θ range for data collection/°	6.016 to 142.444
Index ranges	-18 ≤ h ≤ 12, -12 ≤ k ≤ 12, -25 ≤ l ≤ 25
Reflections collected	17876
Independent reflections	6039 [R_{int} = 0.0418, R_{sigma} = 0.0430]
Data/restraints/parameters	6039/0/478
Goodness-of-fit on F^2	1.038
Final R indexes [I>=2σ (I)]	R_1 = 0.0440, wR_2 = 0.1207
Final R indexes [all data]	R_1 = 0.0503, wR_2 = 0.1253
Largest diff. peak/hole / e Å^{-3}	0.30/-0.29

Table 6-1-2 Fractional Atomic Coordinates (×10^4) and Equivalent Isotropic Displacement Parameters (Å^2×10^3) for dt01_auto. U_{eq} is defined as 1/3 of the trace of the orthogonalised U_{IJ} tensor.

**Atom**	***x***	***y***	***z***	**U(eq)**
O1	3127.9(6)	6799.8(9)	6558.6(5)	22.0(2)
O2	985.9(7)	3352.9(11)	6901.4(5)	30.8(3)

Table 6-1-2 Fractional Atomic Coordinates (×10^4) and Equivalent Isotropic Displacement Parameters (Å^2×10^3) for dt01_auto. U_{eq} is defined as 1/3 of the trace of the orthogonalised U_{IJ} tensor.

**Atom**	***x***	***y***	***z***	**U(eq)**
N5	2644.9(8)	4985.1(11)	5747.0(6)	20.7(2)
N1	4075.5(8)	5974.0(11)	5899.5(6)	21.2(3)
N3	2744.0(8)	7124.0(11)	5360.8(6)	21.7(3)
N4	1355.3(8)	5932.9(12)	4994.4(6)	23.7(3)
N2	4177.7(8)	7851.0(12)	5266.8(6)	23.8(3)
N6	3979.8(8)	3670.6(12)	6052.4(6)	22.7(3)
C1	4463.6(10)	4759.4(14)	6027.0(7)	22.5(3)
C24	3067.7(9)	3797.5(14)	5867.8(7)	21.4(3)
C8	4563.1(10)	6817.4(14)	5633.9(7)	22.3(3)
C16	1870.1(10)	6996.7(14)	4974.8(7)	23.1(3)
C35	2431.4(10)	4491.5(14)	7145.3(7)	22.8(3)
C2	5387.6(10)	4914.8(15)	5971.9(7)	24.0(3)
C23	2353.2(10)	2826.2(14)	5625.7(7)	23.0(3)
C17	1771.3(9)	4913.5(14)	5355.2(7)	21.6(3)
C7	5445.5(10)	6199.1(15)	5723.3(7)	23.7(3)
C34	1942.0(10)	5715.9(14)	6906.0(7)	23.0(3)
C15	1757.3(10)	8009.3(14)	4482.3(7)	25.1(3)
C10	2626.6(10)	8590.6(15)	4560.8(7)	25.7(3)
C9	3264.5(10)	7935.5(14)	5105.2(7)	23.3(3)
C43	3926.4(10)	5518.7(15)	7734.8(7)	26.1(3)
C44	3387.0(10)	4406.5(15)	7480.1(7)	24.5(3)
C18	1542.8(10)	3527.4(14)	5309.7(7)	23.4(3)
C25	2295.5(10)	6821.5(14)	6686.4(7)	22.6(3)
C3	6128.4(10)	4064.3(16)	6093.1(7)	27.5(3)
C33	999.7(10)	5727.8(15)	6876.5(7)	25.5(3)
C22	2356.7(11)	1463.3(15)	5624.8(7)	27.1(3)
C19	746.5(10)	2870.8(15)	4992.2(8)	28.3(3)
C39	3800.6(11)	3146.5(15)	7607.5(7)	27.8(3)
C36	1925.3(10)	3350.6(15)	7091.7(7)	26.6(3)
C32	544.1(10)	4534.6(16)	6912.2(7)	27.8(3)
C6	6243.9(10)	6625.5(16)	5593.6(7)	27.5(3)
C26	1788.5(10)	8004.0(15)	6576.3(7)	27.8(3)

Table 6-1-2 Fractional Atomic Coordinates (×10^4) and Equivalent Isotropic Displacement Parameters (Å^2×10^3) for dt01_auto. U_{eq} is defined as 1/3 of the trace of the orthogonalised U_{IJ} tensor.

**Atom**	***x***	***y***	***z***	**U(eq)**
C28	483.8(11)	6910.6(16)	6771.6(7)	29.2(3)
C27	926.9(11)	8063.8(16)	6642.6(8)	31.5(4)
C5	6981.2(10)	5782.2(17)	5739.1(7)	30.4(3)
C11	2750.3(12)	9552.4(16)	4128.3(8)	30.9(3)
C4	6924.8(10)	4524.3(17)	5986.8(8)	30.5(3)
C14	1007.0(11)	8392.8(16)	3975.3(7)	29.9(3)
C37	2327.5(12)	2103.6(16)	7226.8(8)	31.3(4)
C42	4834.8(11)	5396.3(17)	8059.6(8)	31.7(4)
C20	761.2(11)	1515.8(16)	5012.1(8)	31.8(4)
C21	1549.6(11)	822.2(15)	5325.8(8)	31.1(3)
C31	-347.7(11)	4507.7(18)	6919.7(8)	32.9(4)
C38	3251.4(12)	2006.8(16)	7457.5(8)	31.7(4)
C41	5257.9(11)	4158.9(18)	8122.5(8)	35.2(4)
C40	4752.0(11)	3060.9(17)	7907.6(8)	33.1(4)
C13	1143.1(12)	9361.3(17)	3560.3(8)	35.3(4)
C12	1994.0(12)	9934.8(17)	3635.2(8)	35.9(4)
C30	-827.9(11)	5695.7(19)	6866.5(8)	36.2(4)
C29	-436.7(11)	6859.9(18)	6781.1(8)	35.3(4)
B1	3137.0(11)	6253.7(16)	5933.2(8)	21.3(3)

Table 6-1-3 Anisotropic Displacement Parameters (Å^2×10^3) for dt01_auto. The Anisotropic displacement factor exponent takes the form: $-2\pi^2[h^2a^{*2}U_{11}+2hka^*b^*U_{12}+\ldots]$.

**Atom**	U_{11}	U_{22}	U_{33}	U_{23}	U_{13}	U_{12}
O1	22.8(5)	22.9(5)	20.6(5)	-0.7(4)	6.6(4)	-0.2(4)
O2	28.7(6)	31.4(6)	33.8(6)	-2.3(5)	11.6(5)	-4.5(5)
N5	22.3(6)	20.8(6)	19.5(6)	0.4(5)	6.7(5)	1.1(5)
N1	21.9(6)	20.5(6)	21.4(6)	-0.3(5)	6.2(5)	0.7(5)
N3	22.6(6)	21.1(6)	21.9(6)	0.1(5)	7.1(5)	2.0(5)
N4	22.2(6)	25.8(6)	23.7(6)	-1.4(5)	7.2(5)	2.6(5)
N2	27.1(6)	22.9(6)	22.6(6)	-0.4(5)	8.9(5)	-0.4(5)
N6	23.6(6)	23.8(6)	20.6(6)	0.6(5)	5.9(5)	0.8(5)
C1	25.6(7)	22.9(7)	18.1(7)	0.1(5)	4.8(5)	2.3(6)

Table 6-1-3 Anisotropic Displacement Parameters ($Å^2 \times 10^3$) for dt01_auto. The Anisotropic displacement factor exponent takes the form: $-2\pi^2[h^2a^{*2}U_{11}+2hka^*b^*U_{12}+\ldots]$.

Atom	U_{11}	U_{22}	U_{33}	U_{23}	U_{13}	U_{12}
C24	24.6(7)	21.1(7)	19.2(7)	0.0(5)	7.2(5)	0.9(6)
C8	23.3(7)	23.7(7)	20.4(7)	-1.9(5)	7.1(5)	-2.5(6)
C16	22.9(7)	24.8(7)	21.6(7)	-1.8(5)	6.3(6)	4.1(5)
C35	26.4(7)	25.3(7)	18.0(6)	0.5(5)	8.5(5)	0.5(6)
C2	23.1(7)	28.2(7)	19.5(7)	-3.4(6)	4.0(5)	0.4(6)
C23	24.4(7)	25.7(7)	20.4(7)	-0.8(6)	8.7(6)	-1.8(6)
C17	20.1(6)	25.2(7)	20.6(7)	-2.3(5)	7.7(5)	0.3(5)
C7	23.2(7)	27.2(7)	20.3(7)	-3.3(6)	5.5(5)	-0.6(6)
C34	24.5(7)	27.4(7)	17.6(7)	-3.2(6)	6.6(5)	1.0(6)
C15	29.3(8)	24.3(7)	22.4(7)	-0.8(6)	8.2(6)	6.5(6)
C10	31.6(8)	23.4(7)	23.4(7)	-0.3(6)	9.9(6)	5.7(6)
C9	27.4(7)	21.6(7)	22.7(7)	0.4(5)	9.7(6)	0.9(6)
C43	25.8(7)	30.4(8)	22.2(7)	4.0(6)	7.0(6)	3.9(6)
C44	27.6(7)	28.1(8)	19.4(7)	3.0(6)	9.4(6)	3.4(6)
C18	24.7(7)	24.7(7)	22.7(7)	-2.3(6)	9.8(6)	-0.3(6)
C25	23.2(7)	25.3(7)	18.4(7)	-2.7(5)	4.3(5)	1.4(6)
C3	26.6(8)	28.2(8)	26.9(8)	-4.5(6)	6.0(6)	4.2(6)
C33	25.0(7)	33.0(8)	18.6(7)	-2.8(6)	6.2(6)	0.9(6)
C22	32.6(8)	23.5(7)	26.2(7)	-0.6(6)	10.1(6)	0.5(6)
C19	24.9(7)	31.2(8)	29.9(8)	-3.6(6)	9.5(6)	-2.2(6)
C39	34.1(8)	29.3(8)	21.6(7)	5.3(6)	10.4(6)	6.3(6)
C36	28.4(8)	31.9(8)	21.5(7)	-0.7(6)	10.6(6)	-1.4(6)
C32	28.8(8)	34.3(8)	21.0(7)	-4.2(6)	8.0(6)	-1.6(6)
C6	27.7(8)	33.0(8)	22.6(7)	-3.0(6)	8.3(6)	-2.9(6)
C26	31.8(8)	25.4(8)	26.2(8)	-0.1(6)	8.2(6)	2.6(6)
C28	28.3(8)	36.8(9)	22.6(7)	-4.4(6)	7.0(6)	4.8(6)
C27	33.1(8)	29.9(8)	30.3(8)	-2.0(6)	6.6(6)	10.7(7)
C5	23.7(7)	43.4(9)	26.5(8)	-6.4(7)	10.9(6)	-2.7(7)
C11	40.2(9)	27.8(8)	28.7(8)	3.9(6)	15.9(7)	6.5(7)
C4	23.0(7)	39.1(9)	28.6(8)	-8.1(7)	6.1(6)	4.4(6)
C14	32.1(8)	32.0(8)	24.7(8)	-0.9(6)	6.4(6)	10.4(6)
C37	42.8(9)	26.2(8)	28.8(8)	1.5(6)	16.5(7)	-3.5(7)

Table 6-1-3 Anisotropic Displacement Parameters (Å^2×10^3) for dt01_auto. The Anisotropic displacement factor exponent takes the form: $-2\pi^2[h^2a^{*2}U_{11}+2hka^*b^*U_{12}+\ldots]$.

Atom	U_{11}	U_{22}	U_{33}	U_{23}	U_{13}	U_{12}
C42	28.2(8)	37.7(9)	27.1(8)	3.2(7)	4.3(6)	0.6(7)
C20	31.9(8)	30.3(8)	34.7(8)	-6.1(7)	12.0(7)	-9.3(7)
C21	39.1(9)	22.3(7)	34.8(8)	-4.8(6)	15.3(7)	-5.6(6)
C31	27.6(8)	45.4(10)	26.6(8)	-9.4(7)	9.0(6)	-8.5(7)
C38	43.4(9)	26.9(8)	28.1(8)	6.6(6)	15.4(7)	7.5(7)
C41	28.1(8)	44.3(10)	30.2(8)	7.3(7)	3.0(6)	8.1(7)
C40	35.1(9)	37.2(9)	27.4(8)	8.9(7)	9.2(7)	13.4(7)
C13	41.6(9)	39.7(9)	24.3(8)	4.5(7)	8.6(7)	17.4(8)
C12	50.5(10)	34.8(9)	26.4(8)	9.7(7)	17.3(7)	14.0(8)
C30	22.2(7)	58.2(11)	28.5(8)	-12.0(8)	7.3(6)	-1.6(7)
C29	26.5(8)	45.8(10)	32.7(9)	-7.6(7)	6.6(7)	6.9(7)
B1	22.3(8)	20.9(8)	20.6(8)	0.5(6)	6.0(6)	0.6(6)

Table 6-1-4 Bond Lengths for **1b**.

Atom	Atom	Length/Å	Atom	Atom	Length/Å
O1	C25	1.3746(17)	C34	C33	1.4250(19)
O1	B1	1.4530(18)	C15	C10	1.422(2)
O2	C36	1.3803(18)	C15	C14	1.398(2)
O2	C32	1.3842(19)	C10	C9	1.457(2)
N5	C24	1.3627(18)	C10	C11	1.396(2)
N5	C17	1.3678(18)	C43	C44	1.419(2)
N5	B1	1.4933(19)	C43	C42	1.375(2)
N1	C1	1.3671(18)	C44	C39	1.424(2)
N1	C8	1.3614(18)	C18	C19	1.390(2)
N1	B1	1.4845(19)	C25	C26	1.417(2)
N3	C16	1.3682(18)	C3	C4	1.382(2)
N3	C9	1.3634(19)	C33	C32	1.414(2)
N3	B1	1.4961(19)	C33	C28	1.424(2)
N4	C16	1.3474(19)	C22	C21	1.386(2)
N4	C17	1.3443(19)	C19	C20	1.382(2)
N2	C8	1.3479(19)	C39	C38	1.417(2)
N2	C9	1.3450(19)	C39	C40	1.420(2)

Table 6-1-4 Bond Lengths for **1b**.

Atom	Atom	Length/Å	Atom	Atom	Length/Å
N6	C1	1.3431(19)	C36	C37	1.406(2)
N6	C24	1.3462(18)	C32	C31	1.369(2)
C1	C2	1.4591(19)	C6	C5	1.383(2)
C24	C23	1.459(2)	C26	C27	1.365(2)
C8	C7	1.453(2)	C28	C27	1.421(2)
C16	C15	1.451(2)	C28	C29	1.415(2)
C35	C34	1.472(2)	C5	C4	1.399(2)
C35	C44	1.440(2)	C11	C12	1.387(2)
C35	C36	1.384(2)	C14	C13	1.382(2)
C2	C7	1.425(2)	C37	C38	1.365(2)
C2	C3	1.392(2)	C42	C41	1.407(2)
C23	C18	1.428(2)	C20	C21	1.398(2)
C23	C22	1.389(2)	C31	C30	1.404(3)
C17	C18	1.452(2)	C41	C40	1.366(2)
C7	C6	1.395(2)	C13	C12	1.395(3)
C34	C25	1.387(2)	C30	C29	1.364(3)

Table 6-1-5 Bond Angles for **1b**.

Atom	Atom	Atom	Angle/°	Atom	Atom	Atom	Angle/°
C25	O1	B1	115.84(11)	C42	C43	C44	121.30(15)
C36	O2	C32	118.00(12)	C43	C44	C35	122.98(13)
C24	N5	C17	113.58(12)	C43	C44	C39	117.84(14)
C24	N5	B1	122.75(12)	C39	C44	C35	119.00(14)
C17	N5	B1	122.94(12)	C23	C18	C17	107.11(12)
C1	N1	B1	122.43(12)	C19	C18	C23	121.19(14)
C8	N1	C1	113.39(12)	C19	C18	C17	131.52(14)
C8	N1	B1	123.24(12)	O1	C25	C34	121.60(12)
C16	N3	B1	122.73(12)	O1	C25	C26	118.36(13)
C9	N3	C16	113.37(12)	C34	C25	C26	120.03(13)
C9	N3	B1	122.78(12)	C4	C3	C2	117.84(15)
C17	N4	C16	116.89(12)	C32	C33	C34	119.85(14)
C9	N2	C8	117.04(12)	C32	C33	C28	118.47(14)
C1	N6	C24	116.51(12)	C28	C33	C34	121.57(14)

Table 6-1-5 Bond Angles for **1b**.

Atom	Atom	Atom	Angle/°	Atom	Atom	Atom	Angle/°
N1	C1	C2	105.20(12)	C21	C22	C23	117.96(15)
N6	C1	N1	122.86(13)	C20	C19	C18	117.48(15)
N6	C1	C2	130.53(13)	C38	C39	C44	119.47(14)
N5	C24	C23	105.44(12)	C38	C39	C40	121.36(14)
N6	C24	N5	122.64(13)	C40	C39	C44	119.12(15)
N6	C24	C23	130.69(13)	O2	C36	C35	122.37(14)
N1	C8	C7	105.53(12)	O2	C36	C37	114.96(14)
N2	C8	N1	122.35(13)	C35	C36	C37	122.66(14)
N2	C8	C7	130.94(13)	O2	C32	C33	119.81(13)
N3	C16	C15	105.67(12)	C31	C32	O2	118.38(14)
N4	C16	N3	122.60(13)	C31	C32	C33	121.70(15)
N4	C16	C15	129.98(13)	C5	C6	C7	117.77(15)
C44	C35	C34	124.86(13)	C27	C26	C25	121.34(14)
C36	C35	C34	117.71(13)	C27	C28	C33	117.21(14)
C36	C35	C44	117.22(14)	C29	C28	C33	118.49(15)
C7	C2	C1	106.97(12)	C29	C28	C27	124.27(15)
C3	C2	C1	132.35(14)	C26	C27	C28	120.62(14)
C3	C2	C7	120.64(14)	C6	C5	C4	121.50(14)
C18	C23	C24	107.13(12)	C12	C11	C10	117.60(15)
C22	C23	C24	132.56(14)	C3	C4	C5	121.56(15)
C22	C23	C18	120.20(14)	C13	C14	C15	117.43(15)
N5	C17	C18	105.60(12)	C38	C37	C36	119.30(15)
N4	C17	N5	122.45(13)	C43	C42	C41	120.18(16)
N4	C17	C18	130.29(13)	C19	C20	C21	121.64(15)
C2	C7	C8	107.20(12)	C22	C21	C20	121.47(15)
C6	C7	C8	132.16(14)	C32	C31	C30	118.83(16)
C6	C7	C2	120.61(14)	C37	C38	C39	120.78(14)
C25	C34	C35	126.74(13)	C40	C41	C42	119.99(15)
C25	C34	C33	117.56(13)	C41	C40	C39	121.02(15)
C33	C34	C35	115.65(13)	C14	C13	C12	121.87(15)
C10	C15	C16	106.98(12)	C11	C12	C13	121.59(15)
C14	C15	C16	131.98(14)	C29	C30	C31	121.46(14)
C14	C15	C10	120.93(14)	C30	C29	C28	120.61(16)

Table 6-1-5 Bond Angles for **1b**.

Atom	Atom	Atom	Angle/°	Atom	Atom	Atom	Angle/°
C15	C10	C9	107.44(13)	O1	B1	N5	116.36(12)
C11	C10	C15	120.56(14)	O1	B1	N1	112.03(12)
C11	C10	C9	131.88(14)	O1	B1	N3	114.98(12)
N3	C9	C10	105.24(13)	N5	B1	N3	103.71(11)
N2	C9	N3	122.57(13)	N1	B1	N5	104.08(11)
N2	C9	C10	130.66(13)	N1	B1	N3	104.36(11)

Table 6-1-6 Torsion Angles for **1b**.

A	B	C	D	Angle/°	A	B	C	D	Angle/°
O1	C25	C26	C27	174.63(13)	C34	C33	C32	C31	176.55(14)
O2	C36	C37	C38	-175.94(14)	C34	C33	C28	C27	5.2(2)
O2	C32	C31	C30	-173.86(13)	C34	C33	C28	C29	-176.82(14)
N5	C24	C23	C18	5.71(14)	C15	C10	C9	N3	-6.25(15)
N5	C24	C23	C22	-178.17(15)	C15	C10	C9	N2	159.57(15)
N5	C17	C18	C23	-6.68(15)	C15	C10	C11	C12	1.6(2)
N5	C17	C18	C19	178.33(15)	C15	C14	C13	C12	0.5(2)
N1	C1	C2	C7	-7.91(15)	C10	C15	C14	C13	-0.6(2)
N1	C1	C2	C3	174.45(15)	C10	C11	C12	C13	-1.6(2)
N1	C8	C7	C2	7.24(15)	C9	N3	C16	N4	154.54(13)
N1	C8	C7	C6	-174.97(15)	C9	N3	C16	C15	-11.75(16)
N3	C16	C15	C10	7.00(15)	C9	N3	B1	O1	94.87(16)
N3	C16	C15	C14	-176.98(15)	C9	N3	B1	N5	-136.97(13)
N4	C16	C15	C10	-157.90(14)	C9	N3	B1	N1	-28.25(17)
N4	C16	C15	C14	18.1(3)	C9	N2	C8	N1	-7.7(2)
N4	C17	C18	C23	158.57(14)	C9	N2	C8	C7	157.99(14)
N4	C17	C18	C19	-16.4(3)	C9	C10	C11	C12	177.09(15)
N2	C8	C7	C2	-160.28(15)	C43	C44	C39	C38	169.53(13)
N2	C8	C7	C6	17.5(3)	C43	C44	C39	C40	-7.9(2)
N6	C1	C2	C7	158.50(14)	C43	C42	C41	C40	-5.3(2)
N6	C1	C2	C3	-19.1(3)	C44	C35	C34	C25	31.3(2)
N6	C24	C23	C18	-161.55(14)	C44	C35	C34	C33	-151.33(14)
N6	C24	C23	C22	14.6(3)	C44	C35	C36	O2	166.75(13)
C1	N1	C8	N2	155.71(13)	C44	C35	C36	C37	-13.2(2)

Table 6-1-6 Torsion Angles for **1b**.

A	B	C	D	Angle/°	A	B	C	D	Angle/°
C1	N1	C8	C7	-13.15(16)	C44	C43	C42	C41	2.6(2)
C1	N1	B1	O1	95.63(15)	C44	C39	C38	C37	-3.7(2)
C1	N1	B1	N5	-30.91(17)	C44	C39	C40	C41	5.4(2)
C1	N1	B1	N3	-139.35(13)	C18	C23	C22	C21	-1.7(2)
C1	N6	C24	N5	-8.93(19)	C18	C19	C20	C21	-1.4(2)
C1	N6	C24	C23	156.45(14)	C25	O1	B1	N5	-46.75(17)
C1	C2	C7	C8	0.44(15)	C25	O1	B1	N1	-166.31(11)
C1	C2	C7	C6	-177.66(13)	C25	O1	B1	N3	74.76(15)
C1	C2	C3	C4	179.68(15)	C25	C34	C33	C32	161.90(14)
C24	N5	C17	N4	-155.64(13)	C25	C34	C33	C28	-14.2(2)
C24	N5	C17	C18	11.06(15)	C25	C26	C27	C28	-5.3(2)
C24	N5	B1	O1	-93.87(15)	C3	C2	C7	C8	178.41(13)
C24	N5	B1	N1	29.90(16)	C3	C2	C7	C6	0.3(2)
C24	N5	B1	N3	138.83(12)	C33	C34	C25	O1	-165.22(12)
C24	N6	C1	N1	7.8(2)	C33	C34	C25	C26	13.5(2)
C24	N6	C1	C2	-156.50(14)	C33	C32	C31	C30	2.4(2)
C24	C23	C18	C17	0.61(15)	C33	C28	C27	C26	4.7(2)
C24	C23	C18	C19	176.22(13)	C33	C28	C29	C30	-2.2(2)
C24	C23	C22	C21	-177.42(14)	C22	C23	C18	C17	-176.08(13)
C8	N1	C1	N6	-154.33(13)	C22	C23	C18	C19	-0.5(2)
C8	N1	C1	C2	13.39(16)	C19	C20	C21	C22	-0.8(2)
C8	N1	B1	O1	-96.20(15)	C36	O2	C32	C33	23.2(2)
C8	N1	B1	N5	137.27(13)	C36	O2	C32	C31	-160.53(13)
C8	N1	B1	N3	28.82(17)	C36	C35	C34	C25	-154.07(14)
C8	N2	C9	N3	8.2(2)	C36	C35	C34	C33	23.26(18)
C8	N2	C9	C10	-155.56(15)	C36	C35	C44	C43	-161.25(13)
C8	C7	C6	C5	179.90(15)	C36	C35	C44	C39	13.81(19)
C16	N3	C9	N2	-155.86(13)	C36	C37	C38	C39	4.7(2)
C16	N3	C9	C10	11.40(16)	C32	O2	C36	C35	-15.2(2)
C16	N3	B1	O1	-98.00(16)	C32	O2	C36	C37	164.73(13)
C16	N3	B1	N5	30.16(17)	C32	C33	C28	C27	-170.99(14)
C16	N3	B1	N1	138.87(13)	C32	C33	C28	C29	7.0(2)
C16	N4	C17	N5	7.40(19)	C32	C31	C30	C29	2.8(2)

Table 6-1-6 Torsion Angles for **1b**.

A	B	C	D	Angle/°	A	B	C	D	Angle/°
C16	N4	C17	C18	-155.71(14)	C6	C5	C4	C3	0.5(2)
C16	C15	C10	C9	-0.47(16)	C28	C33	C32	O2	168.94(13)
C16	C15	C10	C11	176.05(13)	C28	C33	C32	C31	-7.2(2)
C16	C15	C14	C13	-176.12(15)	C27	C28	C29	C30	175.63(16)
C35	C34	C25	O1	12.1(2)	C11	C10	C9	N3	177.77(15)
C35	C34	C25	C26	-169.17(14)	C11	C10	C9	N2	-16.4(3)
C35	C34	C33	C32	-15.69(19)	C14	C15	C10	C9	-177.02(13)
C35	C34	C33	C28	168.21(13)	C14	C15	C10	C11	-0.5(2)
C35	C44	C39	C38	-5.8(2)	C14	C13	C12	C11	0.5(3)
C35	C44	C39	C40	176.80(13)	C42	C43	C44	C35	179.11(13)
C35	C36	C37	C38	4.0(2)	C42	C43	C44	C39	4.0(2)
C2	C7	C6	C5	-2.6(2)	C42	C41	C40	C39	1.3(2)
C2	C3	C4	C5	-2.7(2)	C31	C30	C29	C28	-2.8(3)
C23	C18	C19	C20	2.0(2)	C38	C39	C40	C41	-171.96(15)
C23	C22	C21	C20	2.3(2)	C40	C39	C38	C37	173.70(15)
C17	N5	C24	N6	157.89(13)	C29	C28	C27	C26	-173.22(15)
C17	N5	C24	C23	-10.65(15)	B1	O1	C25	C34	80.76(16)
C17	N5	B1	O1	96.54(15)	B1	O1	C25	C26	-98.02(15)
C17	N5	B1	N1	-139.69(12)	B1	N5	C24	N6	-12.6(2)
C17	N5	B1	N3	-30.76(16)	B1	N5	C24	C23	178.87(12)
C17	N4	C16	N3	-7.9(2)	B1	N5	C17	N4	14.8(2)
C17	N4	C16	C15	154.76(14)	B1	N5	C17	C18	-178.49(12)
C17	C18	C19	C20	176.42(15)	B1	N1	C1	N6	14.9(2)
C7	C2	C3	C4	2.3(2)	B1	N1	C1	C2	-177.37(12)
C7	C6	C5	C4	2.2(2)	B1	N1	C8	N2	-13.4(2)
C34	C35	C44	C43	13.4(2)	B1	N1	C8	C7	177.71(12)
C34	C35	C44	C39	-171.58(13)	B1	N3	C16	N4	-13.7(2)
C34	C35	C36	O2	-8.3(2)	B1	N3	C16	C15	-179.98(12)
C34	C35	C36	C37	171.78(13)	B1	N3	C9	N2	12.4(2)
C34	C25	C26	C27	-4.2(2)	B1	N3	C9	C10	179.62(12)
C34	C33	C32	O2	-7.3(2)					

Table 6-1-7 Hydrogen Atom Coordinates (Å×10^4) and Isotropic Displacement Parameters (Å^2×10^3) for **1b**.

**Atom**	***x***	***y***	***z***	**U(eq)**
H43	3660.63	6346.89	7681.08	31
H3	6088.46	3214.17	6240.76	33
H22	2884.32	995.34	5819	32
H19	223.57	3326.5	4774.73	34
H6	6279.23	7449.37	5415.33	33
H26	2047.85	8754.69	6456.44	33
H27	627.07	8866.41	6602.92	38
H5	7527.01	6057.54	5670.76	36
H11	3319.1	9923.28	4169.39	37
H4	7435.37	3985.1	6082.24	37
H14	438.2	8010.65	3919.5	36
H37	1967.58	1352.92	7159.62	38
H42	5170.21	6133.74	8238.14	38
H20	233.35	1053.5	4811.85	38
H21	1532.22	-89.68	5333.92	37
H31	-630.6	3716.55	6959.23	39
H38	3523.7	1183.09	7517.07	38
H41	5882.18	4088.68	8310.32	42
H40	5034.61	2244.42	7958.6	40
H13	653.33	9638.82	3221.43	42
H12	2056.01	10589.26	3347.63	43
H30	-1426	5688.48	6890.1	43
H29	-778.28	7627.52	6728.59	42

6-2 **1c**

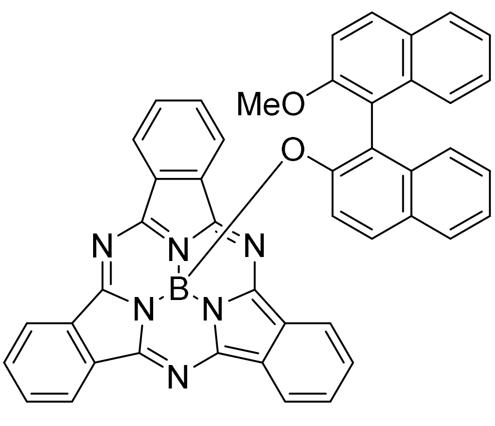

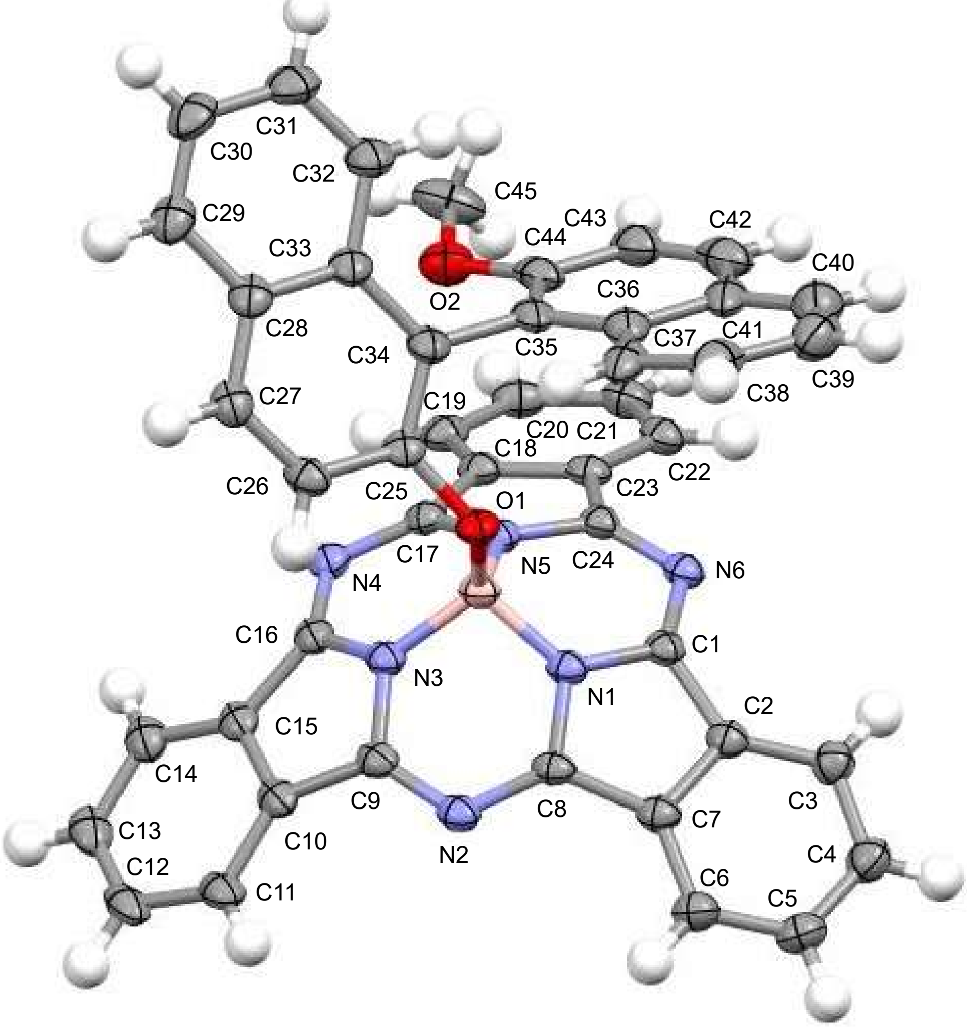

Figure SI-20 Molecular structure of **1c**. Thermal ellipsoids are shown at the 50% probability level.

Table 6-2-1 Crystal data and structure refinement for **1c**.

Identification code	**1c**
Empirical formula	$C_{45}H_{27}BN_6O_2$
Formula weight	694.53
Temperature/K	100.15
Crystal system	monoclinic
Space group	$P2_1/n$
a/Å	12.8291(3)
b/Å	12.2202(2)
c/Å	21.2801(4)
α/°	90
β/°	97.477(2)
γ/°	90
Volume/Å^3	3307.80(11)
Z	4
ρ_{calc}g/cm^3	1.395
μ/mm^{-1}	0.695
F(000)	1440.0
Crystal size/mm^3	0.19 × 0.126 × 0.097
Radiation	Cu Kα (λ = 1.54184)
2Θ range for data collection/°	7.634 to 142.66
Index ranges	-15 ≤ h ≤ 15, -14 ≤ k ≤ 4, -25 ≤ l ≤ 26
Reflections collected	17304
Independent reflections	6227 [R_{int} = 0.0286, R_{sigma} = 0.0327]
Data/restraints/parameters	6227/0/488
Goodness-of-fit on F^2	1.042
Final R indexes [I>=2σ (I)]	R_1 = 0.0670, wR_2 = 0.1852
Final R indexes [all data]	R_1 = 0.0743, wR_2 = 0.1919
Largest diff. peak/hole / e Å^{-3}	1.31/-0.48

Table 6-2-2 Fractional Atomic Coordinates (×10^4) and Equivalent Isotropic Displacement Parameters (Å^2×10^3) for dt13_auto. U_{eq} is defined as 1/3 of the trace of the orthogonalised U_{IJ} tensor.

**Atom**	***x***	***y***	***z***	**U(eq)**
O1	6198.5(14)	4062.6(14)	2667.8(8)	29.0(4)

Table 6-2-2 Fractional Atomic Coordinates (×10^4) and Equivalent Isotropic Displacement Parameters (Å^2×10^3) for dt13_auto. U_{eq} is defined as 1/3 of the trace of the orthogonalised U_{IJ} tensor.

**Atom**	***x***	***y***	***z***	**U(eq)**
N1	5909.5(16)	2833.7(17)	3492.8(9)	25.9(4)
O2	3249.5(16)	4987.5(18)	1575.8(10)	42.0(5)
N5	4425.5(16)	3819.3(17)	3021.5(9)	26.0(4)
N3	5730.3(16)	4701.3(17)	3727.1(9)	26.5(4)
N6	4429.0(16)	1871.5(17)	2981.6(9)	27.0(4)
N4	4029.3(17)	5502.1(17)	3502.5(10)	28.7(5)
N2	6966.2(16)	3602.0(18)	4381.8(10)	29.1(5)
C23	2819.6(19)	3074(2)	2777.5(11)	27.6(5)
C1	5394.7(19)	1884(2)	3322.3(11)	26.1(5)
C24	3942.6(19)	2837(2)	2879.0(11)	26.3(5)
C16	5006(2)	5489(2)	3821.2(11)	27.6(5)
C7	6734.4(19)	1566(2)	4141.4(11)	28.5(5)
C18	2692(2)	4190(2)	2946.1(11)	28.2(5)
C17	3742(2)	4626(2)	3140.6(11)	26.9(5)
C8	6636.6(18)	2736(2)	4018.7(11)	26.3(5)
C2	5954.3(19)	1036(2)	3709.5(12)	28.0(5)
C10	6343(2)	5483(2)	4661.1(12)	30.0(5)
C25	6086(2)	4983(2)	2294.1(11)	28.7(5)
C9	6464.1(19)	4553(2)	4251.7(11)	28.1(5)
C22	1946(2)	2411(2)	2611.0(12)	31.1(5)
C15	5425(2)	6061(2)	4398.6(12)	29.9(5)
C34	5441(2)	4977(2)	1718.3(12)	31.6(6)
C33	5394(2)	5948(2)	1337.8(12)	33.0(6)
C26	6697(2)	5902(2)	2501.3(12)	31.9(6)
C28	5984(2)	6892(2)	1564.8(12)	33.1(6)
C20	838(2)	3959(2)	2783.9(12)	35.6(6)
C35	4798(2)	3983(2)	1496.8(12)	36.5(6)
C27	6646(2)	6840(2)	2150.6(13)	33.5(6)
C6	7378(2)	962(2)	4592.6(13)	33.5(6)
C11	6922(2)	5833(2)	5226.3(12)	36.7(6)
C3	5808(2)	-90(2)	3738.5(13)	33.3(6)
C19	1695(2)	4629(2)	2953.4(12)	32.0(6)

Table 6-2-2 Fractional Atomic Coordinates (×10[4]) and Equivalent Isotropic Displacement Parameters (Å[2]×10[3]) for dt13_auto. U_{eq} is defined as 1/3 of the trace of the orthogonalised U_{IJ} tensor.

**Atom**	***x***	***y***	***z***	**U(eq)**
C21	958(2)	2864(2)	2608.6(13)	35.6(6)
C14	5064(2)	6960(2)	4711.3(13)	36.1(6)
C37	6400(3)	2937(2)	1365.0(13)	41.0(7)
C5	7239(2)	-159(2)	4606.2(13)	37.2(6)
C12	6576(3)	6772(2)	5512.6(12)	43.0(7)
C4	6461(2)	-676(2)	4187.7(14)	38.4(6)
C29	5890(2)	7861(2)	1198.2(14)	38.3(6)
C13	5667(3)	7312(2)	5264.7(13)	40.9(7)
C32	4785(2)	6007(2)	729.8(13)	40.1(7)
C44	3710(2)	4022(2)	1428.7(12)	39.5(7)
C36	5297(3)	3001(2)	1344.0(12)	39.2(7)
C30	5289(3)	7897(2)	626.5(14)	44.0(7)
C41	4635(3)	2061(2)	1134.0(13)	45.9(8)
C31	4731(3)	6953(3)	385.1(14)	46.0(7)
C43	3073(3)	3093(3)	1223.9(14)	45.1(7)
C45	2153(2)	5111(4)	1382.7(14)	53.7(9)
C42	3534(3)	2170(3)	1088.3(14)	48.2(8)
B1	5601(2)	3899(2)	3184.5(13)	26.8(6)
C40	5117(3)	1107(3)	967.3(15)	49.8(8)
C38	6829(3)	1995(3)	1158.5(15)	49.0(8)
C39	6183(3)	1044(3)	967.6(16)	48.0(8)

Table 6-2-3 Anisotropic Displacement Parameters (Å[2]×10[3]) for dt13_auto. The Anisotropic displacement factor exponent takes the form: $-2\pi^2[h^2a^{*2}U_{11}+2hka^*b^*U_{12}+\ldots]$.

**Atom**	U_{11}	U_{22}	U_{33}	U_{23}	U_{13}	U_{12}
O1	34.2(9)	32.7(9)	20.9(8)	3.4(7)	6.5(7)	-1.0(7)
N1	27.5(10)	30.1(11)	20.3(10)	1.8(8)	3.8(8)	-2.0(8)
O2	36.6(10)	51.3(12)	37.6(11)	3.6(9)	3.2(8)	0.9(9)
N5	30.1(10)	28.8(10)	19.3(9)	2.2(8)	4.5(8)	-1.9(8)
N3	31.9(11)	28.3(10)	19.3(9)	2.2(8)	3.8(8)	-5.2(8)
N6	30.1(10)	31.7(11)	19.1(9)	-0.3(8)	2.4(8)	-1.5(8)
N4	34.9(11)	27.9(11)	23.9(10)	4.5(8)	5.9(8)	-1.8(9)

Table 6-2-3 Anisotropic Displacement Parameters ($Å^2×10^3$) for dt13_auto. The Anisotropic displacement factor exponent takes the form: $-2\pi^2[h^2a^{*2}U_{11}+2hka^*b^*U_{12}+…]$.

**Atom**	**U_{11}**	**U_{22}**	**U_{33}**	**U_{23}**	**U_{13}**	**U_{12}**
N2	31.0(11)	34.3(11)	21.9(10)	1.6(8)	3.7(8)	-6.4(9)
C23	30.7(12)	35.9(13)	16.1(11)	2.7(9)	2.4(9)	-0.9(10)
C1	31.1(12)	28.9(12)	19.1(11)	-0.4(9)	5.5(9)	-3.7(10)
C24	30.9(12)	31.0(12)	16.8(11)	0.0(9)	2.5(9)	-3.3(10)
C16	34.8(13)	26.6(12)	22.6(11)	3.6(9)	8.3(10)	-5.1(10)
C7	28.6(12)	34.1(13)	23.2(12)	2.3(10)	4.9(9)	-1.6(10)
C18	32.6(13)	32.6(13)	19.1(11)	4.9(9)	2.7(9)	-1.3(10)
C17	33.3(13)	27.5(12)	20.3(11)	5.8(9)	5.0(9)	0.3(10)
C8	25.1(11)	34.6(13)	19.3(11)	2.9(9)	3.7(9)	-3.2(10)
C2	30.0(12)	30.6(13)	24.2(12)	0.6(10)	6.9(10)	-0.2(10)
C10	38.3(14)	29.7(13)	23.1(12)	2.0(10)	8.7(10)	-11.9(10)
C25	32.7(13)	32.5(13)	22.8(12)	1.8(10)	11.3(10)	-2.2(10)
C9	31.3(12)	32.1(13)	21.4(11)	2.6(10)	5.0(9)	-8.8(10)
C22	33.4(13)	37.5(14)	22.3(12)	-0.8(10)	3.2(10)	-4.4(11)
C15	40.1(14)	27.9(12)	23.5(12)	2.3(10)	10.5(10)	-9.0(10)
C34	42.1(14)	32.9(13)	20.8(12)	-0.7(10)	8.0(10)	-3.7(11)
C33	44.1(15)	34.1(14)	22.4(12)	0.9(10)	10.0(11)	-3.3(11)
C26	32.4(13)	39.6(15)	24.3(12)	-0.9(11)	5.7(10)	-5.9(11)
C28	40.2(14)	33.6(14)	28.3(13)	1.2(10)	15.2(11)	-1.9(11)
C20	29.8(13)	50.3(16)	27.1(13)	4.4(11)	5.1(10)	3.6(11)
C35	59.8(18)	33.5(14)	15.9(11)	2.4(10)	3.8(11)	-7.4(12)
C27	37.2(14)	32.8(14)	32.8(13)	-3.6(11)	13.6(11)	-6.8(11)
C6	32.9(13)	39.0(15)	27.9(13)	4.8(11)	1.1(10)	-0.5(11)
C11	50.9(16)	38.8(15)	20.4(12)	2.9(11)	4.6(11)	-13.9(12)
C3	33.6(13)	32.1(14)	34.3(14)	2.0(11)	5.0(11)	-2.1(10)
C19	37.3(14)	37.0(14)	22.1(12)	3.8(10)	5.9(10)	3.4(11)
C21	31.3(13)	47.2(16)	27.7(13)	0.6(11)	1.9(10)	-6.6(11)
C14	48.1(16)	31.7(13)	31.1(14)	-0.3(11)	15.3(12)	-7.8(12)
C37	70(2)	32.4(14)	24.6(13)	5.7(11)	19.5(13)	7.8(13)
C5	39.0(14)	40.4(15)	31.7(14)	9.4(12)	2.1(11)	2.6(12)
C12	69(2)	41.9(16)	19.0(12)	-3.3(11)	8.5(12)	-21.6(15)
C4	42.1(15)	32.5(14)	40.3(15)	7.4(12)	4.8(12)	-1.5(12)

Table 6-2-3 Anisotropic Displacement Parameters (Å^2×10^3) for dt13_auto. The Anisotropic displacement factor exponent takes the form: $-2\pi^2[h^2a^{*2}U_{11}+2hka^*b^*U_{12}+\ldots]$.

Atom	U_{11}	U_{22}	U_{33}	U_{23}	U_{13}	U_{12}
C29	47.4(16)	33.3(14)	37.9(15)	3.4(12)	19.3(13)	-1.6(12)
C13	62.4(19)	34.4(14)	29.8(14)	-4.8(11)	21.1(13)	-10.6(13)
C32	51.8(17)	42.8(16)	25.9(13)	4.1(12)	5.5(12)	-5.6(13)
C44	49.1(16)	47.7(17)	19.8(12)	4.0(11)	-2.7(11)	-14.7(13)
C36	62.1(19)	35.6(15)	20.7(12)	2.6(11)	9.2(12)	-4.7(13)
C30	59.4(19)	38.6(15)	36.7(15)	12.6(12)	16.8(14)	2.3(13)
C41	87(2)	31.0(14)	20.7(13)	3.2(11)	11.9(14)	1.3(14)
C31	58.2(19)	52.1(18)	27.6(14)	10.2(13)	4.7(13)	-3.2(15)
C43	54.6(18)	48.9(18)	29.8(14)	5.1(13)	-1.7(13)	-10.0(14)
C45	36.3(16)	94(3)	29.3(15)	-4.0(16)	0.3(12)	-4.6(16)
C42	54.8(19)	60(2)	27.7(14)	3.7(13)	-2.8(13)	-16.0(16)
B1	29.2(14)	30.1(14)	20.9(13)	2.0(11)	2.1(10)	-3.0(11)
C40	63(2)	44.8(17)	42.3(17)	0.8(14)	10.0(15)	-2.1(15)
C38	62(2)	49.1(18)	36.3(16)	5.6(13)	9.3(14)	17.2(15)
C39	59(2)	41.7(17)	44.0(17)	4.1(13)	11.0(15)	8.8(14)

Table 6-2-4 Bond Lengths for **1c**.

Atom	Atom	Length/Å	Atom	Atom	Length/Å
O1	C25	1.374(3)	C25	C34	1.386(4)
O1	B1	1.434(3)	C25	C26	1.408(4)
N1	C1	1.361(3)	C22	C21	1.383(4)
N1	C8	1.366(3)	C15	C14	1.394(4)
N1	B1	1.488(3)	C34	C33	1.433(4)
O2	C44	1.374(4)	C34	C35	1.509(4)
O2	C45	1.422(4)	C33	C28	1.429(4)
N5	C24	1.367(3)	C33	C32	1.424(4)
N5	C17	1.365(3)	C26	C27	1.364(4)
N5	B1	1.506(3)	C28	C27	1.416(4)
N3	C16	1.370(3)	C28	C29	1.415(4)
N3	C9	1.375(3)	C20	C19	1.380(4)
N3	B1	1.507(3)	C20	C21	1.402(4)
N6	C1	1.352(3)	C35	C44	1.384(4)

Table 6-2-4 Bond Lengths for **1c**.

Atom	Atom	Length/Å	Atom	Atom	Length/Å
N6	C24	1.339(3)	C35	C36	1.418(4)
N4	C16	1.345(3)	C6	C5	1.382(4)
N4	C17	1.342(3)	C11	C12	1.398(4)
N2	C8	1.345(3)	C3	C4	1.385(4)
N2	C9	1.340(3)	C14	C13	1.390(4)
C23	C24	1.458(3)	C37	C36	1.412(5)
C23	C18	1.425(4)	C37	C38	1.373(4)
C23	C22	1.391(4)	C5	C4	1.399(4)
C1	C2	1.454(3)	C12	C13	1.384(5)
C16	C15	1.454(3)	C29	C30	1.354(4)
C7	C8	1.456(4)	C32	C31	1.367(4)
C7	C2	1.423(3)	C44	C43	1.433(4)
C7	C6	1.394(4)	C36	C41	1.463(4)
C18	C17	1.458(3)	C30	C31	1.417(5)
C18	C19	1.389(4)	C41	C42	1.410(5)
C2	C3	1.392(4)	C41	C40	1.387(5)
C10	C9	1.452(4)	C43	C42	1.323(5)
C10	C15	1.425(4)	C40	C39	1.370(5)
C10	C11	1.396(4)	C38	C39	1.453(5)

Table 6-2-5 Bond Angles for **1c**.

Atom	Atom	Atom	Angle/°	Atom	Atom	Atom	Angle/°
C25	O1	B1	122.1(2)	C14	C15	C16	132.3(3)
C1	N1	C8	113.6(2)	C14	C15	C10	120.8(2)
C1	N1	B1	122.5(2)	C25	C34	C33	118.1(2)
C8	N1	B1	123.5(2)	C25	C34	C35	121.3(2)
C44	O2	C45	117.7(3)	C33	C34	C35	120.6(2)
C24	N5	B1	121.3(2)	C28	C33	C34	119.8(2)
C17	N5	C24	113.1(2)	C32	C33	C34	122.2(2)
C17	N5	B1	124.0(2)	C32	C33	C28	118.0(2)
C16	N3	C9	112.3(2)	C27	C26	C25	120.9(2)
C16	N3	B1	124.0(2)	C27	C28	C33	119.5(2)
C9	N3	B1	122.1(2)	C29	C28	C33	119.1(3)

Table 6-2-5 Bond Angles for **1c**.

Atom	Atom	Atom	Angle/°	Atom	Atom	Atom	Angle/°
C24	N6	C1	116.9(2)	C29	C28	C27	121.4(3)
C17	N4	C16	116.6(2)	C19	C20	C21	121.6(3)
C9	N2	C8	117.2(2)	C44	C35	C34	120.5(3)
C18	C23	C24	107.3(2)	C44	C35	C36	119.0(3)
C22	C23	C24	132.1(2)	C36	C35	C34	120.5(3)
C22	C23	C18	120.4(2)	C26	C27	C28	120.0(2)
N1	C1	C2	105.7(2)	C5	C6	C7	118.2(3)
N6	C1	N1	122.1(2)	C10	C11	C12	117.6(3)
N6	C1	C2	130.3(2)	C4	C3	C2	118.0(3)
N5	C24	C23	105.6(2)	C20	C19	C18	118.1(3)
N6	C24	N5	123.3(2)	C22	C21	C20	120.9(3)
N6	C24	C23	129.0(2)	C13	C14	C15	117.8(3)
N3	C16	C15	106.2(2)	C38	C37	C36	118.5(3)
N4	C16	N3	122.7(2)	C6	C5	C4	121.1(3)
N4	C16	C15	129.6(2)	C13	C12	C11	121.7(3)
C2	C7	C8	107.1(2)	C3	C4	C5	121.6(3)
C6	C7	C8	132.3(2)	C30	C29	C28	121.3(3)
C6	C7	C2	120.5(2)	C12	C13	C14	121.6(3)
C23	C18	C17	106.8(2)	C31	C32	C33	121.0(3)
C19	C18	C23	120.6(2)	O2	C44	C35	117.6(2)
C19	C18	C17	132.4(2)	O2	C44	C43	120.3(3)
N5	C17	C18	106.0(2)	C35	C44	C43	122.1(3)
N4	C17	N5	123.2(2)	C35	C36	C41	118.2(3)
N4	C17	C18	129.0(2)	C37	C36	C35	121.4(3)
N1	C8	C7	105.4(2)	C37	C36	C41	120.4(3)
N2	C8	N1	122.2(2)	C29	C30	C31	120.1(3)
N2	C8	C7	131.0(2)	C42	C41	C36	118.9(3)
C7	C2	C1	107.2(2)	C40	C41	C36	118.5(3)
C3	C2	C1	132.1(2)	C40	C41	C42	122.5(3)
C3	C2	C7	120.6(2)	C32	C31	C30	120.4(3)
C15	C10	C9	107.5(2)	C42	C43	C44	119.2(3)
C11	C10	C9	132.1(3)	C43	C42	C41	122.5(3)
C11	C10	C15	120.4(2)	O1	B1	N1	108.8(2)

Table 6-2-5 Bond Angles for **1c**.

Atom	Atom	Atom	Angle/°	Atom	Atom	Atom	Angle/°
O1	C25	C34	120.9(2)	O1	B1	N5	116.8(2)
O1	C25	C26	117.4(2)	O1	B1	N3	118.8(2)
C34	C25	C26	121.7(2)	N1	B1	N5	104.2(2)
N3	C9	C10	105.6(2)	N1	B1	N3	103.59(19)
N2	C9	N3	122.8(2)	N5	B1	N3	103.0(2)
N2	C9	C10	129.8(2)	C39	C40	C41	121.7(3)
C21	C22	C23	118.4(3)	C37	C38	C39	121.5(3)
C10	C15	C16	106.7(2)	C40	C39	C38	119.2(3)

Table 6-2-6 Torsion Angles for **1c**.

A	B	C	D	Angle/°	A	B	C	D	Angle/°
O1	C25	C34	C33	-177.2(2)	C9	N2	C8	C7	-156.8(2)
O1	C25	C34	C35	3.2(4)	C9	C10	C15	C16	-1.2(3)
O1	C25	C26	C27	178.6(2)	C9	C10	C15	C14	175.8(2)
N1	C1	C2	C7	5.4(3)	C9	C10	C11	C12	-178.8(3)
N1	C1	C2	C3	-178.6(3)	C22	C23	C24	N5	178.8(2)
O2	C44	C43	C42	-179.6(3)	C22	C23	C24	N6	-17.3(4)
N3	C16	C15	C10	-6.7(3)	C22	C23	C18	C17	175.6(2)
N3	C16	C15	C14	176.7(3)	C22	C23	C18	C19	0.7(4)
N6	C1	C2	C7	-158.6(2)	C15	C10	C9	N3	8.7(3)
N6	C1	C2	C3	17.4(5)	C15	C10	C9	N2	-156.1(2)
N4	C16	C15	C10	159.0(2)	C15	C10	C11	C12	-1.1(4)
N4	C16	C15	C14	-17.6(4)	C15	C14	C13	C12	-2.1(4)
C23	C18	C17	N5	5.9(2)	C34	C25	C26	C27	2.4(4)
C23	C18	C17	N4	-158.9(2)	C34	C33	C28	C27	3.0(4)
C23	C18	C19	C20	-1.0(4)	C34	C33	C28	C29	-176.9(2)
C23	C22	C21	C20	-1.5(4)	C34	C33	C32	C31	178.1(3)
C1	N1	C8	N2	-157.3(2)	C34	C35	C44	O2	-0.6(4)
C1	N1	C8	C7	10.5(3)	C34	C35	C44	C43	-179.6(2)
C1	N1	B1	O1	-92.2(3)	C34	C35	C36	C37	-3.0(4)
C1	N1	B1	N5	33.1(3)	C34	C35	C36	C41	179.8(2)
C1	N1	B1	N3	140.6(2)	C33	C34	C35	C44	-65.0(3)
C1	N6	C24	N5	9.4(3)	C33	C34	C35	C36	113.8(3)

Table 6-2-6 Torsion Angles for **1c**.

**A**	**B**	**C**	**D**	**Angle/°**	**A**	**B**	**C**	**D**	**Angle/°**
C1	N6	C24	C23	-152.0(2)	C33	C28	C27	C26	-1.7(4)
C1	C2	C3	C4	-177.1(3)	C33	C28	C29	C30	-2.7(4)
C24	N5	C17	N4	154.7(2)	C33	C32	C31	C30	0.4(5)
C24	N5	C17	C18	-11.2(3)	C26	C25	C34	C33	-1.1(4)
C24	N5	B1	O1	89.2(3)	C26	C25	C34	C35	179.3(2)
C24	N5	B1	N1	-30.8(3)	C28	C33	C32	C31	-2.7(4)
C24	N5	B1	N3	-138.7(2)	C28	C29	C30	C31	0.4(5)
C24	N6	C1	N1	-7.5(3)	C35	C34	C33	C28	178.0(2)
C24	N6	C1	C2	154.3(2)	C35	C34	C33	C32	-2.8(4)
C24	C23	C18	C17	0.8(2)	C35	C44	C43	C42	-0.7(4)
C24	C23	C18	C19	-174.2(2)	C35	C36	C41	C42	0.4(4)
C24	C23	C22	C21	173.9(2)	C35	C36	C41	C40	178.3(3)
C16	N3	C9	N2	152.5(2)	C27	C28	C29	C30	177.4(3)
C16	N3	C9	C10	-13.6(3)	C6	C7	C8	N1	177.0(3)
C16	N3	B1	O1	104.8(3)	C6	C7	C8	N2	-16.7(5)
C16	N3	B1	N1	-134.5(2)	C6	C7	C2	C1	177.8(2)
C16	N3	B1	N5	-26.2(3)	C6	C7	C2	C3	1.2(4)
C16	N4	C17	N5	-8.8(3)	C6	C5	C4	C3	0.9(5)
C16	N4	C17	C18	153.6(2)	C11	C10	C9	N3	-173.4(3)
C16	C15	C14	C13	-179.8(3)	C11	C10	C9	N2	21.8(4)
C7	C2	C3	C4	-1.6(4)	C11	C10	C15	C16	-179.5(2)
C7	C6	C5	C4	-1.3(4)	C11	C10	C15	C14	-2.4(4)
C18	C23	C24	N5	-7.2(2)	C11	C12	C13	C14	-1.4(4)
C18	C23	C24	N6	156.8(2)	C19	C18	C17	N5	-180.0(3)
C18	C23	C22	C21	0.6(4)	C19	C18	C17	N4	15.2(4)
C17	N5	C24	N6	-153.5(2)	C19	C20	C21	C22	1.2(4)
C17	N5	C24	C23	11.6(3)	C21	C20	C19	C18	0.1(4)
C17	N5	B1	O1	-106.5(3)	C37	C36	C41	C42	-176.9(3)
C17	N5	B1	N1	133.5(2)	C37	C36	C41	C40	1.0(4)
C17	N5	B1	N3	25.6(3)	C37	C38	C39	C40	2.9(5)
C17	N4	C16	N3	8.2(3)	C29	C28	C27	C26	178.2(3)
C17	N4	C16	C15	-155.4(2)	C29	C30	C31	C32	0.8(5)
C17	C18	C19	C20	-174.4(2)	C32	C33	C28	C27	-176.3(3)

Table 6-2-6 Torsion Angles for **1c**.

**A**	**B**	**C**	**D**	**Angle/°**	**A**	**B**	**C**	**D**	**Angle/°**
C8	N1	C1	N6	155.5(2)	C32	C33	C28	C29	3.8(4)
C8	N1	C1	C2	-10.1(3)	C44	C35	C36	C37	175.8(2)
C8	N1	B1	O1	96.3(3)	C44	C35	C36	C41	-1.4(4)
C8	N1	B1	N5	-138.4(2)	C44	C43	C42	C41	-0.3(4)
C8	N1	B1	N3	-30.9(3)	C36	C35	C44	O2	-179.5(2)
C8	N2	C9	N3	-7.9(3)	C36	C35	C44	C43	1.6(4)
C8	N2	C9	C10	154.6(2)	C36	C37	C38	C39	-4.8(4)
C8	C7	C2	C1	0.6(3)	C36	C41	C42	C43	0.5(4)
C8	C7	C2	C3	-175.9(2)	C36	C41	C40	C39	-3.0(5)
C8	C7	C6	C5	176.5(3)	C41	C40	C39	C38	1.1(5)
C2	C7	C8	N1	-6.4(3)	C45	O2	C44	C35	167.0(2)
C2	C7	C8	N2	159.9(3)	C45	O2	C44	C43	-14.1(4)
C2	C7	C6	C5	0.2(4)	C42	C41	C40	C39	174.8(3)
C2	C3	C4	C5	0.6(4)	B1	O1	C25	C34	-96.9(3)
C10	C15	C14	C13	4.0(4)	B1	O1	C25	C26	86.8(3)
C10	C11	C12	C13	2.9(4)	B1	N1	C1	N6	-16.7(3)
C25	O1	B1	N1	-179.8(2)	B1	N1	C1	C2	177.6(2)
C25	O1	B1	N5	62.7(3)	B1	N1	C8	N2	14.8(4)
C25	O1	B1	N3	-61.8(3)	B1	N1	C8	C7	-177.4(2)
C25	C34	C33	C28	-1.5(4)	B1	N5	C24	N6	12.4(3)
C25	C34	C33	C32	177.7(3)	B1	N5	C24	C23	177.5(2)
C25	C34	C35	C44	114.5(3)	B1	N5	C17	N4	-10.7(4)
C25	C34	C35	C36	-66.6(3)	B1	N5	C17	C18	-176.6(2)
C25	C26	C27	C28	-0.9(4)	B1	N3	C16	N4	11.9(4)
C9	N3	C16	N4	-154.1(2)	B1	N3	C16	C15	178.8(2)
C9	N3	C16	C15	12.9(3)	B1	N3	C9	N2	-13.7(3)
C9	N3	B1	O1	-90.6(3)	B1	N3	C9	C10	-179.8(2)
C9	N3	B1	N1	30.1(3)	C40	C41	C42	C43	-177.3(3)
C9	N3	B1	N5	138.5(2)	C38	C37	C36	C35	-174.3(3)
C9	N2	C8	N1	7.6(3)	C38	C37	C36	C41	2.8(4)

Table 6-2-7 Hydrogen Atom Coordinates (Å×10^4) and Isotropic Displacement Parameters (Å^2×10^3) for **1c**.

**Atom**	***x***	***y***	***z***	**U(eq)**
H22	2025.99	1664.56	2501.73	37
H26	7150.52	5869.36	2890.94	38
H20	150.84	4246.17	2785.96	43
H27	7055.16	7457.59	2299.46	40
H6	7897.88	1311.29	4883.11	40
H11	7529.03	5447	5409.95	44
H3	5277.3	-446.53	3458.69	40
H19	1604.79	5368.72	3071.81	38
H21	352.05	2428.74	2486.58	43
H14	4428.31	7319.87	4551.48	43
H37	6835.85	3533.36	1518.58	49
H5	7680.06	-586.14	4904.79	45
H12	6974.94	7045.52	5886.79	52
H4	6377.6	-1446.28	4212.34	46
H29	6256.88	8499.76	1356.46	46
H13	5448.73	7937.9	5477.88	49
H32	4411.35	5379.28	561.24	48
H30	5240.59	8555.04	386.8	53
H31	4317.31	6979.32	-18.7	55
H43	2328.13	3139.86	1185.86	54
H45A	1996.94	4965.49	927.33	80
H45B	1940.23	5859.85	1471.18	80
H45C	1765.01	4593.02	1616.66	80
H42	3106.55	1556.58	955.06	58
H40	4697.06	481.91	849.88	60
H38	7564.13	1962.97	1139.92	59
H39	6496.93	386.96	844.57	58

§7 Magnetic Conductive Atomic Force Microscopy (mc-AFM) Experiments

Magnetic conductive atomic force microscopy (mc-AFM) measurements were carried out using a Bruker Multimode 8 equipped with a conductive AFM module.

A solution of 1c (3 mg in 1 mL of toluene) was prepared and filtered through a membrane filter with a pore size of 0.45 µm. The resulting solution was deposited onto a HOPG (Veeco, ZYH) substrate by spin coating (3000 rpm for 30 s). The substrate was then mounted onto the AFM apparatus using a conductive paste (Fujikura Kasei, Dotite).

A Bruker MFMV AFM tip was magnetized for 20 min using a Nd magnet (Niroku Seisakusho Co., Ltd.) with a surface magnetic flux density of 0.49 T. Conductivity measurements were performed at various positions on the substrate, with a total of 40 measurements. After reversing the magnetization direction of the tip, an additional 40 measurements were carried out. The direction of the tip magnetization was defined as follows.

All measured current–voltage (I–V) curves are shown in pale colors, and the averages are plotted as solid lines. The spin polarization percentage (SP%) is presented as a function of the applied bias. The blue line represents the current when the magnet's north pole is oriented upward relative to the substrate, whereas the purple line corresponds to the current when the magnet's north pole is oriented downward.

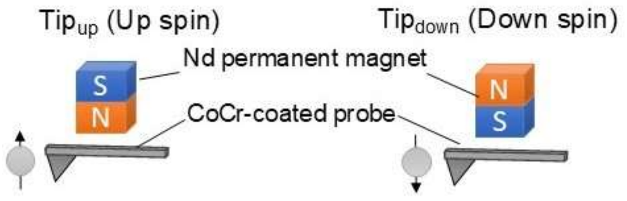

Figure SI-21 Definition of magnetization direction.

$$\mathrm{SP}\ (\%) = \frac{I_{up} - I_{down}}{I_{up} + I_{down}} \times \ 100\%$$

[1] W. H. Pirkle, J. L. Schreiner, *J. Org. Chem.* **1981**, *46*, 4988-4991.
